# Potential efficacy and safety of Xiyanping injection as adjuvant therapy in treatment of suppurative acute tonsillitis: a meta-analysis, trial sequential analysis, and certainty of evidence

**DOI:** 10.3389/fphar.2024.1327856

**Published:** 2024-06-12

**Authors:** Feng-jingming Cheng, Jian Lyu, Lian-xin Wang, Yan-ming Xie

**Affiliations:** ^1^ Institute of Basic Research in Clinical Medicine, China Academy of Chinese Medical Sciences, Beijing, China; ^2^ Xiyuan Hospital, China Academy of Chinese Medical Sciences, Beijing, China

**Keywords:** Xiyanping injection, acute tonsillitis, randomized controlled trial, systematic review, meta-analysis

## Abstract

**Background:** Antibiotic resistance has emerged as a global concern. Xiyanping injection (XYP), a traditional Chinese medicine injection, has been extensively utilized for the treatment of suppurative acute tonsillitis (SAT) in China, exhibiting clinical efficacy. Consequently, there is a need for further evaluation of the potential effectiveness and safety of this treatment. This meta-analysis consolidated data from multiple independent studies to assess the overall treatment efficacy of XYP as adjuvant therapy in patients with SAT.

**Methods:** The search for randomized controlled trials (RCTs) encompassed databases from their inception to 1 April 2024, including the Cochrane Library, PubMed, Embase, SinoMed, CNKI, Wanfang, VIP, and CBM. Data extraction, methodological quality assessment, and meta-analysis were performed independently by two researchers. Review Manager 5.4 was used for data analysis. Various tools were employed for assessment, including forest plots to visualize results, funnel plots to detect publication bias, trial sequential analysis to estimate sample size, and GRADE to evaluate evidence quality.

**Results:** A comprehensive analysis of 32 RCTs involving 4,265 cases was conducted. When compared to conventional treatments (CTs; β-lactams/clindamycin hydrochloride injection/ribavirin) alone, the combination of XYP with CTs demonstrated significant reductions in symptom duration. This included sore throat (MD = −21.08, 95% CI: −24.86 to −17.29, *p* < 0.00001), disappearance of tonsillar redness and swelling (mean difference [MD] = −20.28, 95% confidence interval [CI]: −30.05 to −10.52, *p* < 0.0001), tonsil purulent discharge (MD = −22.40, 95% CI: −28.04 to −16.75, *p* < 0.00001), and normalization of temperature (MD = −19.48, 95% CI: −22.49 to −16.47, *p* < 0.00001). Furthermore, patients receiving CTs combined with XYP exhibited lower levels of interleukin-6 (MD = −7.64, 95% CI: 8.41 to −6.87, *p* < 0.00001) and interleukin-8 (MD = −5.23, 95% CI: −5.60 to −4.86, *p* < 0.00001) than those receiving CTs alone. Additionally, the combination therapy significantly improved the recovery rate (relative risk [RR] = 1.55, 95% CI: 1.37 to 1.77, *p* < 0.00001), white blood cell count recovery rate (RR = 1.13, 95% CI: 1.04 to 1.23, *p* = 0.004), and disappearance rate of tonsillar redness and swelling (RR = 0.51, 95% CI: 1.14 to 1.38, *p* < 0.00001), with no significant increase in adverse events (RR = 0.47, 95% CI: 0.20 to 1.10, *p* = 0.08).

**Conclusion:** The current systematic review and meta-analysis tentatively suggest that the combination of XYP and CTs yields superior clinical outcomes for patients with SAT compared to CTs alone, with a favorable safety profile. Nonetheless, these findings warrant further confirmation through more rigorous RCTs, given the notable heterogeneity and publication bias observed in the included studies.

**Systematic Review Registration:** https://www.crd.york.ac.uk/PROSPERO/display_record.php?RecordID=296118, identifier CRD42022296118.

## 1 Introduction

Acute tonsillitis (AT), accounting for approximately 0.4% of outpatient visits in the United States (Santo and Okeyode, 2022), is characterized by inflammation of the tonsils, situated in the lateral oropharynx between the anterior palatoglossal arch and the posterior palatopharyngeal arch (Sidell and Shapiro, 2012; Georgalas et al., 2014). Common symptoms of AT include sore throat, tonsillar exudate, fever, and tender anterior cervical lymphadenopathy (Gottlieb et al., 2018; Büttner et al., 2023). Clinical treatment methods primarily involve pharmaceuticals and surgery, with antibiotics and anti-inflammatory drugs being extensively utilized. Penicillin and related antibiotics are commonly prescribed for AT treatment. However, antibiotics possess limitations such as a narrow antibacterial spectrum, high antibiotic resistance rates, and limited efficacy in alleviating clinical symptoms. Antibiotic resistance poses an escalating challenge in medical practice, with at least two million individuals in the United States suffering from severe bacterial infections resistant to antibiotics annually (Centers for Disease Control and Prevention U.S. 2013). To address the serious and pressing threat of antibiotic resistance, the United States has implemented initiatives like the *National Action Plan for Combating Antibiotic-Resistant Bacteria* to guide antibiotic usage. Additionally, exploring alternative antibiotics for treating infectious diseases represents an effective strategy (The United States Government, 2020). Notably, research on plant-derived natural products as potential sources of novel antimicrobial agents has garnered considerable attention in recent years (Newman and Cragg, 2020).

In clinical practice, AT is often categorized by clinicians into acute catarrhal tonsillitis (ACT) and suppurative acute tonsillitis (SAT), which pathologically includes acute follicular tonsillitis (AFT) and acute lacunar tonsillitis (ALT). The presence of purulent exudates in the tonsils serves as a distinguishing feature between SAT and ACT. SAT patients typically exhibit yellow or white exudates upon physical examination of the tonsils (Wang, 2003; Huang, 2011). However, there are no precise quantitative diagnostic criteria for AT. According to the Sore Throat (Acute): Antimicrobial Prescribing guidelines from the National Institute for Health and Care Excellence (2018), AT is a self-limiting condition/symptom, with a duration of approximately 1 week, during which most individuals experience improvement without antibiotics, regardless of whether the infection is bacterial or viral. If treatment is deemed necessary, a pharyngeal swab rapid test or culture is recommended (Windfuhr, 2016). SAT is typically more severe than ACT and often warrants antibiotic treatment. Therefore, greater attention should be given to the management of SAT. In Chinese medicine, SAT in children is referred to as “Lan Ru E.” The onset of SAT is rapid and largely attributed to the invasion of the tonsils by “fire” and “heat evil” according to traditional Chinese medicine (TCM) theory. TCM plays an indispensable role in the prevention and treatment of SAT, offering unique advantages. Andrographolide, the primary bioactive compound found in *Andrographis paniculata (A. paniculata)*, demonstrates promising antibacterial activity against most Gram-positive bacteria and is considered a potential candidate for development of new antibacterial drugs (Supplementary Figure S1) (Sareer et al., 2014; Banerjee et al., 2021). Xiyanping injection (XYP), derived from *A. paniculata* (known as Chuan Xin Lian in Chinese, CXL), is a widely used traditional medicinal plant extract. Previous studies have confirmed the safety and efficacy of andrographolide as an anti-inflammatory agent, with beneficial effects on respiratory diseases such as upper respiratory tract infections, lung injury, and pneumonia (Peng et al., 2016; Li et al., 2017; Kumar et al., 2021). Andrographolide reduces the production of pro-inflammatory factors and possesses antibacterial, antiviral, antipyretic, and anti-inflammatory properties (Wang, 2003). Reviews have highlighted the significant anti-inflammatory value and potential applications of andrographolide (Li et al., 2022). Several randomized controlled trials (RCTs) have demonstrated the efficacy of XYP in the treatment of SAT (Qiao et al., 2015; Mai, 2018; Ren, 2018). Moreover, numerous studies have revealed that andrographolide exerts its anti-inflammatory effects through various targets (such as tumor necrosis factor-α and interleukin) and pathways (such as regulation of sirtuin 1/extracellular signal-regulated kinase and modulation of nuclear transcription factor-κB expression and activation) (Gupta et al., 2020; Shi et al., 2020; Cai et al., 2022).

No systematic reviews on XYP for SAT management have been published to date. Therefore, this study reviews the clinical therapeutic effects of XYP on SAT. Through rigorous systematic screening, we identified high-quality articles to enable a comprehensive evaluation of XYP treatment efficacy and safety in SAT patients. Our systematic approach ensures the accuracy and reliability of the assessment, providing valuable insights for clinical application.

## 2 Methods

### 2.1 Study registration

This systematic review was registered in the PROSPERO (registration number: CRD42022296118).

### 2.2 Search strategy

The following databases were systematically searched from their inception to 1 April 2024: CNKI, Wanfang Data, VIP, SinoMed, Cochrane Library, PubMed, Embase, and Web of Science. The search terms used were “Xiyanping” and “tonsillitis.” The literature search was conducted in English, and articles were retrieved regardless of language. Two researchers (Cheng F.J.M. and Lyu J.) independently screened the eligible studies. The detailed retrieval strategies for each database are provided in Supplementary Material S2.

### 2.3 Selection criteria

#### 2.3.1 Types of studies

We included RCTs without restrictions on language or publication type. The participants in these trials had to meet the diagnostic criteria for SAT. We excluded the following types of studies: 1) safety studies; 2) retrospective studies; 3) meta-analyses; 4) mechanism research; 5) reviews; 6) economics research; 7) composition detection; 8) quality control; 9) pharmacodynamics studies; 10) case reports; 11) real-world studies; and 12) protocols.

#### 2.3.2 Patients

Patients diagnosed with suppurative tonsillitis according to the diagnostic criteria of *Otorhinolaryngology Head and Neck Surgery* (Yu, 2004) were included in this study, irrespective of their age, gender, or race. Common symptoms of SAT include sore throat, tonsillar exudate, fever, tender anterior cervical lymphadenopathy, chills, headache, loss of appetite, fatigue, and general malaise.

#### 2.3.3 Types of interventions and comparisons

The experimental group received treatment with XYP alone or XYP in combination with one of the medications used in the control group. The control group was administered conventional treatments (CTs), which comprised β-lactams (such as cefuroxime sodium injection, amoxicillin and potassium clavulanate, benzylpenicillin sodium injection, mezlocillin sodium and sulbactam sodium injection, and cefprozil), ribavirin, azithromycin, and clindamycin hydrochloride injection without XYP. All of these medications have antipyretic and anti-infection effects. The dosage and duration of the treatment were not restricted.

#### 2.3.4 Types of outcomes

The guidelines referenced, such as “*Acute tonsillitis*” (Sidell and Shapiro, 2012) and “*Clinical practice guideline: tonsillitis I. Diagnostics and nonsurgical management*” (Windfuhr, 2016), advocate for the utilization of pharyngeal swab testing to inform antibiotic usage. However, none of the studies included in our analysis documented this outcome. A systematic review highlighted the importance of symptomatic therapies in managing acute tonsillitis to reduce antibiotic usage (Büttner et al., 2023). According to the “*Sore throat (acute): antimicrobial prescribing*” guideline from the National Institute for Health and Care Excellence (2018), AT is typically a self-limiting condition lasting approximately 1 week, with most individuals experiencing improvement without antibiotics, irrespective of the underlying bacterial or viral infection. Considering these international guidelines and the available outcomes extracted from the literature, the primary outcome was the duration of sore throat. Secondary outcomes included the duration of the disappearance of tonsillar redness and swelling, time of tonsil purulent discharge, time of returning to normal temperature, levels of IL-6 and IL-8, recovery rate, recovery rate of the white blood cell count, and disappearance rate of tonsillar redness and swelling. One secondary outcome used to assess the effectiveness of the intervention was the recovery rate, defined as the sum of the percentage of patients classified as “cured,” “improved,” and “significantly improved” (Oncology Center of Excellence, Center for Biologics Evaluation and Research, Center for Drug Evaluation and Research 2018). Safety was evaluated based on the incidence of adverse events (AEs).

#### 2.3.5 Exclusion criteria

Articles were excluded if 1) the full text was not available; 2) data had serious errors; 3) data were repeatedly published, where we retained the first published article; 4) the medications were not intravenous drip; and 5) non-RCTs.

### 2.4 Evaluation method

#### 2.4.1 Literature screening

The study selection process adhered to the guidelines outlined in the PRISMA statement (Supplementary Material S1) (Page et al., 2021). Two researchers (FJMC and JL) independently screened the literature and evaluated the outcomes using standardized information extraction tables. Any disagreements were resolved through discussion or consultation with a third researcher (LXW). NoteExpress software was utilized for managing and revisiting the included articles. Initially, titles and abstracts were scrutinized, followed by a thorough examination of the full text during the final screening phase.

#### 2.4.2 Data extraction

The data collected from each study encompassed the following aspects: 1) title, publication date, author names, and general study information and 2) characteristics of the included studies, including sample size, allocation of cases in each group, details of interventions and control measures, treatment duration, efficacy parameters, and safety indicators. These details were systematically organized into a custom-designed data extraction form. In instances where any data were incomplete or unavailable, efforts were made to contact the authors for clarification and supplementation.

#### 2.4.3 Risk of bias

The methodological quality of the studies was assessed independently by two researchers, FJMC and JL, with any disagreements resolved through consultation with a third researcher, LXW. The assessment was conducted using the Cochrane risk-of-bias 2.0 (RoB2.0) tool, which evaluates various aspects including the randomization process, deviations from intended interventions, missing outcome data, measurement of outcomes, and selection of reported results. Each study was categorized as having a low risk of bias, high risk of bias, or some concerns in each of these five domains. Additionally, the articles were rigorously evaluated based on the PRISMA statement guidelines to ensure comprehensive reporting of the study findings.

#### 2.4.4 Data synthesis and analysis

The statistical analysis was conducted using RevMan 5.4.1 software. To assess statistical heterogeneity, the I^2^ test was employed, with values of I^2^ ≤ 50% indicating low heterogeneity and values of I^2^ > 50% indicating substantial heterogeneity. Based on the level of heterogeneity, either a fixed-effects model or a random-effects model was utilized. Subgroup analysis was performed to investigate potential sources of heterogeneity. For count variables, the relative risk (RR) was calculated, while the mean difference (MD) was used for continuous variables. All analyses were reported with 95% confidence intervals (CIs). In cases where data were not suitable for meta-analysis, a descriptive analysis was conducted.

#### 2.4.5 Subgroup analysis

Subgroup analysis was conducted to reduce heterogeneity among studies, focusing on each outcome indicator and categorized by different interventions and treatment durations (3–7 days and 8–14 days) to enhance result accuracy. The main interventions analyzed included XYP plus β-lactams versus β-lactams alone, XYP plus cefuroxime sodium injection versus cefuroxime sodium injection alone, XYP plus clindamycin hydrochloride injection versus clindamycin hydrochloride injection alone, XYP plus ribavirin versus ribavirin alone, and XYP plus azithromycin versus azithromycin alone. Each outcome underwent subgroup analysis for mitigating inter-study heterogeneity.

#### 2.4.6 Sample size estimate

Trial sequential analysis (TSA) is a valuable method for estimating sample sizes to ensure the statistical robustness of meta-analyses (Brok et al., 2008). When the number of included cases exceeds the predetermined threshold determined by the TSA, the reliability of the results is considered high.

#### 2.4.7 Certainty of evidence

FJMC and JL independently evaluated the certainty of evidence for each outcome using the GRADE method, following the criteria outlined in the Cochrane Systematic Review Manual (Balshem et al., 2011). GRADEpro software was employed for this assessment, considering factors such as the random allocation method, allocation concealment, blinding methods, patient attrition, outcome integrity, and other biases. The certainty of evidence for each outcome was graded as high, moderate, low, or very-low level according to the GRADE guidelines (Guyatt et al., 2011).

## 3 Results

### 3.1 Search results and study characteristics

**FIGURE 1:**
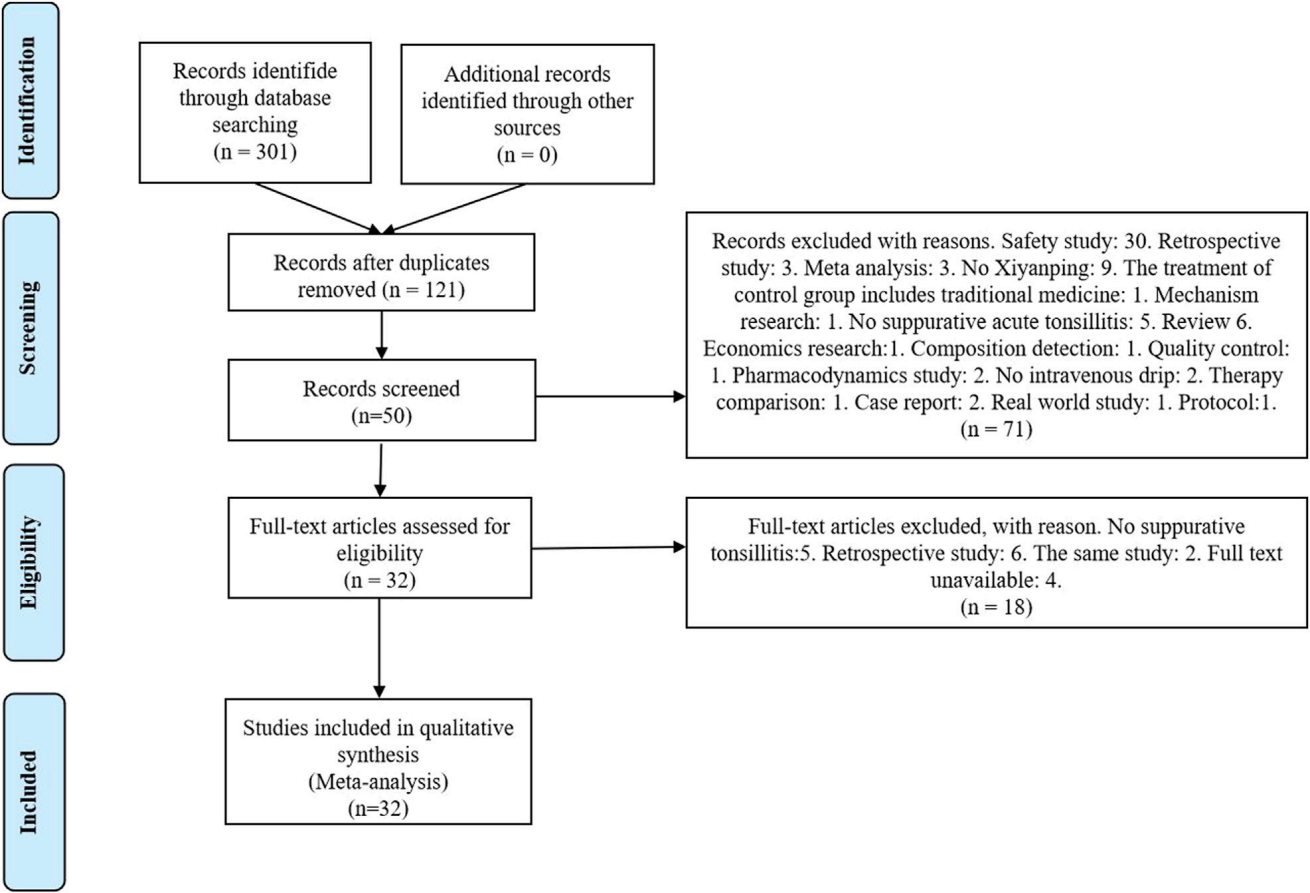
Flowchart of including and excluding studies.

The initial search strategy yielded a total of 288 studies, sourced from various databases including 74 from CNKI, 109 from Wanfang, 44 from VIP, 69 from SinoMed, 1 from PubMed, 2 from the Cochrane Library, 1 from Web of Science, and 1 from Embase. Following the removal of 118 duplicate studies, an additional 71 studies were excluded after abstract review. Subsequently, 18 studies were excluded after full-text review, leaving a final selection of 32 RCTs for inclusion (Li and Li, 2004; Dai, 2006; Liu et al., 2008; Dong, 2009; Lu, 2010; Zou, 2010; Ye, 2011; Jiang and Yang, 2012; He, 2013; Wu, 2013; Long and Cai, 2014; Pan et al., 2014; Peng et al., 2015; Qiao et al., 2015; Wang, 2015; Luo, 2016; Li, 2017; Ou et al., 2017; Hu et al., 2018; Mai, 2018; Ren, 2018; Shi, 2018; Shuai et al., 2018; Zeng, 2018; Zhou, 2018; Zhou, 2018; Gu, 2019; Zhou, 2019; Gan et al., 2020; Gan et al., 2020; Gong, 2020; Liu, 2020; Lyu, 2020; Sun, 2020), involving a total of 4,265 cases (Figure 1). The screening process flowchart is depicted in Figure 1. Data were extracted independently by two researchers, with 2,211 and 2,054 cases allocated to the treatment and control groups, respectively. Notably, all 32 RCTs were published in China, with publication dates ranging from 2004 to 2020. Fifteen of the studies reported adverse drug reactions (ADRs) (Liu et al., 2008; Dong, 2009; Lu, 2010; Zou, 2010; He, 2013; Long and Cai, 2014; Peng et al., 2015; Qiao et al., 2015; Wang, 2015; Ou et al., 2017; Mai, 2018; Ren, 2018; Shuai et al., 2018; Liu, 2020; Sun, 2020). Consistency in baseline characteristics was observed across all studies. The detailed characteristics of the included studies are summarized in Table 1.

**TABLE 1:** Characteristics of included studies.

Study year region	Type	Cases			Gender (male/female)		Age/year		Baseline	Intervention		Dosage	Outcomes	AEs	
		T	C	Total	T	C	T	C		T	C			T	C
Qiao et al. (2015)	RCT	65	61	126	36/29	35/26	0.83 ∼ 14 (3.96 ± 2.37)	0.83 ∼ 14 (4.05 ± 2.51)	B	A (5 mg/kg/d, qd, i.v.) + CT (C)	CT (C)	7 ∼ 10 d	①③④⑦⑩	One case of vomiting; one case of chills and fever	One case of skin rash; one case of abdominal pain and vomiting
Mai (2018)	RCT	38	37	75	19/19	18/19	0.5 ∼ 9 (4.75 ± 0.15)	0.5 ∼ 9 (4.75 ± 0.17)	B	A (5 ∼ 15 mg/kg/d, qd, i.v.) + CT (F, qd, i.v.)	CT (F, qd, i.v.)	NA	①②③④⑦⑩	One case	Three cases
Ren (2018)	RCT	47	47	94	24/23	25/22	1 ∼ 11 (5.32 ± 2.21)	1 ∼ 10 (5.28 ± 2.14)	B	A (250 mg, qd, i.v.) + CT (F, 15 mg/kg/d, qd, i.v.)	CT (F, 15 mg/kg/d, qd, i.v.)	3 d	⑦⑩	One case of diarrhea; two cases of itching	Six cases of nausea; four cases of vomiting; four cases of diarrhea
Liu (2020)	RCT	43	43	86	22/21	20/23	2 ∼ 10 (6.01 ± 0.77)	2 ∼ 9 (5.67 ± 0.82)	B	A (5 mg/kg, qd, i.v.) + CT (C2, 30 mg/kg, tid, i.v.)	CT (C2, 30 mg/kg, tid, i.v.)	5 d	①②③⑤⑩	Two cases of vomiting; two cases of diarrhea; two cases of rash	One case of vomiting; one case of diarrhea; two cases of rash
Lu (2010)	RCT	60	60	120	65/55		2–11 (6.1)		B	A (5 ∼ 15 mg/kg/d, qd, i.v.) + CT (C2, 30 mg/kg, tid, i.v.)	CT (C2, 30 mg/kg, tid, i.v.)	7 d	⑦	0 case	0 case
Ye (2011)	RCT	60	60	120	32/28	34/26	0 ∼ 18		B	A (0.2 ∼ 0.4 mL/kg·d, i.v.) + CT (C3, 20 ∼ 300,000 U/kg·d, bid, i.v.)	CT (C3, 20 ∼ 300,000 U/kg·d, bid, i.v.)	5 ∼ 7 d	⑦	NA	NA
Lyu (2020)	RCT	60	60	120	68/52		2 ∼ 13 (6.04 ± 3.28)		B	A (5 ∼ 10 mg/kg/d, qd, i.v.) + CT (C, i.v.)	CT (C, i.v.)	5 d	①②⑦	NA	NA
Wu (2013)	RCT	60	60	120	38/22	34/26	(4.8 ± 1.5)	(4.3 ± 1.8)	B	A (0.2–0.4 mL/kg/d, i.v.) + CT (C4, 75 mg/kg/time, bid, i.v.)	CT (C4, 75 mg/kg/time, bid, i.v.)	5 d	①④⑦⑧	NA	NA
Zhou (2019)	RCT	60	60	120	32/28	34/26	2 ∼ 10 (5.3 ± 2.2)	1 ∼ 11 (5.4 ± 2.1)	B	A (5 ∼ 10 mg/kg/d, qd, i.v.) + CT (F, 25 ∼ 40 mg/kg, qd, i.v.)	CT (F, 25 ∼ 40 mg/kg, qd, i.v.)	7 d	①②③④⑦	NA	NA
Sun (2020)	RCT	254	105	359	185/69	73/32	4 ∼ 16 (11.06 ± 2.57)	4 ∼ 17 (11.15 ± 2.62)	B	A (5 ∼ 10 mg/kg/d, qd, i.v.) + CT (C5, 7.5 mg/(kg. d), bid, po)	CT (C5, 7.5 mg/(kg. d), bid, po)	14 d	①③④⑦⑩	Four cases of somnolence; three cases of rash; four of nausea and vomiting	Eleven cases of somnolence; 8 cases of rash; 11 cases of nausea and vomiting
Shuai et al. (2018)	RCT	54	54	108	35/19	32/22	2 ∼ 9 (4.2 ± 1.0)	2 ∼ 11 (4.7 ± 1.4)	B	A (5 mg/kg/d, qd, i.v.) + CT (F, 5 mg/kg, bid, i.v.)	CT (F, 5 mg/kg, bid, i.v.)	7 d	①④⑤⑥⑦⑩	Two cases of nausea and vomiting	10 cases
Peng et al. (2015)	RCT	50	50	100	28/22	27/23	1 ∼ 11 (5.2 ± 2.1)	1 ∼ 10 (5.5 ± 2.3)	B	A (5 ∼ 10 mg/kg/d, qd, i.v.) + CT (F, 25 ∼ 40 mg/kg, qd, i.v.)	CT (F, 25 ∼ 40 mg/kg, qd, i.v.)	3 d	①②③④⑤⑥⑦⑩	0 case	0 case
Dai (2006)	RCT	34	34	68	19/15	22/12	0 ∼ 18		B	A (0.2–0.4 mL/kg/d, qd, i.v.) + CT (C3, 200,000 U/kg·d, bid, i.v.)	CT (C3, 200,000 U/kg·d, bid, i.v.)	3 ∼ 5 d	⑦	NA	NA
Zeng (2018)	RCT	43	43	86	24/19	25/18	0.92 ∼ 6 (3.13 ± 1.04)	0.83 ∼ 5 (3.01 ± 1.03)	B	A (0.2–0.4 mL/kg/d, i.v.) + CT (D, qd, i.v.)	CT (D, qd, i.v.)	5 d	④	NA	NA
Li and Li (2004)	RCT	60	66	126	34/26	35/31	0 ∼ 18		B	A (0.2–0.4 mL/kg/d, i.v.) + CT (C3, 20 ∼ 300,000 U/kg·d, bid, i.v.)	A (0.2–0.4 mL/kg/d, i.v.) + CT (C3, 20 ∼ 300,000	5 ∼ 7 d	⑦⑨	NA	NA
Li (2017)	RCT	54	54	108	28/26	30/24	1 ∼ 13 (6.3 ± 1.5)	1 ∼ 12 (5.9 ± 1.3)	B	A (5 ∼ 10 mg/kg/d, qd, i.v.) + CT (C1, 100 mg/kg, bid, i.v.)	CT (C1, 100 mg/kg, bid, i.v.)	7 d	③④	NA	NA
Jiang and Yang (2012)	RCT	38	38	76	20/18	22/16	18 ∼ 65	18 ∼ 65	B	A (250 mg, qd, i.v.) + CT (C)	CT (C)	5 d	①④⑦⑧⑨	NA	NA
Pan et al. (2014)	RCT	60	60	120	34/26	29/31	2 ∼ 13		B	A (5 ∼ 10 mg/kg/d, qd, i.v.) + CT (E, 10 mg/kg/d, i.v.)	CT (E, 10 mg/kg/d, i.v.)	5 d	①④⑦⑧⑨	NA	NA
Wang (2015)	RCT	39	39	78	22/17	23/16	1 ∼ 9 (5.19 ± 2.34)	1 ∼ 10 (5.25 ± 2.17)	B	A (0.2∼0.4 mL/kg/d, i.v.) + CT (C, i.v.)	CT (C, i.v.)	7 d	⑤⑥⑦	0 case	0 case
Shi (2018)	RCT	450	450	900	246/204	249/201	0.33 ∼ 12 (4.88 ± 0.34)	0.25 ∼ 13 (4.98 ± 0.35)	B	A (0.2∼0.4 mL/kg/d, i.v.) + CT (C, i.v.)	CT (C, i.v.)	5 d	①②③④⑦	NA	NA
Luo (2016)	RCT	49	49	98	29/20	30/19	(4.5 ± 2.1)	(4.6 ± 2.1)	B	A (5 ∼ 10 mg/kg/d, qd, i.v.) + CT (F, 8 mg/kg, qd, i.v.)	CT (F, 8 mg/kg, qd, i.v.)	7 d	⑤⑥⑦	NA	NA
Hu et al. (2018)	RCT	54	54	108	28/26	31/23	2 ∼ 10 (5.23 ± 2.04)	1 ∼ 11 (5.41 ± 2.14)	B	A (5 ∼ 10 mg/kg/d, qd, i.v.) + CT (F, 25 ∼ 40 mg/kg/d, qd, i.v.)	CT (F, 25 ∼ 40 mg/kg/d, qd, i.v.)	7 d	①②③④⑤⑥⑦	NA	NA
Dong (2009)	RCT	42	40	82	23/19	21/19	7.5	7.8	B	A (5 ∼ 10 mg/kg/d, i.v.) + CT (F, 25 ∼ 40 mg/kg/d, i.v.)	CT (F, 25 ∼ 40 mg/kg/d, i.v.)	5 ∼ 7 d	⑦	0 case	0 case
He (2013)	RCT	57	57	114	31/26	32/25	1 ∼ 8 (4.8 ± 2.3)	1 ∼ 8 (4.7 ± 2.2)	B	A (5 ∼ 10 mg/kg/d, qd, i.v.) + CT (F, 5 ∼ 8 mg/kg/d, tid, i.v.)	CT (F, 5 ∼ 8 mg/kg/d, tid, i.v.)	7 d	⑦⑩	One case of venous inflammation	0 case
Zou (2010)	RCT	30	30	60	19/11	18/12	0 ∼ 18		B	A (0.2∼0.4 mL/kg/d, qd, i.v.) + CT (F, 200,000 U/kg·d, bid, i.v.)	CT (F, 200,000 U/kg·d, bid, i.v.)	3 ∼ 5d	⑦	0 case	0 case
Gu (2019)	RCT	45	45	90	23/22	22/23	(4.3 ± 1.4)	(4.5 ± 1.2)	B	A (10 mg/kg/d, qd, i.v.) + CT (F, 5 mg/kg, tid, i.v.)	CT (F, 5 mg/kg, tid, i.v.)	7 d	⑤⑥⑦	NA	NA
Long and Cai (2014)	RCT	30	30	60	35/25		0.25 ∼ 14		B	A (0.2 mL/kg/d, qd, i.v.) + CT (C, 75 mg/kg, bid, i.v.)	CT (C, 75 mg/kg, bid, i.v.)	NA	①④⑦⑧⑩	Two cases of rash; one case of diarrhea. The symptoms improved after symptomatic support	Two cases of mild rash and nausea. The symptoms improved after symptomatic support
Gong (2020)	RCT	62	62	124	40/22	38/24	1 ∼ 7 (4.92 ± 0.81)	2 ∼ 8 (5.01 ± 0.42)	B	A (2 ∼ 4 mL/time, i.v.) + CT (D, 10 ∼ 15 mg/kg, i.v.)	CT (D, 10 ∼ 15 mg/kg, i.v.)	3 ∼ 7 d	①②③④⑦	NA	NA
Ou et al. (2017)	RCT	100	100	200	53/47	51/49	1 ∼ 14 (4.77 ± 2.13)	1 ∼ 14 (4.71 ± 2.38)	B	A (5 ∼ 10 mg/kg/d, qd, i.v.) + CT (F, 5 ∼ 8 mg/kg, tid, i.v.)	CT (F, 5 ∼ 8 mg/kg, tid, i.v.)	5 d	①②③④⑦	0 case	0 case
Liu et al. (2008)	RCT	36	33	69	20/16	18/15	0 ∼ 18		B	A (5 ∼ 10 mg/kg, qd, i.v.) + CT (C3, 200,000 U/kg·d, bid, i.v.)	A (5 ∼ 10 mg/kg, qd, i.v.) + CT (C3, 200,000 U/kg·d, bid, i.v.)	3 ∼ 5 d	⑦	0 case	0 case
Zhou (2018)	RCT	47	43	90	27/20	25/18	1–11 (5.18 ± 2.65)	1–12 (5.46 ± 2.39)	B	A (5 ∼ 10 mg/kg, qd, i.v.) + CT (C3, 100 ∼ 300 万U/kg·d, bid, i.v.)	CT (C3, 100 ∼ 300 万U/kg·d, bid, i.v.)	NA	①④⑦⑧⑨	NA	NA
Gan et al. (2020)	RCT	30	30	60	NA	NA	2 ∼ 8 (5.10 ± 0.34)	2 ∼ 9 (5.24 ± 0.51)	B	A (2 ∼ 4 mL/time, i.v.) + CT (D, 10 ∼ 15 mg/kg, i.v.)	CT (D, 10 ∼ 15 mg/kg, i.v.)	3 ∼ 7 d	⑦	NA	NA

Notes: T: treatment group; C: control group; B: balanced; A: Xiyanping injection; CT: conventional treatment [C: β-lactams (C1: cefuroxime sodium for injection; C2: amoxicillin and potassium clavulanate; C3: benzylpenicillin sodium for injection; C4: mezlocillin sodium and sulbactam sodium for injection; C5: cefprozil); D: ribavirin; E: azithromycin; F: clindamycin hydrochloride injection]. ①: Duration of sore throat; ②: duration of tonsil redness and swelling; ③: duration of purulent secretion; ④: time to fever resolution; ⑤: IL-6; ⑥: IL-8; ⑦: recovery rate; ⑧: white blood cell count normalization rate; ⑨: restoration rate of tonsil enlargement; and ⑩: incidence of adverse events [AEs]; NA: not applicable.

### 3.2 Methodological quality assessment

**FIGURE 2:**
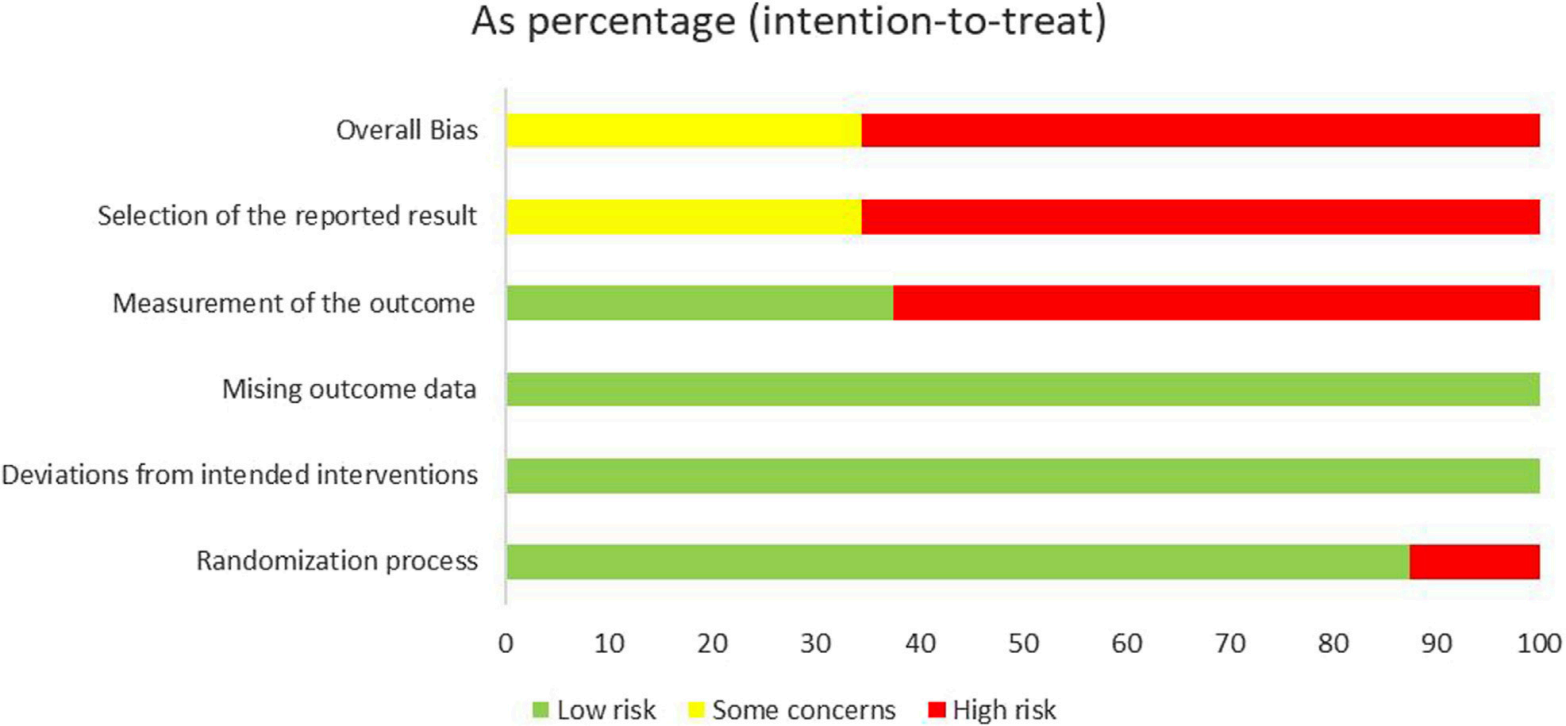
Risk of bias among included studies.

All of the included studies were RCTs, with 13 trials (Liu et al., 2008; Dong, 2009; Peng et al., 2015; Qiao et al., 2015; Luo, 2016; Li, 2017; Ou et al., 2017; Hu et al., 2018; Mai, 2018; Ren, 2018; Shi, 2018; Liu, 2020; Sun, 2020) providing detailed descriptions of their randomization methods. Among these, one trial (Sun, 2020) utilized stratified randomization, while eight trials (Peng et al., 2015; Qiao et al., 2015; Luo, 2016; Li, 2017; Ou et al., 2017; Ren, 2018; Shi, 2018; Liu, 2020) employed random number tables. Additionally, three trials (Hu et al., 2018; Mai, 2018; Gan et al., 2020) employed treatment modalities as their randomization method. Notably, three studies categorized participants based on treatment modalities, while one study (Dong, 2009) utilized an odd or even admission number sequence for randomization. In this particular study, participants were allocated to groups based on whether their admission number was odd or even. However, this method was deemed to pose a high risk of bias. It is worth mentioning that none of the remaining studies provided information on allocation concealment or blinding procedures. Additionally, none of the 32 studies reported research protocols, sample size estimation, or concealed information regarding random allocation plans. Therefore, these studies were rated as having an unclear risk of bias (Figure 2).

**FIGURE 3:**
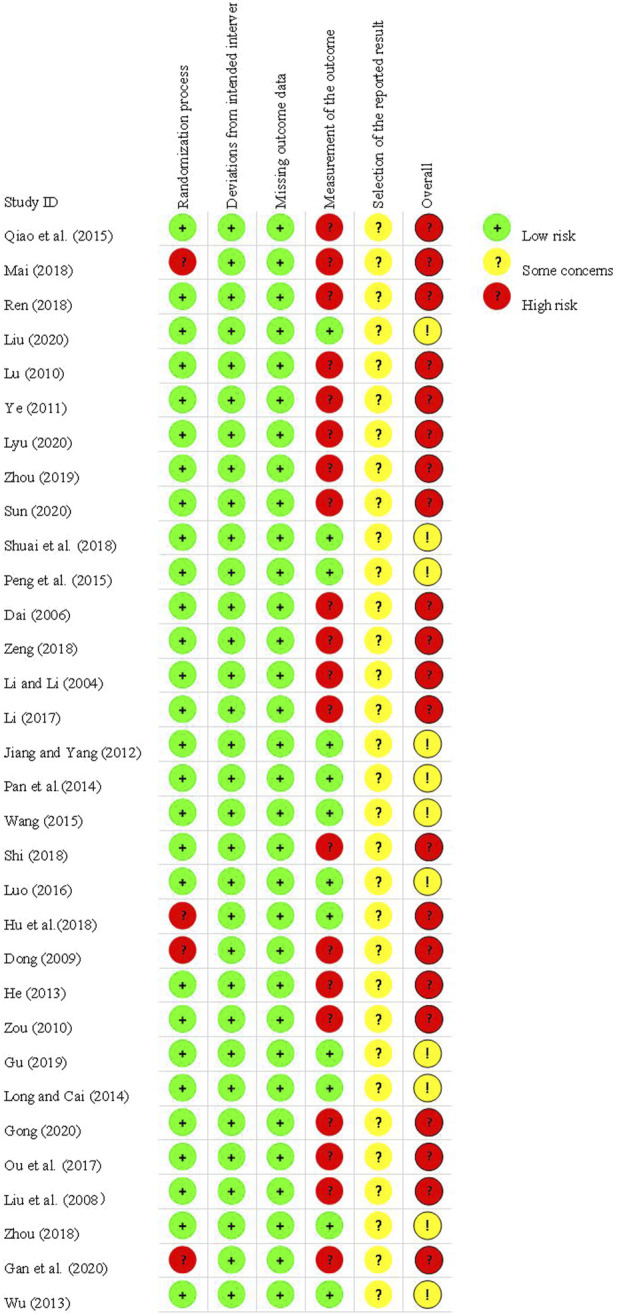
Assessment of the risk of bias in 32 trials.

Six studies (Peng et al., 2015; Hu et al., 2018; Mai, 2018; Zhou, 2019; Gan et al., 2020; Liu, 2020) successfully passed ethical review processes. In 12 studies (Peng et al., 2015; Li, 2017; Ou et al., 2017; Hu et al., 2018; Mai, 2018; Ren, 2018; Zhou, 2019; Gan et al., 2020; Gong, 2020; Liu, 2020; Lyu, 2020; Sun, 2020), patients provided informed consent prior to participation. Notably, none of the studies reported any instances of patient dropout. However, in one study (Mai, 2018), the outcome indicator detection results failed to specify the number of samples in each group. This lack of detail renders it difficult to assess the completeness of the data and the level of uncertainty regarding bias risk (Figure 3).

### 3.3 Primary outcome

#### 3.3.1 Duration of sore throat

**FIGURE 4:**
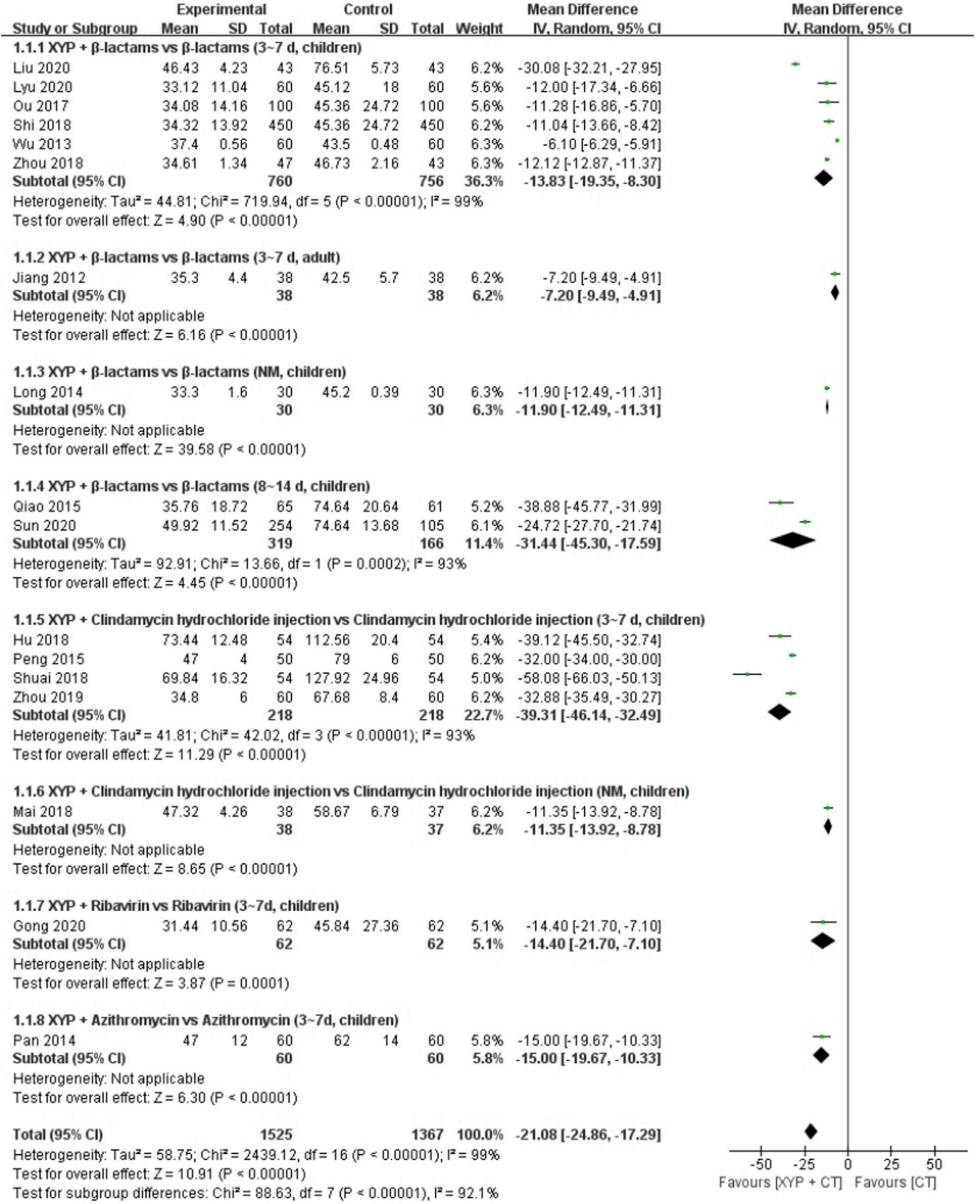
Forest plot of the duration of sore throat.

Seventeen studies (Jiang and Yang, 2012; Wu, 2013; Long and Cai, 2014; Pan et al., 2014; Peng et al., 2015; Qiao et al., 2015; Ou et al., 2017; Hu et al., 2018; Mai, 2018; Shi, 2018; Shuai et al., 2018; Zhou, 2018; Zhou, 2019; Gong, 2020; Liu, 2020; Lyu, 2020; Sun, 2020) comprising 2,892 cases reported the duration of sore throat. Notably, the heterogeneity among these studies was high (I^2^ = 99%, *p* < 0.00001). Consequently, a random-effects model was employed for data analysis. The forest plot depicted significant differences between the treatment and control groups (MD = −21.08, 95% CI: −24.86 to −17.29, *p* < 0.00001; Figure 4).

To address the heterogeneity observed, we conducted subgroup analyses based on the combination medication, duration of treatment, and age of the patients. Specifically, 6 studies (Wu, 2013; Ou et al., 2017; Shi, 2018; Zhou, 2018; Liu, 2020; Lyu, 2020) compared the efficacy of XYP plus β-lactams versus β-lactams alone over a treatment duration of 3–7 days in children. Notably, significant heterogeneity was evident among these studies (*p* < 0.00001, I^2^ = 99%). Utilizing a random-effects model, the analysis revealed that XYP plus β-lactams (3–7 days, children) was more effective than β-lactams alone (3–7 days, children) in reducing the duration of sore throat (MD = −13.83, 95% CI: −19.35 to −8.30, *p* < 0.00001; Figure 4). Of note, the confidence intervals of two trials (Wu, 2013; Liu, 2020) did not overlap with those of the other four trials in the forest plot. After reviewing the original study, it was noted that one trial (Liu, 2020) had fewer than 90 cases, while another trial (Wu, 2013) did not specify the outcome measures in advance. These factors were deemed significant contributors to the observed heterogeneity (*p* = 0.88, I^2^ = 0%). Therefore, these two trials were excluded from the analysis. The remaining four trials (Ou et al., 2017; Shi, 2018; Zhou, 2018; Lyu, 2020) were pooled using a fixed-effects model, revealing a significant reduction in sore throat duration (MD = −12.03, 95% CI: −12.73 to −11.32, *p* < 0.000 01; Supplementary Figure S2). In addition, two studies (Qiao et al., 2015; Sun, 2020) compared XYP plus β-lactams (8–14 days, children) with β-lactams alone (8–14 days, children), demonstrating significant heterogeneity among studies (*p* = 0.0002, I^2^ = 93%). Nonetheless, XYP plus β-lactams (8–14 days, children) was found to be superior in reducing sore throat duration (MD = −31.44, 95% CI: −45.30 to −17.59, *p* < 0.00001; Figure 4). Furthermore, four studies (Peng et al., 2015; Hu et al., 2018; Shuai et al., 2018; Zhou, 2019) investigated the efficacy of XYP plus clindamycin hydrochloride injection (3–7 days, children) compared to clindamycin hydrochloride injection alone (3–7 days, children), revealing significant heterogeneity among studies (*p* < 0.00001, I^2^ = 93%). Upon exclusion of the trial conducted by Shuai et al. (2018) due to lack of informed consent and ethical approval, the pooled analysis of the remaining three trials (Peng et al., 2015; Hu et al., 2018; Zhou, 2019) showed a significant reduction in sore throat duration (MD = −32.72, 95% CI: −34.26 to −31.18, *p* < 0.000 01; Supplementary Figure S2).

The results from the other three studies supported significant differences in sore throat duration when comparing XYP plus the combination of β-lactams (3–7 days, adult) (Jiang and Yang, 2012) (MD = −7.20, 95% CI: −9.49 to −4.91, *p* < 0.00001), ribavirin (3–7 days, children) (Pan et al., 2014) (MD = −14.40, 95% CI: −21.70 to −7.10, *p* = 0.0001), and azithromycin (3–7 days, children) (Gong, 2020) (MD = −15.00, 95% CI: −19.67 to −10.33, *p* < 0.00001) compared to the control group. Additional details are given in Figure 4.

### 3.4 Secondary outcomes

#### 3.4.1 The duration of disappearance of tonsillar redness and swelling

**FIGURE 5:**
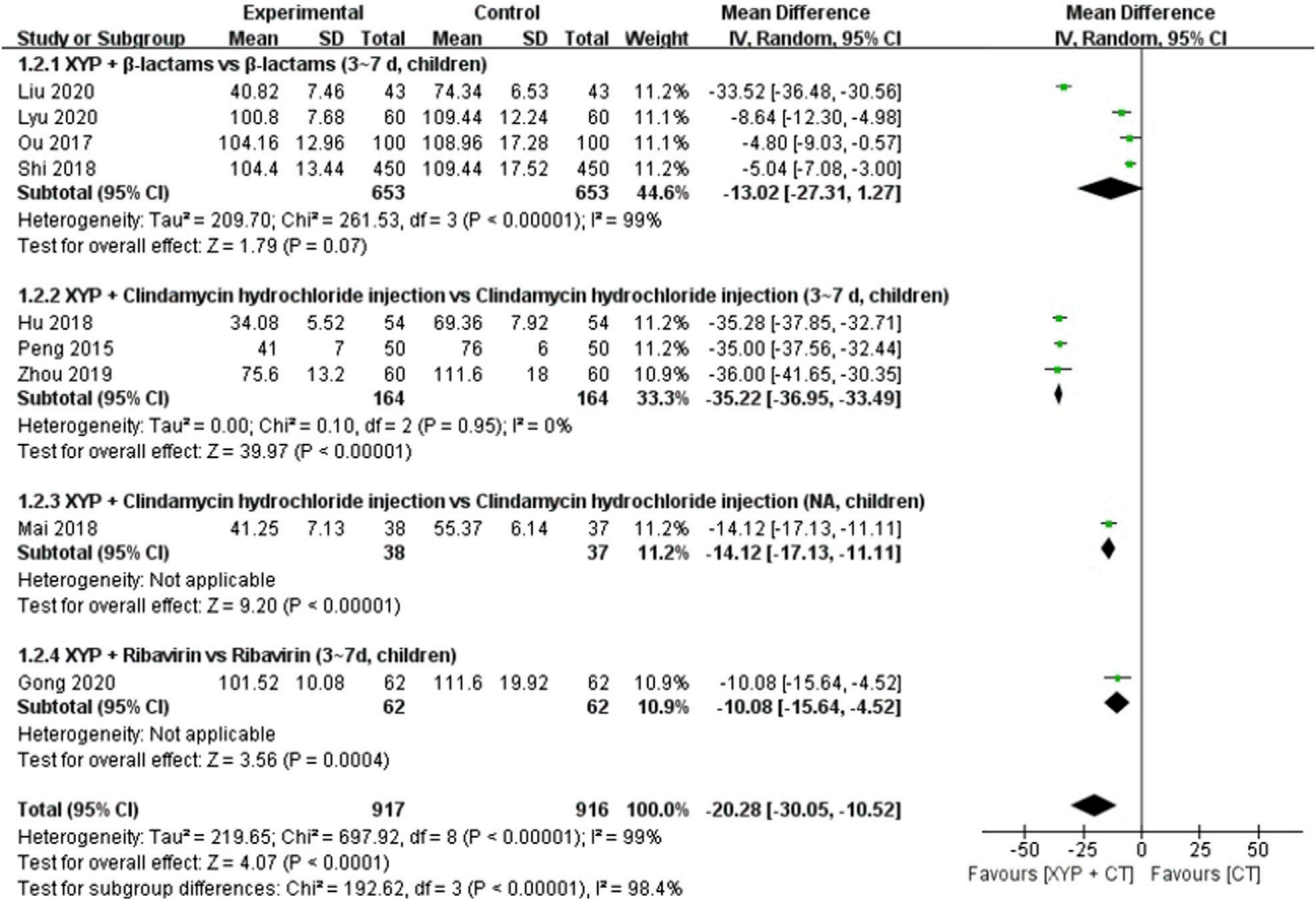
Forest plot of the duration of the disappearance of tonsillar redness and swelling.

Nine studies (Peng et al., 2015; Ou et al., 2017; Hu et al., 2018; Mai, 2018; Shi, 2018; Zhou, 2019; Gong, 2020; Liu, 2020; Lyu, 2020) comprising 1,833 cases reported the duration of the disappearance of tonsillar redness and swelling. The forest plot (Figure 5) demonstrated significant differences between the treatment and control groups (MD = −20.28, 95% CI: −30.05 to −10.52, *p* < 0.0001), with high heterogeneity observed (*p* < 0.00001, I^2^ = 99%). Consequently, a random-effects model was employed to analyze the data.

To address the observed heterogeneity, subgroup analyses were conducted based on the combination medication, duration of treatment, and age of the patients. Specifically, four studies (Ou et al., 2017; Shi, 2018; Liu, 2020; Lyu, 2020) compared the efficacy of XYP plus β-lactams versus β-lactams alone over a treatment duration of 3–7 days in children. However, significant heterogeneity was noted among these studies (*p* < 0.00001, I^2^ = 99%). Utilizing a random-effects model, the analysis revealed that XYP plus β-lactams (3–7 days, children) was superior to β-lactams alone (3–7 days, children) in shortening the duration of disappearance of tonsillar redness and swelling (MD = −13.02, 95% CI: −27.31 to −1.27, *p* < 0.00001; Figure 5). Notably, the confidence interval of one trial (Liu, 2020) did not overlap with that of the other three trials (Ou et al., 2017; Shi, 2018; Lyu, 2020). After reviewing the original study, it was observed that one trial (Liu, 2020) had fewer than 90 cases, which was considered a significant contributor to the heterogeneity. Therefore, this trial was excluded from the analysis, and the remaining three trials (Ou et al., 2017; Shi, 2018; Lyu, 2020) were pooled alone (MD = −5.73, 95% CI: −7.37 to −4.09, *p* < 0.00001; Supplementary Figure S3). Additionally, three studies (Peng et al., 2015; Hu et al., 2018; Zhou, 2019) evaluated the efficacy of XYP plus clindamycin hydrochloride injection compared to clindamycin hydrochloride injection alone over a treatment duration of 3–7 days in children. These studies demonstrated low heterogeneity (*p* = 0.95, I^2^ = 0%). Utilizing a fixed-effects model, the analysis indicated that XYP plus clindamycin hydrochloride injection (3–7 days, children) was more effective than clindamycin hydrochloride injection alone (3–7 days, children) (MD = −35.22, 95% CI: −36.95 to −33.49, *p* < 0.00001; Supplementary Figure S3).

One study (Gong, 2020) demonstrated that XYP plus ribavirin (3–7 days, children) was superior to ribavirin alone (3–7 days, children) in reducing the duration of disappearance of tonsillar redness and swelling (MD = −10.08, 95% CI: −15.64 to −4.52, *p* = 0.0004). Further details are provided in Figure 5.

#### 3.4.2 The time of tonsil purulent discharge

**FIGURE 6:**
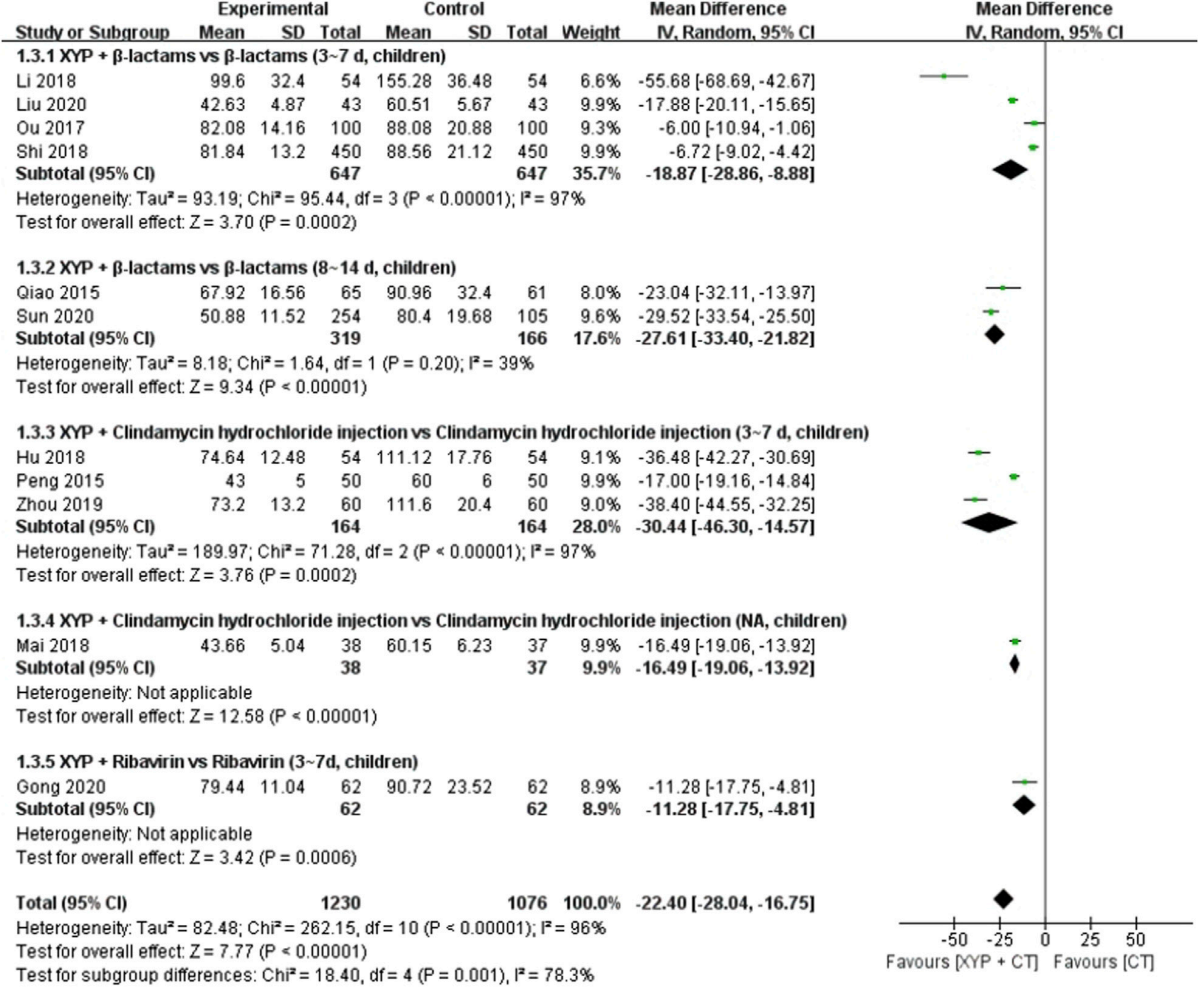
Forest plot of the time of tonsil purulent discharge.

Eleven studies (Peng et al., 2015; Qiao et al., 2015; Li, 2017; Ou et al., 2017; Hu et al., 2018; Mai, 2018; Shi, 2018; Zhou, 2019; Gong, 2020; Liu, 2020; Sun, 2020) comprising 2,306 cases reported the time of tonsil purulent discharge. The forest plot (Figure 6) indicated significant differences between the treatment and control groups (MD = −22.40, 95% CI: −28.04 to −16.75, *p* < 0.00001), with high heterogeneity observed (I^2^ = 96%, *p* < 0.00001). Consequently, a random-effects model was utilized to analyze the data.

To address the observed heterogeneity, subgroup analyses were conducted based on the combination medication, duration of treatment, and age of the patients. Specifically, four studies (Li, 2017; Ou et al., 2017; Shi, 2018; Liu, 2020) compared the efficacy of XYP plus β-lactams versus β-lactams alone over a treatment duration of 3–7 days in children. However, significant heterogeneity was noted among these studies (*p* < 0.00001, I^2^ = 97%). Utilizing a random-effects model, the analysis revealed that XYP plus β-lactams (3–7 days, children) was superior to β-lactams alone (3–7 days, children) in shortening the time of tonsil purulent discharge (MD = −18.87, 95% CI: −28.86 to −8.88, *p* = 0.0002; Figure 6). Notably, the confidence intervals of two trials (Li, 2017; Liu, 2020) did not overlap with those of the other two trials (Ou et al., 2017; Shi, 2018). After reviewing the original studies, it was observed that two trials (Li, 2017; Liu, 2020) had fewer than 90 cases, which likely contributed to the heterogeneity. Therefore, these trials were excluded from the analysis, and the remaining two trials (Ou et al., 2017; Shi, 2018) were pooled alone using a fixed-effects model (MD = −6.59, 95% CI: −8.68 to −4.51, *p* < 0.00001; Supplementary Figure S4). Two studies (Qiao et al., 2015; Sun, 2020) demonstrated that XYP plus β-lactams (8–14 days, children) was superior to β-lactams alone (8–14 days, children) in shortening the time of tonsil purulent discharge, with low heterogeneity observed among studies (*p* = 0.20, I^2^ = 39%). Utilizing a fixed-effects model, the analysis revealed a significant difference favoring XYP plus β-lactams (MD = −28.46, 95% CI: −32.13 to −24.78, *p* < 0.00001; Supplementary Figure S4). Additionally, three studies (Peng et al., 2015; Hu et al., 2018; Zhou, 2019) evaluated the efficacy of XYP plus clindamycin hydrochloride injection compared to clindamycin hydrochloride injection alone over a treatment duration of 3–7 days in children. These studies demonstrated significant heterogeneity among studies (*p* < 0.00001, I^2^ = 97%). After excluding one study (Peng et al., 2015) with fewer than 100 cases, the heterogeneity was significantly reduced (*p* = 0.66, I^2^ = 0%). Utilizing a fixed-effects model, the pooled analysis of the remaining two trials (Hu et al., 2018; Zhou, 2019) indicated that XYP plus clindamycin hydrochloride injection (3–7 days, children) was more effective than clindamycin hydrochloride injection alone (MD = −37.38, 95% CI: −41.60 to −33.17, *p* < 0.00001; Supplementary Figure S4).

**FIGURE 7:**
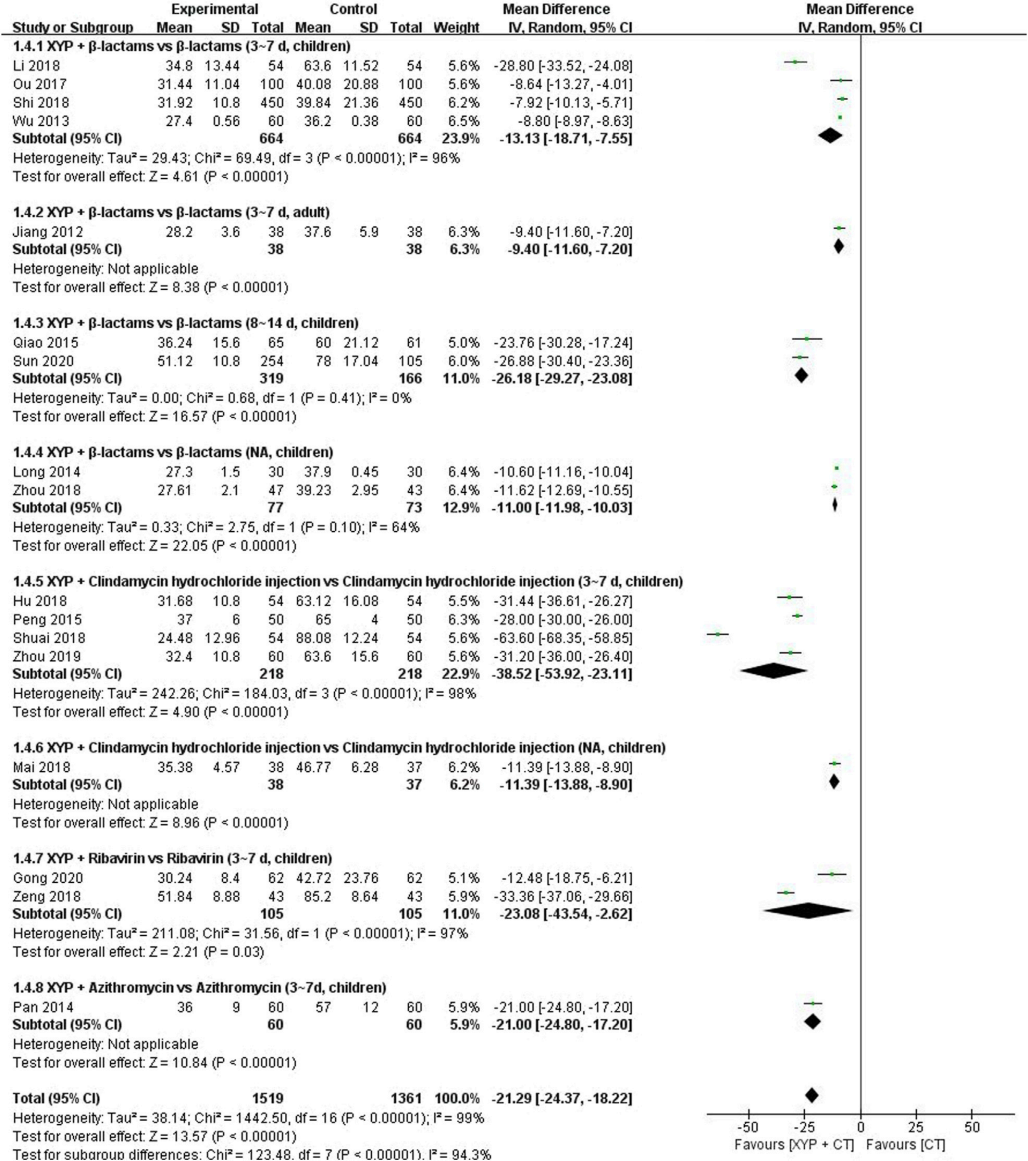
Forest plot of the time of recovering normal temperature.

The findings from the other two studies supported significant differences in the time of tonsil purulent discharge for XYP in combination with clindamycin hydrochloride injection (children) (Mai, 2018) (MD = −16.49, 95% CI: −19.06 to −13.92, *p* < 0.00001) and ribavirin (3–7 days, children) (Gong, 2020) (MD = −11.28, 95% CI: −17.75 to −4.81, *p* = 0.0006) compared to the control group. Further details are given in Figure 7.

#### 3.4.3 The time of recovering normal temperature

Eleven studies (Jiang and Yang, 2012; Wu, 2013; Long and Cai, 2014; Pan et al., 2014; Peng et al., 2015; Qiao et al., 2015; Ou et al., 2017; Hu et al., 2018; Mai, 2018; Shi, 2018; Shuai et al., 2018; Zeng, 2018; Zhou, 2018; Zhou, 2019; Gong, 2020; Sun, 2020) comprising 2,880 cases reported the time of recovering normal temperature. The forest plot (Figure 7) revealed significant differences between the treatment and control groups (MD = −21.29, 95% CI: −24.37 to −18.22, *p* < 0.00001), with high heterogeneity observed (I^2^ = 99%, *p* < 0.00001). Therefore, a random-effects model was utilized to analyze the data.

To address the observed heterogeneity, subgroup analyses were conducted based on the combination medication, duration of treatment, and age of the patients. Specifically, four studies (Wu, 2013; Li, 2017; Ou et al., 2017; Shi, 2018) compared the efficacy of XYP plus β-lactams versus β-lactams alone over a treatment duration of 3–7 days in children. However, significant heterogeneity was noted among these studies (*p* < 0.00001, I^2^ = 96%). Utilizing a random-effects model, the analysis revealed that XYP plus β-lactams (3–7 days, children) was superior to β-lactams alone (3–7 days, children) in shortening the time of recovering normal temperature (MD = −13.13, 95% CI: −18.71 to −7.55, *p* < 0.00001; Figure 7). Notably, the confidence interval of one trial (Li, 2017) did not overlap with those of the other three trials (Wu, 2013; Ou et al., 2017; Shi, 2018). After reviewing the original study, it was observed that one trial (Li, 2017) had fewer than 110 cases, which likely contributed to the heterogeneity. Therefore, this trial was excluded from the analysis, and the remaining three trials (Wu, 2013; Ou et al., 2017; Shi, 2018) were pooled alone using a fixed-effects model (MD = −8.79, 95% CI: −8.97 to −8.62, *p* < 0.00001; Supplementary Figure S5). Additionally, two studies (Qiao et al., 2015; Sun, 2020) compared XYP plus β-lactams (8–14 days, children) with β-lactams alone (8–14 days, children) and demonstrated low heterogeneity among studies (*p* = 0.41, I^2^ = 0%). The findings indicated that XYP plus β-lactams (8–14 days, children) was superior to β-lactams alone (8–14 days, children) in reducing the time of recovering normal temperature (MD = −26.18, 95% CI: −29.27 to −23.08, *p* < 0.00001; Supplementary Figure S5). Moreover, four studies (Peng et al., 2015; Hu et al., 2018; Zhou, 2019; Gong, 2020) investigated the efficacy of XYP plus clindamycin hydrochloride injection (3–7 days, children) compared to clindamycin hydrochloride injection alone (3–7 days, children), revealing significant heterogeneity among studies (*p* < 0.00001, I^2^ = 99%). Despite this heterogeneity, the results favored XYP plus clindamycin hydrochloride injection (3–7 days, children) in reducing the time of recovering normal temperature (MD = −38.52, 95% CI: −53.92 to −23.11, *p* = 0.006; Figure 7). However, one trial (Shuai et al., 2018) lacked data on the specific method of randomization, which likely contributed to the observed heterogeneity (*p* = 0.28, I^2^ = 22%). Consequently, this trial was excluded, and the remaining three trials (Peng et al., 2015; Hu et al., 2018; Zhou, 2019) were pooled using a fixed-effects model (MD = −28.81, 95% CI: −30.55 to −27.07, *p* < 0.00001; Supplementary Figure S5). Furthermore, two studies (Zeng, 2018; Gong, 2020) examined the efficacy of XYP plus ribavirin (3–7 days, children) compared to ribavirin alone (3–7 days, children), revealing significant heterogeneity among studies (*p* < 0.00001, I^2^ = 97%). Despite this heterogeneity, the random-effects model indicated that XYP plus ribavirin (3–7 days, children) was superior to ribavirin alone (3–7 days, children) in reducing the time of recovering normal temperature (MD = −23.08, 95% CI: −43.54 to −2.62, *p* = 0.03; Figure 7).

The other three studies supported that the recovering normal temperature time of XYP and the combination of β-lactams (3–7 days, adult) (Jiang and Yang, 2012) (MD = −9.40, 95% CI: −11.60 to −7.20, *p* < 0.00001), clindamycin hydrochloride injection (children) (Mai, 2018) (MD = −11.39, 95% CI: −13.88 to −8.90, *p* < 0.00001), and azithromycin (3–7 days, children) (Gong, 2020) (MD = −21.00, 95% CI: −24.80 to −17.20, *p* < 0.00001) was significantly different from that of the control group. Details are shown in Figure 7.

#### 3.4.4 Interleukin-6 (U/L)

**FIGURE 8:**
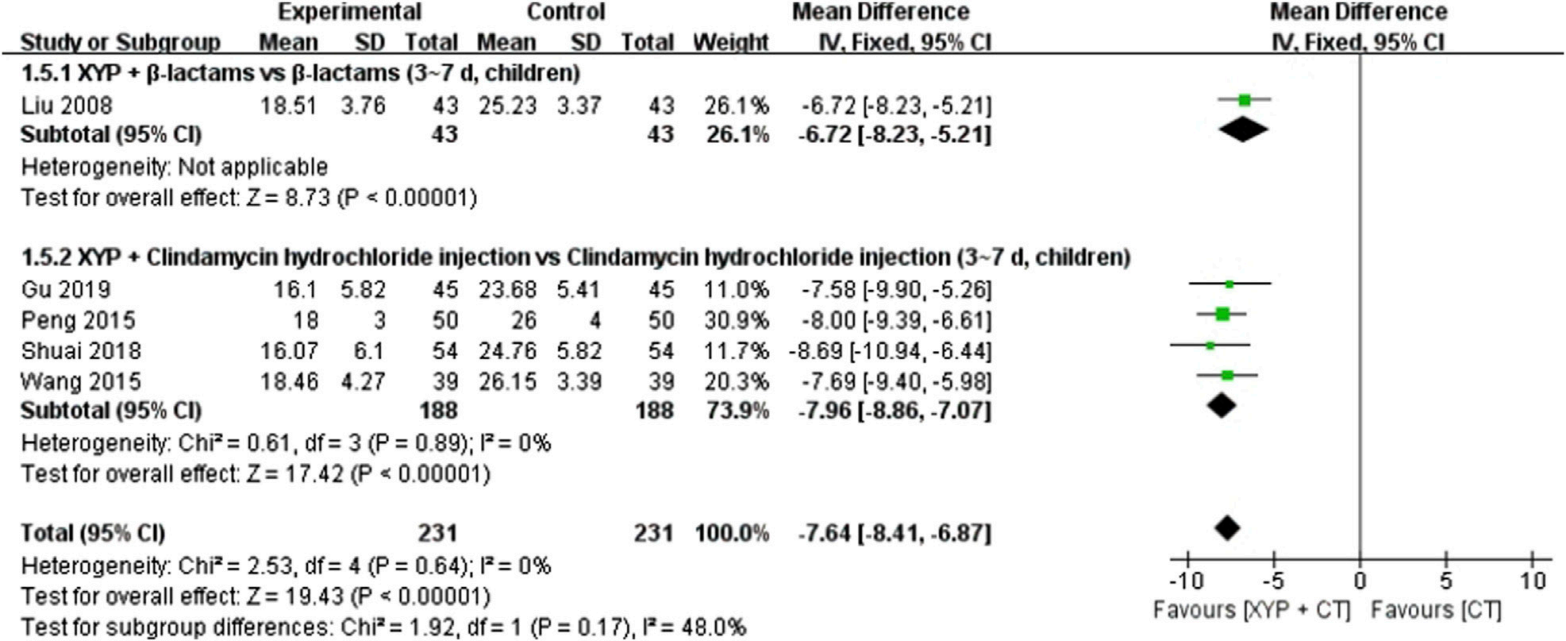
Forest plot of the interleukin-6 (IL-6) level.

Five studies (Liu et al., 2008; Peng et al., 2015; Wang, 2015; Shuai et al., 2018; Gu, 2019) with a total of 445 cases reported on the interleukin-6 (IL-6) indicator. The forest plot (Figure 8) illustrated significant differences between the treatment and control groups (MD = −7.64, 95% CI: −8.41 to −6.87, *p* < 0.00001), and the heterogeneity was low (I^2^ = 0%, *p* = 0.64). Given the low heterogeneity, a fixed-effects model was employed to analyze the data.

To address heterogeneity, subgroup analysis was conducted based on the combination medication, treatment duration, and patient age. Four studies (Peng et al., 2015; Wang, 2015; Shuai et al., 2018; Gu, 2019) examining XYP plus clindamycin hydrochloride injection (3–7 days, children) versus clindamycin hydrochloride injection alone (3–7 days, children) demonstrated low heterogeneity (*p* = 0.89, I^2^ = 0%). These findings revealed that XYP plus clindamycin hydrochloride injection (3–7 days, children) was superior to clindamycin hydrochloride injection alone in reducing IL-6 indicators (MD = −7.96, 95% CI: −8.86 to −7.07, *p* < 0.00001). Additionally, one study (Liu et al., 2008) indicated that XYP plus β-lactams (3–7 days, children) significantly decreased IL-6 indicators compared to β-lactams alone (3–7 days, children) (MD = −6.72, 95% CI: −8.23 to −5.21, *p* < 0.00001). Further details are provided in Figure 8.

#### 3.4.5 Interleukin-8 (U/L)

**FIGURE 9:**
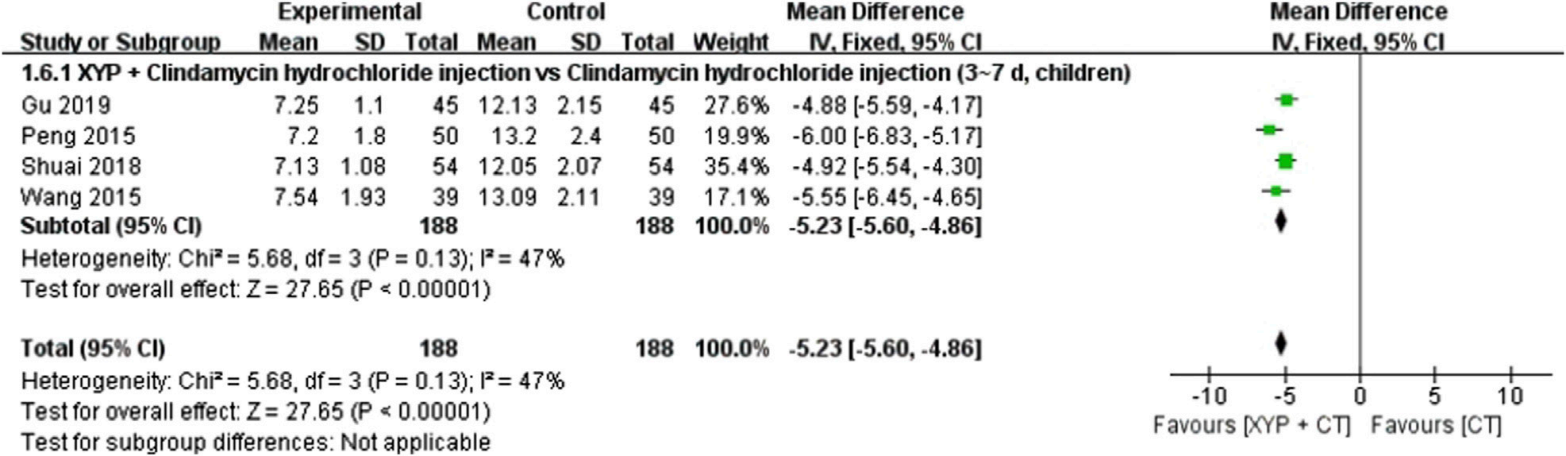
Forest plot of the IL-8 level.

In four studies (Peng et al., 2015; Wang, 2015; Shuai et al., 2018; Gu, 2019) involving 376 cases, interleukin-8 (IL-8) indicators were reported. The forest plot (Figure 9) displayed significant differences between the treatment and control groups (MD = −5.23, 95% CI: −5.60 to −4.86, *p* < 0.00001). With low heterogeneity (I^2^ = 47%, *p* = 0.13), the fixed-effects model was employed for data analysis. The results indicated that XYP plus clindamycin hydrochloride injection (3–7 days, children) outperformed clindamycin hydrochloride injection alone (3–7 days, children) in reducing IL-8 indicators.

#### 3.4.6 Recovery rate

In 29 studies (Dai, 2006; Liu et al., 2008; Dong, 2009; Lu, 2010; Zou, 2010; Ye, 2011; Jiang and Yang, 2012; He, 2013; Wu, 2013; Long and Cai, 2014; Pan et al., 2014; Peng et al., 2015; Qiao et al., 2015; Wang, 2015; Luo, 2016; Li, 2017; Ou et al., 2017; Hu et al., 2018; Mai, 2018; Ren, 2018; Shi, 2018; Shuai et al., 2018; Zhou, 2018; Gu, 2019; Zhou, 2019; Gan et al., 2020; Gong, 2020; Lyu, 2020; Sun, 2020) involving 3,985 cases, the recovery rate of the disease was reported. The forest plot (Figure 11) illustrated significant differences between the treatment and control groups (RR 1.55, 95% CI: 1.37 to 1.77, *p* < 0.00001). Since heterogeneity was high (I^2^ = 73%, *p* < 0.00001), we opted for the random-effects model for data analysis.

**FIGURE 10:**
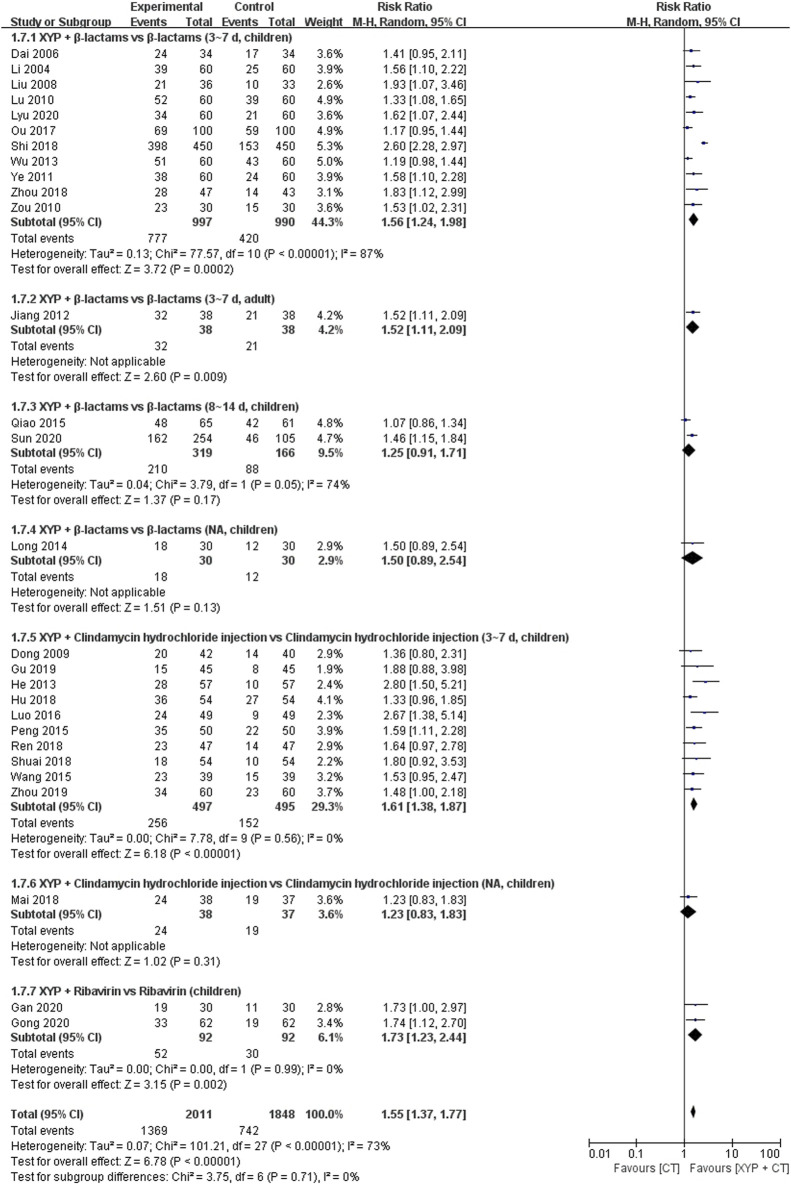
Forest plot of the recovery rate of disease.

In the analysis, despite the heterogeneity not being pronounced, to mitigate potential issues, we conducted subgroup analysis based on the combination medication, treatment duration, and patient age. Eleven studies (Li and Li, 2004; Dai, 2006; Liu et al., 2008; Lu, 2010; Zou, 2010; Ye, 2011; Wu, 2013; Ou et al., 2017; Shi, 2018; Zhou, 2018; Lyu, 2020) comparing XYP plus β-lactams with β-lactams (3–7 days, children) exhibited significant heterogeneity among them (*p* < 0.00001, I^2^ = 87%). The random-effects model demonstrated that XYP plus β-lactams (3–7 days, children) was superior to β-lactams (3–7 days, children) in enhancing the recovery rate (RR = 1.56, 95% CI: 1.24 to 1.98, *p* = 0.0002; Figure 10). Notably, the confidence intervals of three trials (Shi, 2018; Zhou, 2018; Lyu, 2020) did not overlap with those of the other eight trials (Li and Li, 2004; Dai, 2006; Liu et al., 2008; Lu, 2010; Zou, 2010; Ye, 2011; Wu, 2013; Ou et al., 2017; Zhou, 2018). Upon reviewing the original studies, we noted that two trials (Shi, 2018; Lyu, 2020) specified the dosage of medication used, while one trial (Zhou, 2018) employed off-label dosing of antibiotics. We hypothesized that this disparity contributed to the observed heterogeneity. Consequently, we excluded these trials and pooled the data from the remaining eight trials (Li and Li, 2004; Dai, 2006; Liu et al., 2008; Lu, 2010; Zou, 2010; Ye, 2011; Wu, 2013; Ou et al., 2017; Zhou, 2018) using a fixed-effects model (RR = 1.38, 95% CI: 1.25 to 1.53, *p* < 0.00001; Supplementary Figure S6).

In these two studies (Qiao et al., 2015; Sun, 2020), XYP plus β-lactams (8–14 days, children) was compared with β-lactams alone (8–14 days, children) regarding their effectiveness in improving the recovery rate. Although there was considerable heterogeneity among the studies (*p* = 0.05, I^2^ = 74%), the overall analysis did not show a statistically significant difference favoring XYP plus β-lactams (8–14 days, children) over β-lactams (8–14 days, children) in enhancing the recovery rate (RR = 1.25, 95% CI: 0.91 to 1.71, *p* = 0.17; Figure 10).

In these 10 studies (Dong, 2009; He, 2013; Peng et al., 2015; Wang, 2015; Luo, 2016; Hu et al., 2018; Ren, 2018; Shuai et al., 2018; Gu, 2019; Zhou, 2019), XYP plus clindamycin hydrochloride injection (3–7 days, children) was compared with clindamycin hydrochloride injection (3–7 days, children) regarding their effectiveness in improving the recovery rate. The analysis showed low heterogeneity among the studies (*p* = 0.56, I^2^ = 0%). The results indicated that XYP plus clindamycin hydrochloride injection (3–7 days, children) was significantly better than clindamycin hydrochloride injection (3–7 days, children) in enhancing the recovery rate (RR = 1.68, 95% CI: 1.44 to 1.96, *p* < 0.00001; Supplementary Figure S6).

Two studies (Gan et al., 2020; Gong, 2020) compared XYP plus ribavirin (children) with ribavirin alone (children), with low heterogeneity observed among studies (*p* = 0.99, I2 = 0%). The results showed that XYP plus ribavirin (children) was better than ribavirin alone (children) in improving the recovery rate (RR = 1.73, 95% CI: 1.23 to 2.44, *p* = 0.002; Supplementary Figure S6).

Additionally, the other two studies evaluated the recovery rate of XYP in comparison with a combination of β-lactams (3–7 days, adult) (Jiang and Yang, 2012) and clindamycin hydrochloride injection (children). Jiang and Yang (2012) showed a significant difference favoring the combination of β-lactams (RR = 1.52, 95% CI: 1.11 to 2.09, *p* = 0.009), while Mai (2018) did not find a significant difference between XYP and clindamycin hydrochloride injection (RR = 1.23, 95% CI: 0.83 to 1.83, *p* = 0.31). Details are shown in Figure 10.

#### 3.4.7 The recovery rate of the white blood cell count

Five studies (Wu, 2013; Long and Cai, 2014; Pan et al., 2014; Zhou, 2018) involving 466 cases reported the recovery rate of white blood cell count. The forest plot showed significant differences between the treatment and control groups (RR = 1.13, 95% CI: 1.04 to 1.23, *p* = 0.004). There was no heterogeneity (I^2^ = 0%, *p* = 0.79); therefore, we used the fixed-effects model to analyze the data.

**FIGURE 11:**
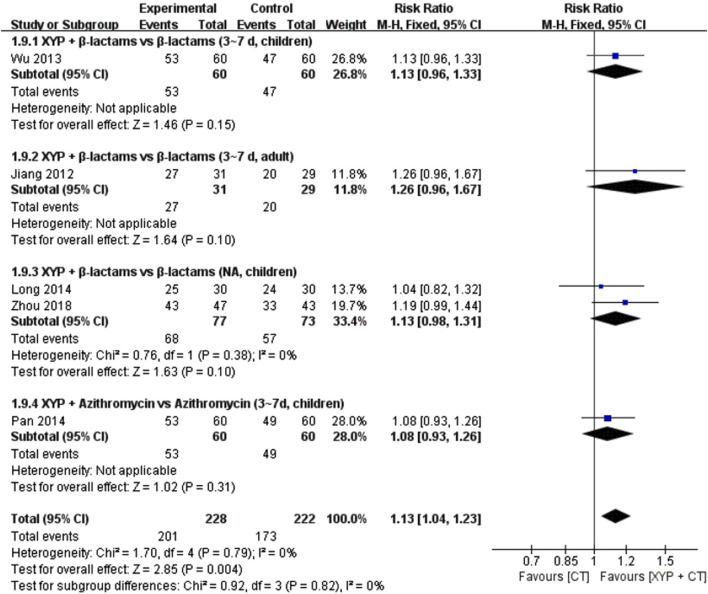
Forest plot of the recovery rate of the white blood cell count.

The other four studies supported that the white blood cell count recovery rate of XYP and the combination of β-lactams (3–7 days, children) (Wu, 2013) (RR = 1.13, 95% CI: 0.96 to 1.33, *p* = 0.15), XYP plus β-lactams (3–7 days, adult) (Jiang and Yang, 2012) (RR = 1.26, 95% CI: 0.96 to 1.67, *p* = 0.10), ribavirin (children) (Gan et al., 2020) (RR = 1.27, 95% CI: 1.01 to 1.61, *p* = 0.05), and azithromycin (3–7 days, children) (Pan et al., 2014) (RR = 1.08, 95% CI: 0.93 to 1.26, *p* = 0.31) was significantly different from the control group. Details are shown in Figure 11.

#### 3.4.8 The disappearance rate of tonsillar redness and swelling

Five studies (Jiang and Yang, 2012; Wu, 2013; Pan et al., 2014; Li, 2017; Zhou, 2018) involving 532 cases reported the disappearance rate of tonsillar redness and swelling. The forest plot showed significant differences between the treatment and control groups (RR = 0.51, 95% CI: 1.14 to 1.38, *p* < 0.00001). There was no heterogeneity (I^2^ = 0%, *p* = 0.66); therefore, we used the fixed-effects model to analyze the data.

Two studies (Wu, 2013; Li, 2017) compared XYP plus β-lactams (3–7 days, children) with β-lactams (3–7 days, children), with low heterogeneity among studies (*p =* 0.94, I^2^ = 0%). The results showed that XYP plus β-lactams (3–7 days, children) was better than β-lactams (3–7 days, children) in improving the disappearance rate of tonsillar redness and swelling (RR = 1.18, 95% CI: 1.04 to 1.34, *p* = 0.010).

**FIGURE 12:**
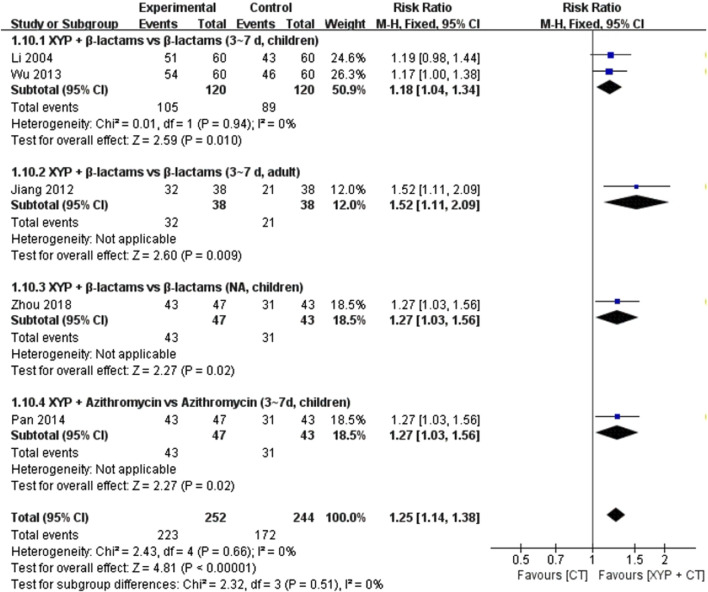
Forest plot of the disappearance rate of tonsillar redness and swelling.

The other two studies supported that the tonsillar redness and swelling disappearance rate of XYP and the combination of β-lactams (3–7 days, adult) (Jiang and Yang, 2012) (RR = 1.52, 95% CI: 1.11 to 2.09, *p* = 0.009) and azithromycin (3–7 days, children) (Pan et al., 2014) (RR = 1.27, 95% CI: 1.03 to 1.56, *p* = 0.02) was significantly different from that in the control group. Details are shown in Figure 12.

### 3.5 Safety outcomes

#### 3.5.1 Incidence of AEs

**FIGURE 13:**
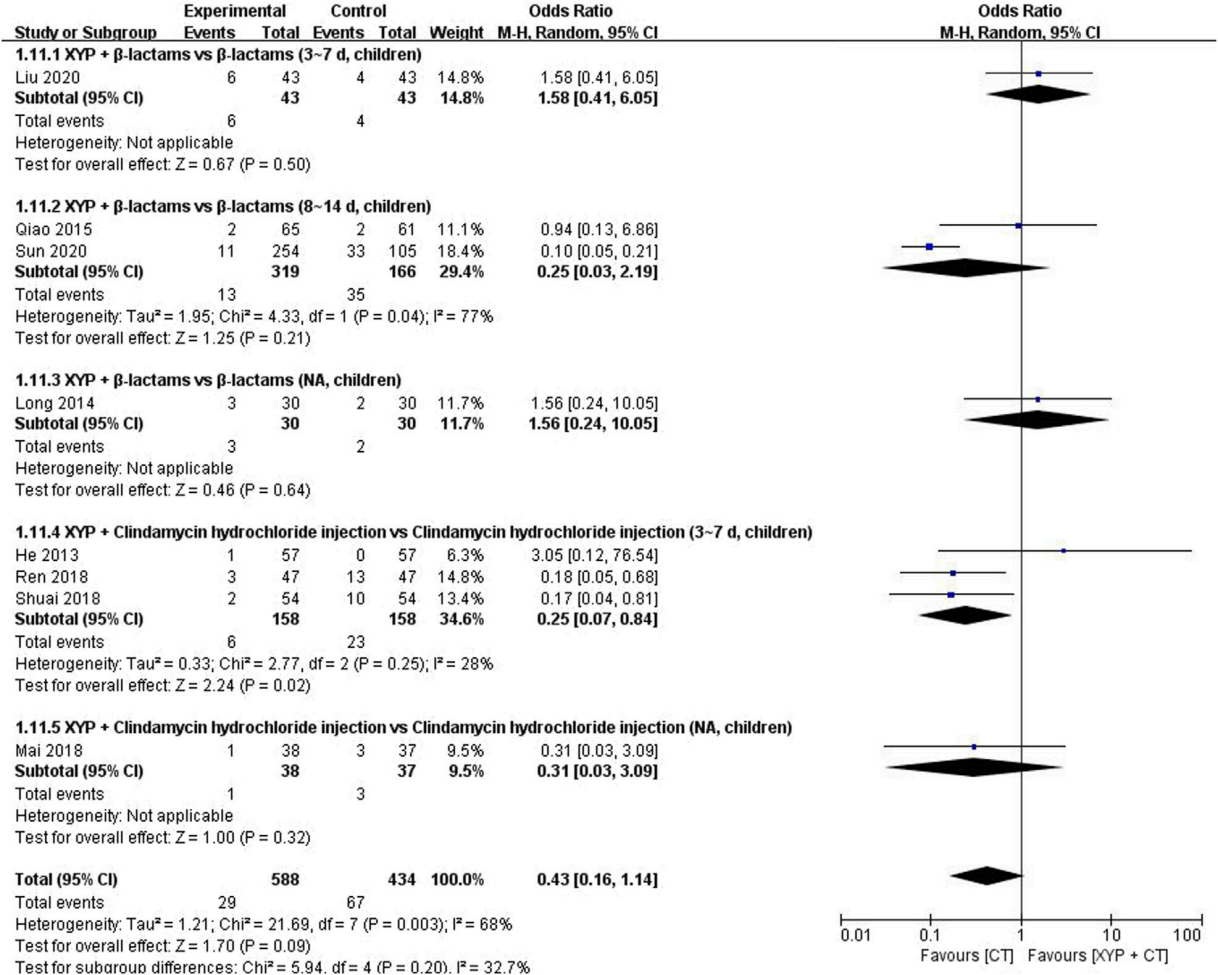
Forest plot of the incidence of adevrse events (AEs).

Eight studies (He, 2013; Qiao et al., 2015; Mai, 2018; Ren, 2018; Shuai et al., 2018; Liu, 2020; Sun, 2020) involving 1,022 cases reported the incidence of AEs. The forest plot (Figure 13) showed significant differences between the treatment and control groups (OR = 0.43, 95% CI: 0.16 to 1.14, *p* = 0.09). The heterogeneity was high (I^2^ = 68%, *p* = 0.003); therefore, we used the random-effects model to analyze the data.

Two studies (Qiao et al., 2015; Sun, 2020) demonstrated that the administration of XYP plus β-lactams (8–14 days) in children exhibited a lower incidence of adverse events than the use of β-lactams alone, with a minimal heterogeneity among the studies (*p* = 0.04, I^2^ = 77%). Specifically, the odds ratio (OR) for reduced adverse events was 0.25 (95% CI: 0.03 to 2.19, *p* = 0.21; Figure 13). Furthermore, an analysis of three studies (He, 2013; Ren, 2018; Shuai et al., 2018) revealed that the combination of XYP and clindamycin hydrochloride injection (3–7 days) in children, as opposed to clindamycin hydrochloride injection alone, yielded a higher disappearance rate of tonsillar redness and swelling, with low heterogeneity observed among the studies (*p* = 0.25, I^2^ = 28%). The fixed-effects model estimated the odds ratio to be 0.24 (95% CI: 0.10 to 0.58, *p* = 0.002; Supplementary Figure S7).

One study (Sun, 2020) indicated that XYP plus β-lactams (3–7 days, children) demonstrated superiority over β-lactams (3–7 days, children) in reducing the incidence of adverse events (RR = 1.50, 95% CI: 0.46 to 4.94, *p* = 0.51). Similarly, another study (Mai, 2018) revealed that XYP plus clindamycin hydrochloride injection (children) outperformed clindamycin hydrochloride injection (children) in shortening the duration of tonsillar redness and swelling disappearance (RR = 0.32, 95% CI: 0.04 to 2.98, *p* = 0.32). Further details are presented in Figure 13.

### 3.6 Subgroup analysis

The subgroup analysis of XYP was conducted based on various treatment durations, age groups, and combination regimens. Subgroup differences were observed in the duration of sore throat (92.1%), the duration of the disappearance of tonsillar redness and swelling (98.4%), the time of tonsil purulent discharge (78.3%), and the time of recovering normal temperature (93.9%).

Regarding the duration of sore throat, heterogeneous results were observed across subgroups. In the comparison of XYP+β-lactams vs. β-lactams (3–7 days, children), six studies exhibited 99% heterogeneity, while in the XYP+β-lactams vs. β-lactams (8–14 days, children) subgroup, two studies showed 93% heterogeneity. Similarly, in the comparison of XYP+clindamycin hydrochloride injection vs. clindamycin hydrochloride injection (3–7 days, children), four studies demonstrated 93% heterogeneity. For the duration of the disappearance of tonsillar redness and swelling, the XYP+β-lactams vs. β-lactams (3–7 days, children) subgroup, comprising four studies, exhibited 99% heterogeneity. Regarding the time of tonsil purulent discharge, both the XYP+β-lactams vs. β-lactams (3–7 days, children) subgroup (four studies, 99% heterogeneity) and the XYP+clindamycin hydrochloride injection vs. clindamycin hydrochloride injection (3–7 days, children) subgroup (three studies, 97% heterogeneity) showed high levels of heterogeneity. Concerning the time of recovering normal temperature, varied heterogeneity was observed across subgroups. In the XYP+β-lactams vs. β-lactams (3–7 days, children) subgroup (four studies, 96% heterogeneity) and the XYP+clindamycin hydrochloride injection vs. clindamycin hydrochloride injection (3–7 days, children) subgroup (four studies, 99% heterogeneity), significant heterogeneity was noted. Additionally, the XYP+β-lactams vs. β-lactams (NA, children) subgroup (two studies, 64% heterogeneity) and the XYP+ribavirin vs. ribavirin (3–7 days, children) subgroup (two studies, 97% heterogeneity) also exhibited notable heterogeneity.

The remaining subgroups had only one study each, making it impossible to analyze within-group heterogeneity. Among the indicators, heterogeneity was insignificant for IL-6 (48%), recovery rate (0%), recovery rate of white blood cell count (0%), recovery rate of antiadoncus (0%), and the incidence of AEs (36.9%). However, to eliminate potential sources of heterogeneity and facilitate further research, subgroup analyses were performed on the aforementioned indicators.

Regarding the recovery rate, 11 studies were included in the XYP+β-lactams vs. β-lactams (3–7 days, children) subgroup, with 87% heterogeneity within the subgroup, while two studies were included in the XYP+β-lactams vs. β-lactams (8–14 days, children) subgroup, with 74% heterogeneity within the subgroup. For the other indicators where data from two or more articles were included, subgroup heterogeneity was <50%.

### 3.7 Evaluation of publication bias

The duration of sore throat was utilized as the outcome measure in 17 studies (Jiang and Yang, 2012; Wu, 2013; Long and Cai, 2014; Pan et al., 2014; Peng et al., 2015; Qiao et al., 2015; Ou et al., 2017; Hu et al., 2018; Mai, 2018; Shi, 2018; Shuai et al., 2018; Zhou, 2018; Zhou, 2019; Gong, 2020; Liu, 2020; Lyu, 2020; Sun, 2020). A funnel chart (Supplementary Figure S8) was generated using the effect size MD as the horizontal axis. However, publication bias was detected through Egger’s regression test (Egger’s test *p* = 0.001; Supplementary Figure S9). The asymmetry of the graph suggested the presence of publication bias, likely attributed to language publication bias since all the included studies were in Chinese.

### 3.8 Sample size estimate

In this study, TSA was employed to ascertain whether the sample size met the minimum required standard. A type I error of 0.05 and a statistical power (power) of 0.8 were set. A relative risk reduction (RRR) of 10% was assumed. The information axis depicted the cumulative sample size. The TSA revealed an estimated sample size of 1,610 patients. Notably, the meta-analysis incorporated 17 studies with 2,892 cases, surpassing the estimated sample size. Consequently, the meta-analysis unveiled statistically significant efficacy disparities, indicating that the sample size was adequate to conclude that XYP effectively improves SAT (Supplementary Figure S10).

### 3.9 GRADE evidence quality evaluation of outcome indicators

GRADE evidence quality evaluation was conducted on six outcomes, encompassing treatment efficacy, duration of fever, duration of cough, duration of nasal congestion, duration of pharyngeal congestion, and incidence of AEs (Table 2).

**TABLE 2:** GRADE evidence grading of XYP combined with CT for suppurative tonsillitis.

Certainty assessment								No. of patients		Effect		Certainty importance	Importance
Outcome	Design	No. of studies	Risk of bias	Inconsistency	Indirectness	Imprecision	Other considerations	Experimental group	Control group	Relative (95% CI)	Absolute (95% CI)		
XYP + CTs vs. CTs													
Duration of sore throat (h)	Randomized trials	17	Serious^a^ ^,^ ^b^ ^,^ ^c^ ^,^ ^d^	Serious^e^	Not serious	Serious^f^	Publication bias strongly suspected^h^	1,525	1,367	—	MD 21.08 lower (24.86 lower to 17.29 lower)	⊕○○○ Very low	Importance
Duration of disappearance of tonsillar redness and swelling (h)	Randomized trials	9	Serious^a^ ^,^ ^b^ ^,^ ^c^	Serious^e^	Not serious	Serious^e^	Publication bias strongly suspected^h^	917	916	—	MD 20.28 lower (30.05 lower to 10.52 lower)	⊕○○○ Very low	Importance
Time of recovering normal temperature (h)	Randomized trials	17	Serious^a^ ^,^ ^b^ ^,^ ^c^ ^,^ ^d^	Serious^e^	Not serious	Serious^f^	Publication bias strongly suspected^h^	1,519	1,361	—	MD 19.48 lower (22.49 lower to 16.47 lower)	⊕○○○ Very low	Importance
Time of tonsil purulent discharge (h)	Randomized trials	11	Serious^a^ ^,^ ^b^ ^,^ ^c^ ^,^ ^d^	Serious^e^	Not serious	Serious^f^	Publication bias strongly suspected^h^	1,230	1,076	—	MD 22.4 lower (28.04 lower to 16.75 lower)	⊕○○○ Very low	Importance
Interleukin-6 (U/L)	Randomized trials	5	Serious^a^ ^,^ ^b^ ^,^ ^c^	Not serious	Not serious	Serious^f^	Publication bias strongly suspected^h^	231	231	—	MD 7.64 lower (8.41 lower to 6.87 lower)	⊕○○○ Very low	Importance
Interleukin-8 (U/L)	Randomized trials	4	Serious^a^ ^,^ ^b^ ^,^ ^c^	Not serious	Not serious	Serious^f^	Publication bias strongly suspected^h^	188	188	—	MD 5.28 lower (5.8 lower to 4.76 lower)	⊕○○○ Very low	Importance
Recovery rate	Randomized trials	28	Serious^a^ ^,^ ^b^ ^,^ ^c^ ^,^ ^d^	Serious^e^	Not serious	Serious^f^	Publication bias strongly suspected^h^	1,369/2,011 (68.1%)	742/1,848 (40.2%)	RR 1.55 (1.37–1.77)	221 more per 1,000 (from 149 more to 309 more)	⊕○○○ Very low	Importance
Recovery rate of white blood cell count	Randomized trials	5	Serious^a^ ^,^ ^b^ ^,^ ^c^ ^,^ ^d^	Not serious	Not serious	Serious^f^	Publication bias strongly suspected^h^	201/228 (88.2%)	173/222 (77.9%)	RR 1.13 (1.04–1.23)	101 more per 1,000 (from 31 more to 179 more)	⊕○○○ Very low	Importance
Disappearance rate of tonsillar redness and swelling	Randomized trials	5	Serious^a^ ^,^ ^b^ ^,^ ^c^ ^,^ ^d^	Not serious	Not serious	Not serious	Publication bias strongly suspected^h^	223/252 (88.5%)	172/244 (70.5%)	RR 1.25 (1.14–1.38)	176 more per 1,000 (from 99 more to 268 more)	⊕⊕○○ Low	Critical
Incidence of AEs	Randomized trials	8	Serious^a^ ^,^ ^b^ ^,^ ^c^ ^,^ ^d^	Serious^e^	Not serious	Serious^f^	Publication bias strongly suspected^h^	29/588 (4.9%)	67/434 (15.4%)	RR 0.31 (0.21–0.45)	107 fewer per 1,000 (from 122 fewer to 85 fewer)	⊕○○○ Very low	Importance
Subgroup analysis													
XYP + β-lactams vs. β-lactams (3∼7 d, children)													
Duration of sore throat (h)	Randomized trials	6	Serious^a^ ^,^ ^b^ ^,^ ^c^ ^,^ ^d^	Serious^e^	Not serious	Serious^f^	Publication bias strongly suspected^h^	760	756	—	MD 13.83 lower (19.35 lower to 8.3 lower)	⊕○○○ Very low	Importance
Duration of the disappearance of tonsillar redness and swelling (h)	Randomized trials	4	Serious^a^ ^,^ ^b^ ^,^ ^c^	Serious^e^	Not serious	Serious^f^	Publication bias strongly suspected^h^	653	653	—	MD 13.02 lower (27.31 lower to 1.27 higher)	⊕○○○ Very low	Importance
Time of tonsil purulent discharge (h)	Randomized trials	4	Serious^a^ ^,^ ^b^ ^,^ ^c^ ^,^ ^d^	Serious^e^	Not serious	Serious^f^	Publication bias strongly suspected^h^	647	647	—	MD 18.87 lower (28.86 lower to 8.88 lower)	⊕○○○ Very low	Importance
Time of recovering normal temperature (h)	Randomized trials	4	Serious^a^ ^,^ ^b^ ^,^ ^c^ ^,^ ^d^	Serious^e^	Not serious	Not serious	Publication bias strongly suspected^h^	664	664	—	MD 13.13 lower (18.71 lower to 7.55 lower)	⊕○○○ Very low	Importance
Recovery rate	Randomized trials	11	Serious^a^ ^,^ ^b^ ^,^ ^c^ ^,^ ^d^	Serious^e^	Not serious	Serious^f^	Publication bias strongly suspected^h^	777/997 (77.9%)	420/990 (42.4%)	RR 1.56 (1.24–1.98)	238 more per 1,000 (from 102 more to 416 more)	⊕○○○ Very low	Importance
XYP + β-lactams vs. β-lactams (8∼14 d, children)													
Duration of sore throat (h)	Randomized trials	2	Serious^a^ ^,^ ^b^ ^,^ ^c^	Serious^e^	Not serious	Not serious	Publication bias strongly suspected^h^	319	166	-	MD 31.44 lower (45.3 lower to 17.59 lower)	⊕○○○ Very low	Importance
Recovery rate	Randomized trials	2	Serious^a^ ^,^ ^b^ ^,^ ^c^	Serious^e^	Not serious	Not serious	Publication bias strongly suspected^h^	210/319 (65.8%)	88/166 (53.0%)	RR 1.25 (0.91–1.71)	133 more per 1,000 (from 48 fewer to 376 more)	⊕○○○ Very low	Importance
Incidence of AEs	Randomized trials	2	Serious^a^ ^,^ ^b^ ^,^ ^c^	Serious^e^	Not serious	Not serious	Publication bias strongly suspected^h^	13/319 (4.1%)	35/166 (21.1%)	RR 0.17 (0.10–0.31)	175 fewer per 1,000 (from 190 fewer to 145 fewer)	⊕○○○ Very low	Importance
XYP + clindamycin hydrochloride injection vs. clindamycin hydrochloride injection (3∼7 days, children)													
Duration of sore throat (h)	Randomized trials	4	Serious^a^ ^,^ ^b^ ^,^ ^c^	Serious^e^	Not serious	Not serious	Publication bias strongly suspected^h^	218	218	—	MD 39.31 lower (46.14 lower to 32.49 lower)	⊕○○○ Very low	Importance
Duration of the disappearance of tonsillar redness and swelling (h)	Randomized trials	3	Serious^a^ ^,^ ^b^ ^,^ ^c^	Serious^e^	Not serious	Not serious	Publication bias strongly suspected^h^	164	164	—	MD 35.22 lower (36.95 lower to 33.49 lower)	⊕○○○ Very low	Importance
Time of tonsil purulent discharge (h)	Randomized trials	3	Serious^a^ ^,^ ^b^ ^,^ ^c^	Serious^e^	Not serious	Not serious	Publication bias strongly suspected^h^	164	164	—	MD 30.44 lower (46.3 lower to 14.57 lower)	⊕○○○ Very low	Importance
Time of recovering normal temperature (h)	Randomized trials	4	Serious^a^ ^,^ ^b^ ^,^ ^c^	Serious^e^	Not serious	Not serious	Publication bias strongly suspected^h^	218	218	—	MD 30.72 lower (52.44 lower to 9.01 lower)	⊕○○○ Very low	Importance
Interleukin-6 (U/L)	Randomized trials	4	Serious^a^ ^,^ ^b^ ^,^ ^c^ ^,^ ^d^	Not serious	Not serious	Serious^f^	Publication bias strongly suspected^h^	188	188	—	MD 7.96 lower (8.86 lower to 7.07 lower)	⊕○○○ Very low	Importance
XYP + β-lactams vs. β-lactams (NA, children)													
Time of recovering normal temperature (h)	Randomized trials	2	Serious^a^ ^,^ ^b^ ^,^ ^c^ ^,^ ^d^	Serious^e^	Not serious	Serious^f^	Publication bias strongly suspected^h^	77	73	—	MD 11 lower (11.98 lower to 10.03 lower)	⊕○○○ Very low	Importance
XYP + ribavirin vs. ribavirin (3∼7 days, children)													
Time of recovering normal temperature (h)	Randomized trials	2	Serious^a^ ^,^ ^b^ ^,^ ^c^	Serious^e^	Not serious	Not serious	Publication bias strongly suspected^h^	105	105	—	MD 23.08 lower (43.54 lower to 2.62 lower)	⊕○○○ Very low	Importance
Sensitivity analysis													
XYP + β-lactams vs. β-lactams (3∼7 days, children)													
Duration of sore throat (h)	Randomized trials	4	Serious^a^ ^,^ ^b^ ^,^ ^c^	Not serious	Not serious	Serious^f^	Publication bias strongly suspected^h^	657	653	—	MD 12.03 lower (12.73 lower to 11.32 lower)	⊕○○○ Very low	Importance
Duration of the disappearance of tonsillar redness and swelling (h)	Randomized trials	3	Serious^a^ ^,^ ^b^ ^,^ ^c^	Not serious	Not serious	Not serious	Publication bias strongly suspected^h^	610	610	—	MD 5.95 lower (8.19 lower to 3.7 lower)	⊕⊕○○ Low	Importance
Time of tonsil purulent discharge (h)	Randomized trials	2	Serious^a^ ^,^ ^b^ ^,^ ^c^	Not serious	Not serious	Not serious	Publication bias strongly suspected^h^	550	550	—	MD 6.59 lower (8.68 lower to 4.51 lower)	⊕⊕○○ Low	Importance
Time of recovering normal temperature (h)	Randomized trials	3	Serious^a^ ^,^ ^b^ ^,^ ^c^	Not serious	Not serious	Not serious	Publication bias strongly suspected^h^	610	610	—	MD 8.79 lower (8.97 lower to 8.62 lower)	⊕⊕○○ Low	Importance
Recovery rate	Randomized trials	9	Serious^a^ ^,^ ^b^ ^,^ ^c^ ^,^ ^d^	Not serious	Not serious	Serious^f^	Publication bias strongly suspected^h^	345/487 (70.8%)	246/480 (51.2%)	RR 1.35 (1.21–1.49)	179 more per 1,000 (from 108 more to 251 more)	⊕○○○ Very low	Importance
Disappearance rate of tonsillar redness and swelling	Randomized trials	2	Serious^a^ ^,^ ^b^ ^,^ ^c^ ^,^ ^d^	Not serious	Not serious	Not serious	Publication bias strongly suspected^h^	105/120 (87.5%)	89/120 (74.2%)	RR 1.18 (1.04–1.34)	133 more per 1,000 (from 30 more to 252 more)	⊕⊕○○ Low	Critical
XYP + β-lactams vs. β-lactams (8∼14 days, children)													
Time of tonsil purulent discharge (h)	Randomized trials	2	Serious^a^ ^,^ ^b^ ^,^ ^c^	Not serious	Not serious	Not serious	Publication bias strongly suspected^h^	319	166	—	MD 27.61 lower (33.4 lower to 21.82 lower)	⊕⊕○○ Low	Importance
Time of recovering normal temperature (h)	Randomized trials	2	Serious^a^ ^,^ ^b^ ^,^ ^c^	Not serious	Not serious	Not serious	Publication bias strongly suspected^h^	319	166	—	MD 26.18 lower (29.27 lower to 23.08 lower)	⊕⊕○○ Low	Importance
XYP + clindamycin hydrochloride injection vs. clindamycin hydrochloride injection (3∼7 days, children)													
Duration of sore throat (h)	Randomized trials	3	Serious^a^ ^,^ ^b^ ^,^ ^c^	Not serious	Not serious	Not serious	Publication bias strongly suspected^h^	164	164	—	MD 33.31 lower (35.95 lower to 30.67 lower)	⊕⊕○○ Low	Importance
Duration of disappearance of tonsillar redness and swelling (h)	Randomized trials	3	Serious^a^ ^,^ ^b^ ^,^ ^c^	Not serious	Not serious	Not serious	Publication bias strongly suspected^h^	164	164	—	MD 35.22 lower (36.95 lower to 33.49 lower)	⊕⊕○○ Low	Importance
Time of tonsil purulent discharge (h)	Randomized trials	2	Serious^a^ ^,^ ^b^ ^,^ ^c^	Not serious	Not serious	Not serious	Publication bias strongly suspected^h^	114	114	—	MD 37.38 lower (41.6 lower to 33.17 lower)	⊕⊕○○ Low	Importance
Time of recovering normal temperature (h)	Randomized trials	2	Serious^a^ ^,^ ^b^ ^,^ ^c^	Not serious	Not serious	Not serious	Publication bias strongly suspected^h^	104	104	—	MD 28.86 lower (31.78 lower to 25.94 lower)	⊕⊕○○ Low	Importance
Interleukin-8 (U/L)	Randomized trials	4	Serious^a^ ^,^ ^b^ ^,^ ^c^	Not serious	Not serious	Serious^f^	Publication bias strongly suspected^h^	188	188	—	MD 5.28 lower (5.8 lower to 4.76 lower)	⊕○○○ Very low	Importance
Recovery rate	Randomized trials	10	Serious^a^ ^,^ ^b^ ^,^ ^c^ ^,^ ^d^	Not serious	Not serious	Serious^f^	Publication bias strongly suspected^h^	256/497 (51.5%)	152/495 (30.7%)	RR 1.61 (1.38–1.87)	187 more per 1,000 (from 117 more to 267 more)	⊕○○○ Very low	Importance
Incidence of AEs	Randomized trials	3	Serious^a^ ^,^ ^b^ ^,^ ^c^ ^,^ ^d^	Not serious	Not serious	Serious^f^	Publication bias strongly suspected^h^	6/158 (3.8%)	23/158 (14.6%)	RR 0.28 (0.12–0.64)	105 fewer per 1,000 (from 128 fewer to 52 fewer)	⊕○○○ Very low	Importance
XYP + ribavirin vs. ribavirin (children)													
Recovery rate	Randomized trials	2	Serious^a^ ^,^ ^b^ ^,^ ^c^	Not serious	Not serious	Not serious	Publication bias strongly suspected^h^	52/92 (56.5%)	30/92 (32.6%)	RR 1.73 (1.23–2.44)	238 more per 1,000 (from 75 more to 470 more)	⊕⊕○○ Low	Importance
XYP + β-lactams vs. β-lactams (NA, children)													
Recovery rate of white blood cell count	Randomized trials	2	Serious^a^ ^,^ ^b^ ^,^ ^c^ ^,^ ^d^	Not serious	Not serious	Serious^f^	Publication bias strongly suspected^h^	68/77 (88.3%)	57/73 (78.1%)	RR 1.13 (0.98–1.31)	102 more per 1,000 (from 16 fewer to 242 more)	⊕○○○ Very low	Importance

CI, confidence interval; MD, mean difference; RR, risk ratio.

^a^ No details of the random protocol were reported.

The implementation of blinding was not reported.

^c^ Lack of allocation concealment.

^d^ Accounting for the patients and the outcome events is not complete.

^e^ The heterogeneity between studies is large (*p* < 0.1, I2 > 55%).

^f^ The sample size included in the study is too small for imprecision (sample size <100).

^g^ Evaluation of the data suggested publication bias, and there may be an equivalent number of “negative” trials not included in this study.

^h^ Quantitative evaluation of the included data indicated publication bias.

## 4 Discussion

Through systematic searching and screening, 32 RCTs were ultimately included in this meta-analysis to evaluate the potential efficacy and safety of XYP combined with CTs compared to CTs alone for treating patients with SAT. The findings indicated that, when compared with CTs alone, the addition of XYP to CTs could reduce the duration of sore throat and tonsillar redness and swelling, shorten the time of tonsil purulent discharge and recovery of normal temperature, and increase the rates of tonsillar redness and swelling disappearance and overall recovery. Furthermore, it led to decreased levels of IL-6 and IL-8 indicators, as well as white blood cell count, without an increase in ADRs. These results suggest that XYP may have a beneficial role in the treatment of SAT, and adjunctive therapy with XYP could potentially reduce the need for antibiotics.

This systematic review represents the inaugural comprehensive evaluation of the safety and efficacy of XYP for SAT. Rigorous procedures were implemented, including independent study selection, data extraction, and risk of bias assessment, conducted by separate reviewers to mitigate errors. Subgroup analysis was employed to address potential heterogeneity among studies, thereby enhancing the result accuracy. Trial sequential analysis was utilized to estimate the sample size and confirm the efficacy of XYP in SAT treatment. Additionally, the GRADE assessment was applied to appraise the quality of evidence across 10 outcomes, encompassing various clinical parameters. Publication bias was explored via a symmetrical funnel chart, while TSA results affirmed the adequacy of the sample size for obtaining robust conclusions regarding XYP effectiveness in SAT treatment.

Each included study was assessed according to the CONSORT Checklist of Items for Reporting Trials of Chinese Herbal Medicine Formulas (Cheng et al., 2017) (Supplementary Material S3). The findings revealed several limitations across the 32 studies: 1) only 16 studies (Dong, 2009; Jiang and Yang, 2012; He, 2013; Peng et al., 2015; Qiao et al., 2015; Wang, 2015; Luo, 2016; Hu et al., 2018; Mai, 2018; Ren, 2018; Zeng, 2018; Zhou, 2018; Gu, 2019; Zhou, 2019; Lyu, 2020; Sun, 2020) included specifically recorded the illustration of the details of the medicine (such as the proprietary product name, lot number, and name and percentage of the added materials); 2) two studies (Li and Li, 2004; Ou et al., 2017) did not pre-specify the outcome measure; 3) none of the studies recorded the specific sample size determination method, the type of randomization method, the mechanism and method used to implement the random allocation sequence, or the specific blinding protocol; 4) six studies (Li and Li, 2004; Dai, 2006; Liu et al., 2008; Zou, 2010; Ye, 2011; Ou et al., 2017) did not record specific statistical analysis methods; 5) subgroup and adjusted analyses were not performed; 6) all studies were not shown for each primary and secondary outcome, results for each group, and the estimated effect size and its precision (such as the 95% confidence interval); 7) all studies were not subjected to subgroup and adjusted analyses; 8) there were no trial limitations, addressing sources of potential bias and imprecision in the discussion of the study; 9) none of the studies were registered clinical trials; and 10) only three studies (Peng et al., 2015; Qiao et al., 2015; Ou et al., 2017) were funded.

This study had the following limitations: 1) there may be potential differences in drug dosage and comorbidities; 2) limited by the sizes of the included studies, we were only able to perform subgroup analysis based on the interventions; 3) all studies did not mention the blinding, allocation concealment methods, study protocol, and sample size estimation in detail; 4) sample size predictions were made for the primary outcome measure only and not for all outcomes; 5) studies that were registered on clinical trial websites but whose results had not been published in journal were not searched; 6) AEs were described in 12 articles, but there were few data, and there was not a unified standard; 7) the source of publication bias in our analysis was language publication bias because the studies included in the analysis were all in Chinese; 8) the outcomes included in more than five studies were evaluated and showed very low-quality evidence. The number of studies included for outcomes evaluated for low-quality evidence was less than 4. Thus, the credibility of the evidence was low; 9) currently, there is still a lack of uniform clinical norms for the outcome indicators of SAT; most of the indicators were qualitative indicators, such as symptom improvement, and a few were objective quantitative indicators; 10) none of the included studies mentioned that a pharyngeal swab rapid test or culture was examined in the included patients. Therefore, future research needs to focus on measurable indicators; and 11) all studies were not reported according to the CONSORT–Chinese Herbal Medicine Formulas (CONSORT-CHM) checklist.

Based on the findings of this study, the utilization of XYP combined with CTs (such as β-lactams, clindamycin hydrochloride injection, or ribavirin) presents a promising treatment approach for SAT, particularly with clindamycin. This combination therapy demonstrates significant efficacy in reducing the duration of sore throat, disappearance of tonsillar redness and swelling, time of tonsil purulent discharge, time of recovering normal temperature, and lowering IL-6/IL-8 indicators. Among the various combinations assessed, XYP combined with clindamycin hydrochloride injection exhibited the most substantial efficacy in reducing IL-6 levels and shortening the duration of sore throat, tonsillar redness and swelling disappearance, tonsil purulent discharge, and temperature recovery, followed by β-lactams. Furthermore, β-lactams combined with XYP proved more effective than azithromycin in enhancing the recovery rate of the white blood cell count. Additionally, the combination of ribavirin and XYP demonstrated superior efficacy in enhancing the recovery rate compared to clindamycin hydrochloride injection. For patients exhibiting severe symptoms such as sore throat, fever, tonsillar swelling, and copious purulent secretions, and in the absence of drug contraindications, the combination of clindamycin hydrochloride injection and XYP is recommended. Conversely, for patients with mild symptoms of sore throat and fever but high white blood cell counts, β-lactams and XYP are the recommended combination. Regarding safety, the addition of XYP alongside clindamycin hydrochloride injection for SAT treatment is suggested to enhance clinical efficacy while minimizing AEs. Although safety data for the combination of XYP and β-lactams were available in this study, and no significant difference in AE incidence was observed between the combination of XYP and β-lactams versus β-lactams alone, the number of AEs associated with the combination of XYP and β-lactams was higher. Therefore, close monitoring for adverse reactions is advised when using XYP combined with β-lactams/ribavirin/azithromycin for SAT treatment. Further clinical data are warranted to substantiate the safety profile of XYP combined with β-lactams/ribavirin/azithromycin in future studies.

TCM *A. paniculata*, a key component of XYP, is known for its efficacy in clearing heat and detoxifying, cooling blood, and reducing swelling. It has been traditionally used to treat various conditions such as cold and fever, sore throat, mouth and tongue sores, cough, diarrhea, dysentery, and inflammatory conditions like carbuncles, sores, and snake bites. Further research into its activity and mechanisms has revealed promising antiviral properties. Fan et al. demonstrated that XYP, a potential antiviral candidate for dengue virus (DENV) infection, exhibited inhibitory effects on DENV replication. XYP not only inactivated DENV2 but also inhibited its entry into cells. Moreover, it significantly reduced viral replication in a dose-dependent manner, thereby lowering viral load in organs and sera and enhancing survival rates in experimental mice. Notably, the antiviral activity of XYP surpassed that of ribavirin injection, especially in the early and late stages of infection. Regarding safety, Yu et al. revealed that XYP administration at varying doses had no significant adverse effects on the central nervous, cardiovascular, or respiratory systems of experimental animals. Additionally, Liu et al. demonstrated that XYP, either alone or in combination with cefazolin, promoted the recovery of cytophagocytosis in mice infected with *Staphylococcus aureus*. This enhancement in phagocytic function led to improved anti-infection capabilities and antibacterial effects. Xiong et al. underscored the potential of XYP combined with cefazolin in enhancing therapeutic outcomes by promoting neutrophil apoptosis and reducing inflammation in mice. This was evidenced by a decrease in IL-6 levels and an increase in IL-10 levels in plasma, indicating a modulation of the immune response. Given these findings, future studies could explore the potential synergistic effects of XYP combined with clindamycin hydrochloride injection, assessing both efficacy and toxicity. Such investigations may shed light on the therapeutic benefits of this combination in treating SAT while minimizing adverse effects.

Therefore, this study presents the following recommendations: 1) reference to the items in the Cochrane Collaboration Network risk of bias tool should be required for future RCT protocol development. The sample size needs to be increased. Trials should be registered prior to commencement; 2) future research should focus on measurable indicators; 3) more clinical research studies are needed to apply XYP to adults; 4) high-quality, large, prospective, and multicenter studies should be performed; 5) future research studies should be rigorously developed with reference to the checklist in the CONSORT-CHM; and 6) reporting of all AEs regardless of the causality to the investigational product. The quality of drugs is also a very important factor. If the quality of the injection itself is defective, it will lead to infusion reactions in patients. Therefore, the quality control of drugs is very important. In clinical practice, modern detection methods should be used to identify the specific causes of AEs, and infusion reactions should be avoided as adverse drug reactions. Drug quality-related issues are closely related to the efficacy and safety of drugs, which should be paid attention to in the future.

## 5 Conclusion

The present meta-analysis sheds light on the potential efficacy and safety of XYP combined with CTs (β-lactams/clindamycin hydrochloride injection/ribavirin) in the treatment of pediatric patients with SAT. However, only one study has investigated the efficacy and safety of XYP in treating adults with SAT. While the findings suggest that XYP combined with CT therapy may be beneficial for children with SAT, the lack of strong evidence, particularly due to outcomes evaluated as low-quality evidence, underscores the need for further investigation. High-quality, well-designed, multicenter RCTs are essential to confirm these findings in the future. Moreover, future research endeavors should prioritize adopting rigorous experimental designs and enhancing the methodological quality and objectivity of outcome measures. RCTs should adhere strictly to the CONSORT-CHM checklist to ensure rigor and transparency in reporting.

## Data Availability

The datasets presented in this study can be found in online repositories. The names of the repository/repositories and accession number(s) can be found in the article/Supplementary Material.
