# Supplementary material for: Potential efficacy and safety of Xiyanping injection as adjuvant therapy in treatment of suppurative acute tonsillitis: a meta-analysis, trial sequential analysis, and certainty of evidence
Source: Front Pharmacol. 2024 Jun 12;15:1327856. doi: 10.3389/fphar.2024.1327856 (PMC11199392; doi:10.3389/fphar.2024.1327856)
Supplement: Supplementary file 1 [file DataSheet2.PDF]

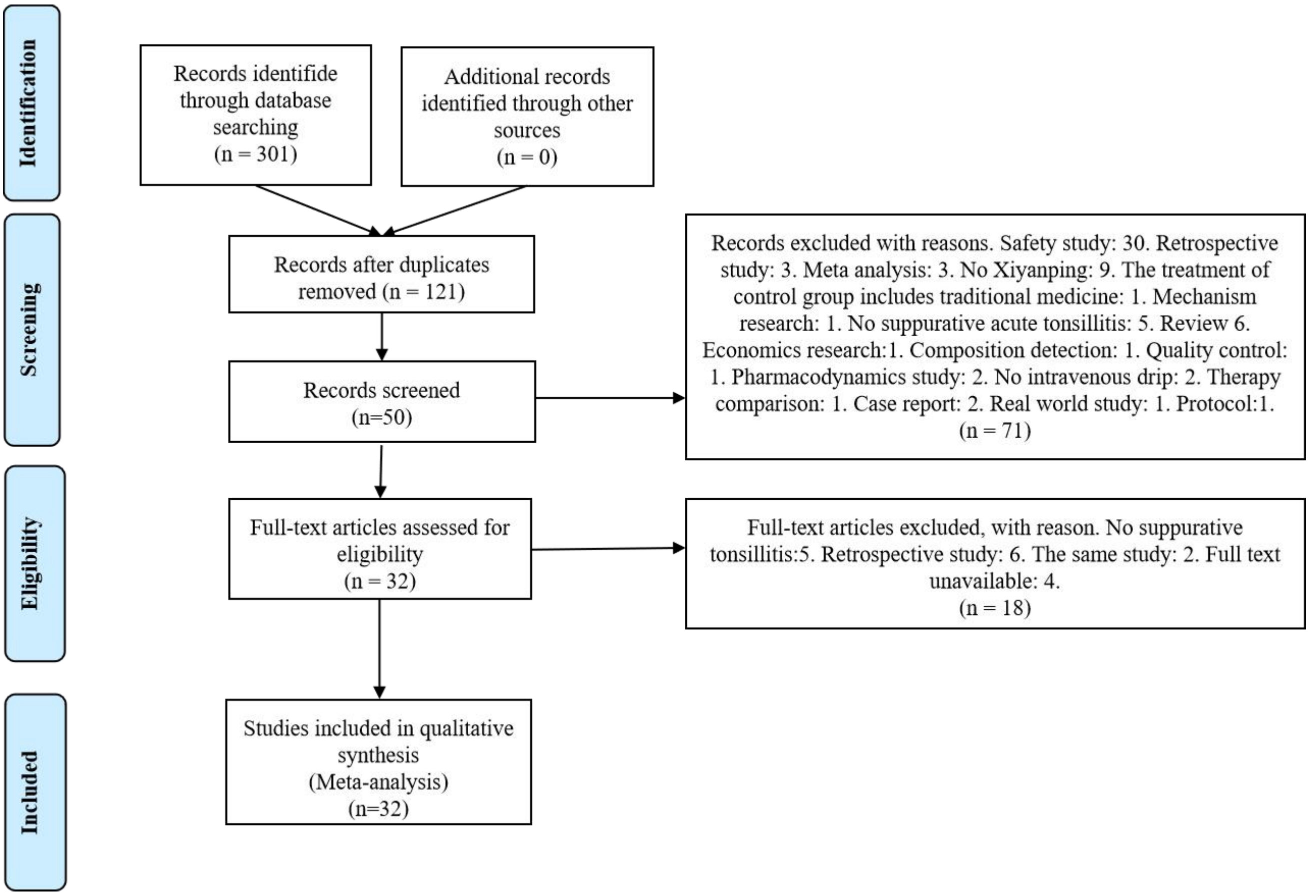

Figure 1. Flow chart of including and excluding studies.

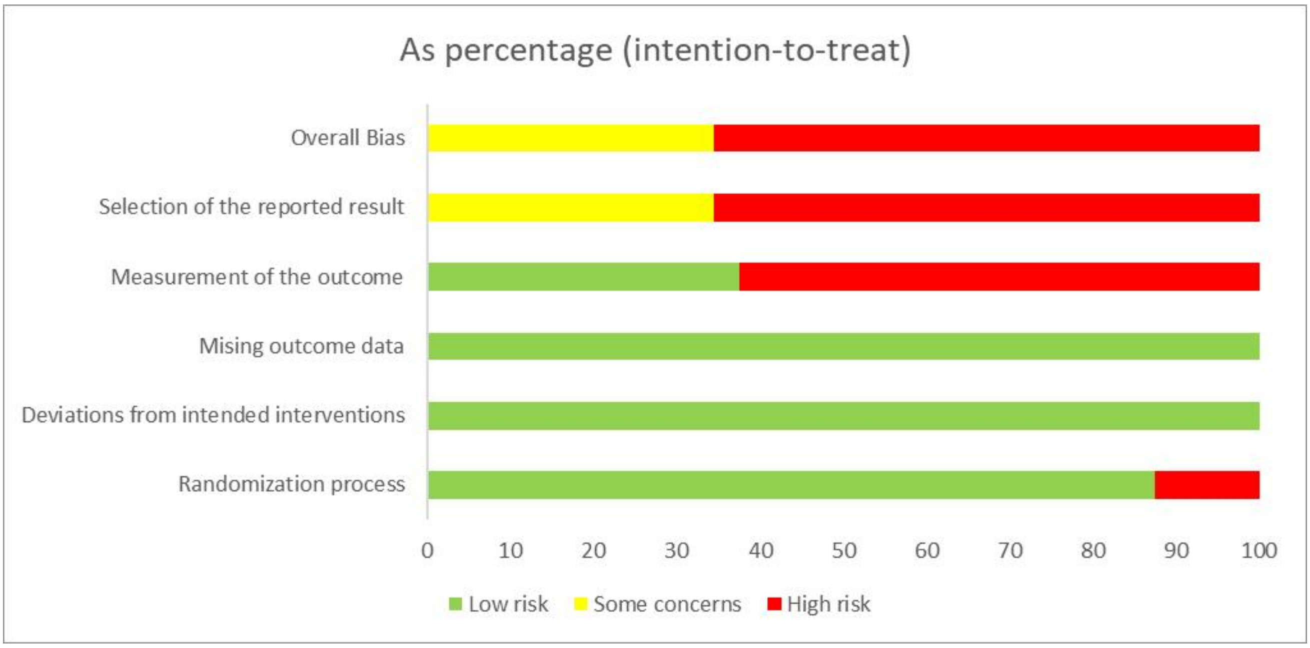

Figure 2. The risk of bias among included studies.

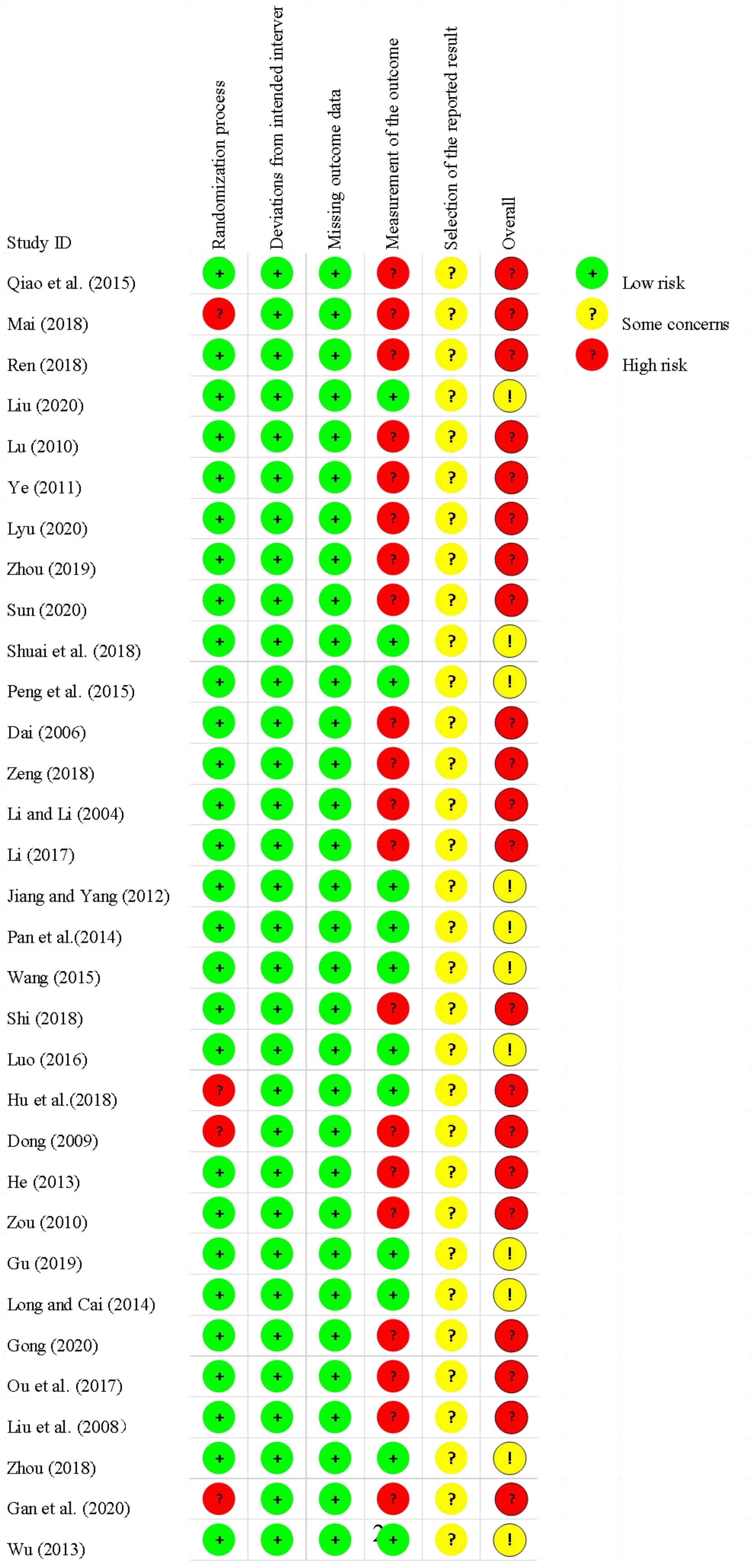

Study ID
Randomization process
Deviations from intended interver
Missing outcome data
Measurement of the outcome
Selection of the reported result
Overall
Qiao et al. (2015)
Mai (2018)
Ren (2018)
Liu (2020)
Lu (2010)
Ye (2011)
Lyu (2020)
Zhou (2019)
Sun (2020)
Shuai et al. (2018)
Peng et al. (2015)
Dai (2006)
Zeng (2018)
Li and Li (2004)
Li (2017)
Jiang and Yang (2012)
Pan et al.(2014)
Wang (2015)
Shi (2018)
Luo (2016)
Hu et al.(2018)
Dong (2009)
He (2013)
Zou (2010)
Gu (2019)
Long and Cai (2014)
Gong (2020)
Ou et al. (2017)
Liu et al. (2008)
Zhou (2018)
Gan et al. (2020)
Wu (2013)
Low risk
Some concerns
High risk

Figure 3. Assessment of risk of bias in the 32 trials.

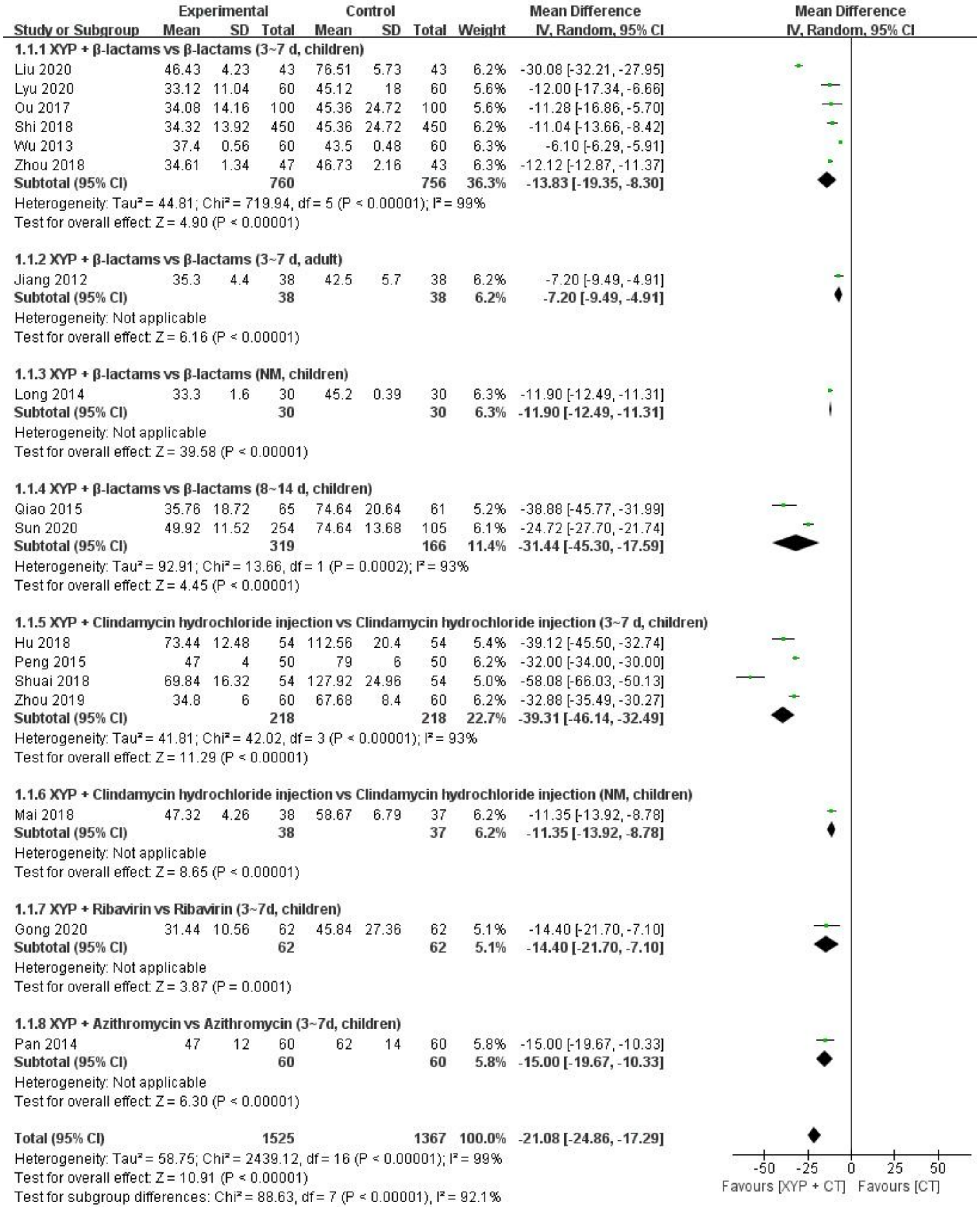

Figure 4. Forest plot of duration of sore throat.

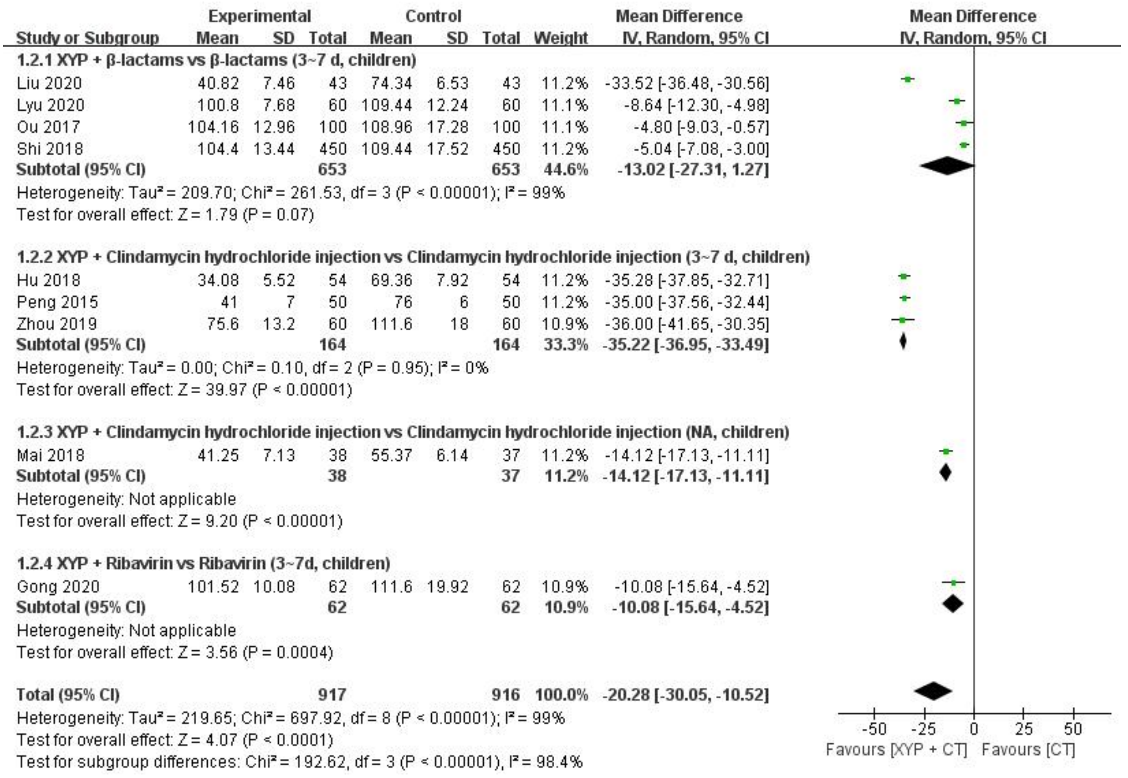

Figure 5. Forest plot of the duration of disappearance of tonsillar redness and swelling.

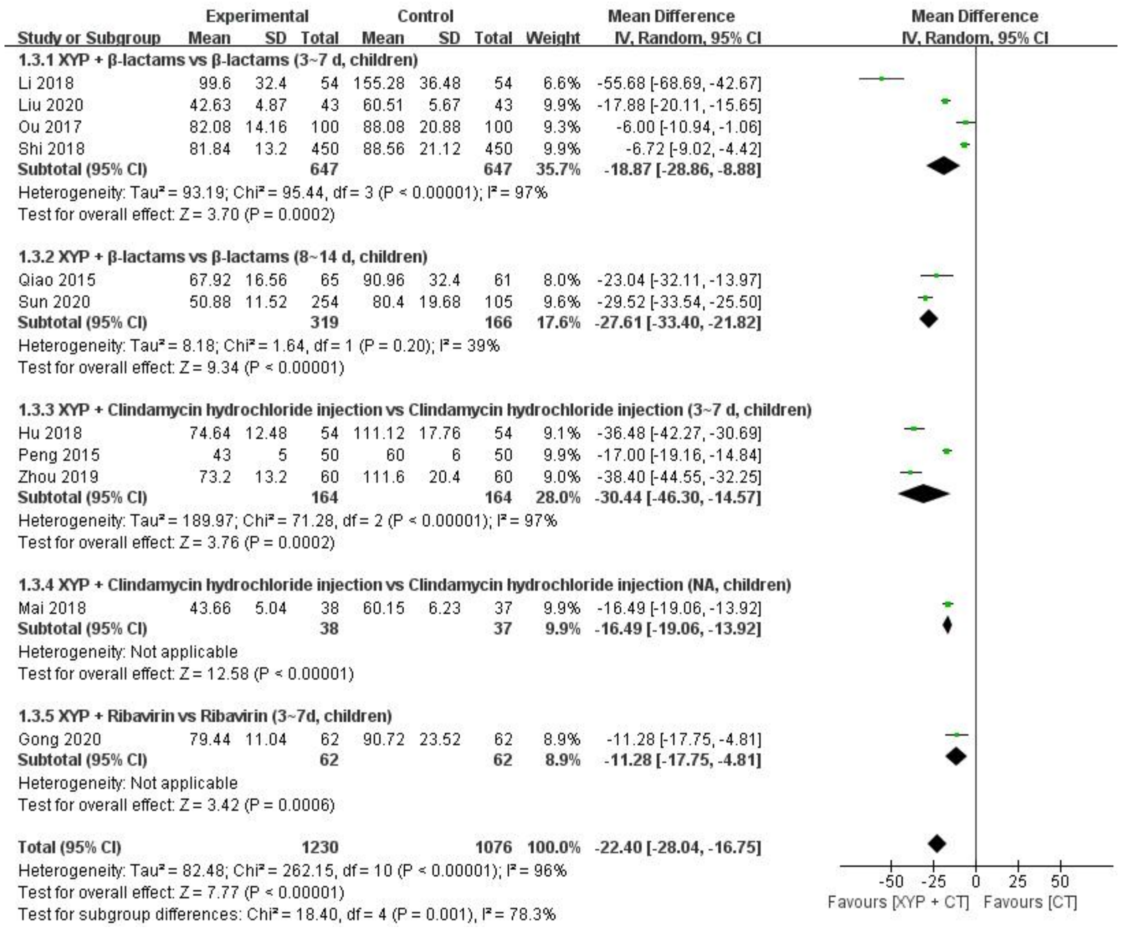

Figure 6. Forest plot of the time of tonsil purulent discharge.

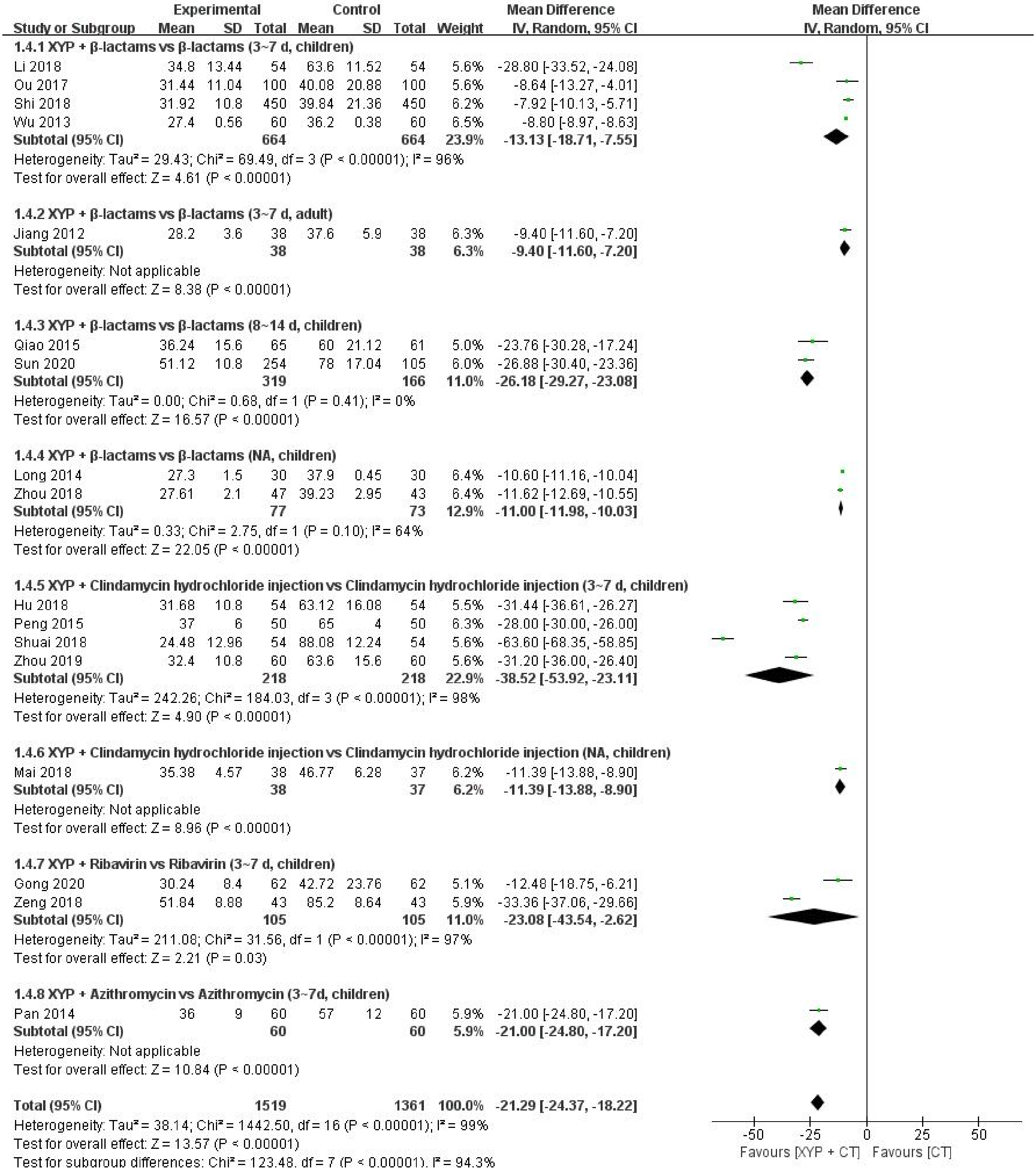

Figure 7. Forest plot of the time of recovering normal temperature.

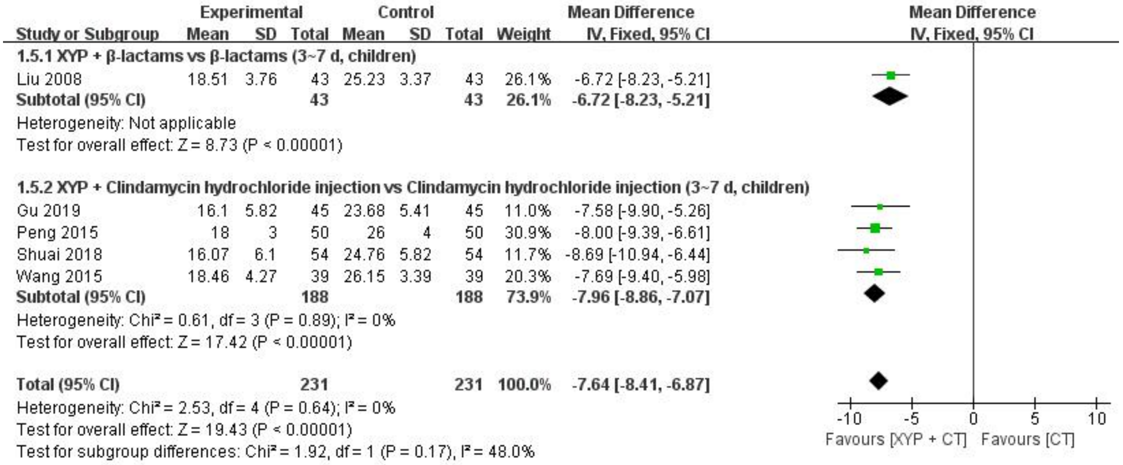

Figure 8. Forest plot of IL-6 level.

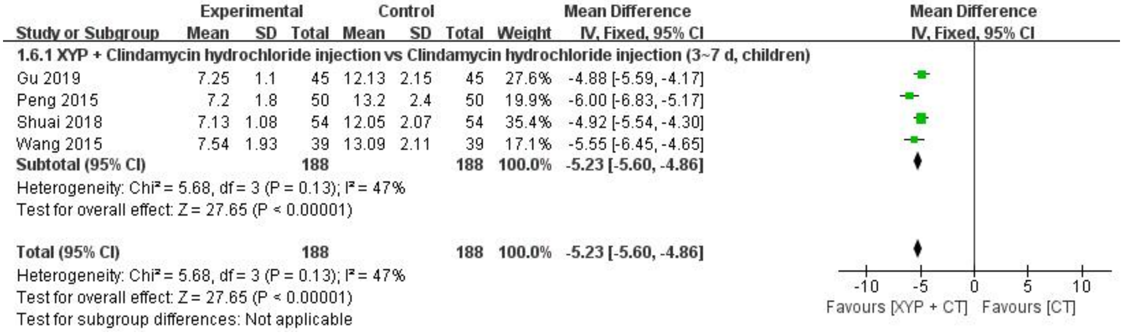

Figure 9. Forest plot of IL-8 level.

Study or Subgroup	Experimental Events	Experimental Total	Control Events	Control Total	Weight	Risk Ratio M-H, Random, 95% CI
**1.7.1 XYP + β-lactams vs β-lactams (3~7 d, children)**						
Dai 2006	24	34	17	34	3.6%	1.41 [0.95, 2.11]
Li 2004	39	60	25	60	3.9%	1.56 [1.10, 2.22]
Liu 2008	21	36	10	33	2.6%	1.93 [1.07, 3.46]
Lu 2010	52	60	39	60	4.9%	1.33 [1.08, 1.65]
Lyu 2020	34	60	21	60	3.6%	1.62 [1.07, 2.44]
Ou 2017	69	100	59	100	4.9%	1.17 [0.95, 1.44]
Shi 2018	398	450	153	450	5.3%	2.60 [2.28, 2.97]
Wu 2013	51	60	43	60	5.0%	1.19 [0.98, 1.44]
Ye 2011	38	60	24	60	3.9%	1.58 [1.10, 2.28]
Zhou 2018	28	47	14	43	3.1%	1.83 [1.12, 2.99]
Zou 2010	23	30	15	30	3.6%	1.53 [1.02, 2.31]
**Subtotal (95% CI)**		**997**		**990**	**44.3%**	**1.56 [1.24, 1.98]**
Total events	777		420			
Heterogeneity: Tau² = 0.13; Chi² = 77.57, df = 10 (P < 0.00001); I² = 87%						
Test for overall effect: Z = 3.72 (P = 0.0002)						
**1.7.2 XYP + β-lactams vs β-lactams (3~7 d, adult)**						
Jiang 2012	32	38	21	38	4.2%	1.52 [1.11, 2.09]
**Subtotal (95% CI)**		**38**		**38**	**4.2%**	**1.52 [1.11, 2.09]**
Total events	32		21			
Heterogeneity: Not applicable						
Test for overall effect: Z = 2.60 (P = 0.009)						
**1.7.3 XYP + β-lactams vs β-lactams (8~14 d, children)**						
Qiao 2015	48	65	42	61	4.8%	1.07 [0.86, 1.34]
Sun 2020	162	254	46	105	4.7%	1.46 [1.15, 1.84]
**Subtotal (95% CI)**		**319**		**166**	**9.5%**	**1.25 [0.91, 1.71]**
Total events	210		88			
Heterogeneity: Tau² = 0.04; Chi² = 3.79, df = 1 (P = 0.05); I² = 74%						
Test for overall effect: Z = 1.37 (P = 0.17)						
**1.7.4 XYP + β-lactams vs β-lactams (NA, children)**						
Long 2014	18	30	12	30	2.9%	1.50 [0.89, 2.54]
**Subtotal (95% CI)**		**30**		**30**	**2.9%**	**1.50 [0.89, 2.54]**
Total events	18		12			
Heterogeneity: Not applicable						
Test for overall effect: Z = 1.51 (P = 0.13)						
**1.7.5 XYP + Clindamycin hydrochloride injection vs Clindamycin hydrochloride injection (3~7 d, children)**						
Dong 2009	20	42	14	40	2.9%	1.36 [0.80, 2.31]
Gu 2019	15	45	8	45	1.9%	1.88 [0.88, 3.98]
He 2013	28	57	10	57	2.4%	2.80 [1.50, 5.21]
Hu 2018	36	54	27	54	4.1%	1.33 [0.96, 1.85]
Luo 2016	24	49	9	49	2.3%	2.67 [1.38, 5.14]
Peng 2015	35	50	22	50	3.9%	1.59 [1.11, 2.28]
Ren 2018	23	47	14	47	2.9%	1.64 [0.97, 2.78]
Shuai 2018	18	54	10	54	2.2%	1.80 [0.92, 3.53]
Wang 2015	23	39	15	39	3.2%	1.53 [0.95, 2.47]
Zhou 2019	34	60	23	60	3.7%	1.48 [1.00, 2.18]
**Subtotal (95% CI)**		**497**		**495**	**29.3%**	**1.61 [1.38, 1.87]**
Total events	256		152			
Heterogeneity: Tau² = 0.00; Chi² = 7.78, df = 9 (P = 0.56); I² = 0%						
Test for overall effect: Z = 6.18 (P < 0.00001)						
**1.7.6 XYP + Clindamycin hydrochloride injection vs Clindamycin hydrochloride injection (NA, children)**						
Mai 2018	24	38	19	37	3.6%	1.23 [0.83, 1.83]
**Subtotal (95% CI)**		**38**		**37**	**3.6%**	**1.23 [0.83, 1.83]**
Total events	24		19			
Heterogeneity: Not applicable						
Test for overall effect: Z = 1.02 (P = 0.31)						
**1.7.7 XYP + Ribavirin vs Ribavirin (children)**						
Gan 2020	19	30	11	30	2.8%	1.73 [1.00, 2.97]
Gong 2020	33	62	19	62	3.4%	1.74 [1.12, 2.70]
**Subtotal (95% CI)**		**92**		**92**	**6.1%**	**1.73 [1.23, 2.44]**
Total events	52		30			
Heterogeneity: Tau² = 0.00; Chi² = 0.00, df = 1 (P = 0.99); I² = 0%						
Test for overall effect: Z = 3.15 (P = 0.002)						
**Total (95% CI)**		**2011**		**1848**	**100.0%**	**1.55 [1.37, 1.77]**
Total events	1369		742			
Heterogeneity: Tau² = 0.07; Chi² = 101.21, df = 27 (P < 0.00001); I² = 73%						
Test for overall effect: Z = 6.78 (P < 0.00001)						
Test for subgroup differences: Chi² = 3.75, df = 6 (P = 0.71), I² = 0%						

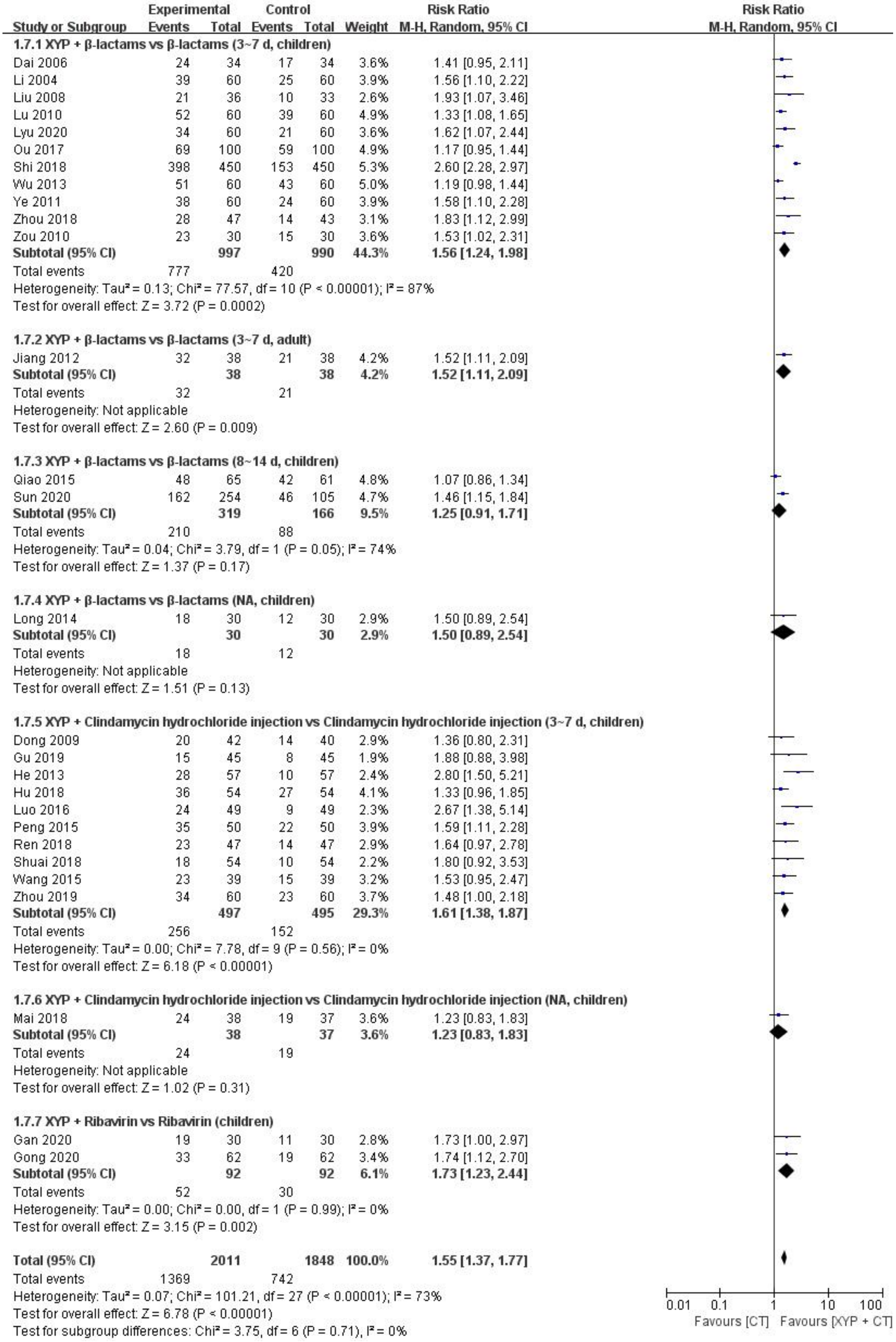

Figure 10. Forest plot of recovery rate of disease.

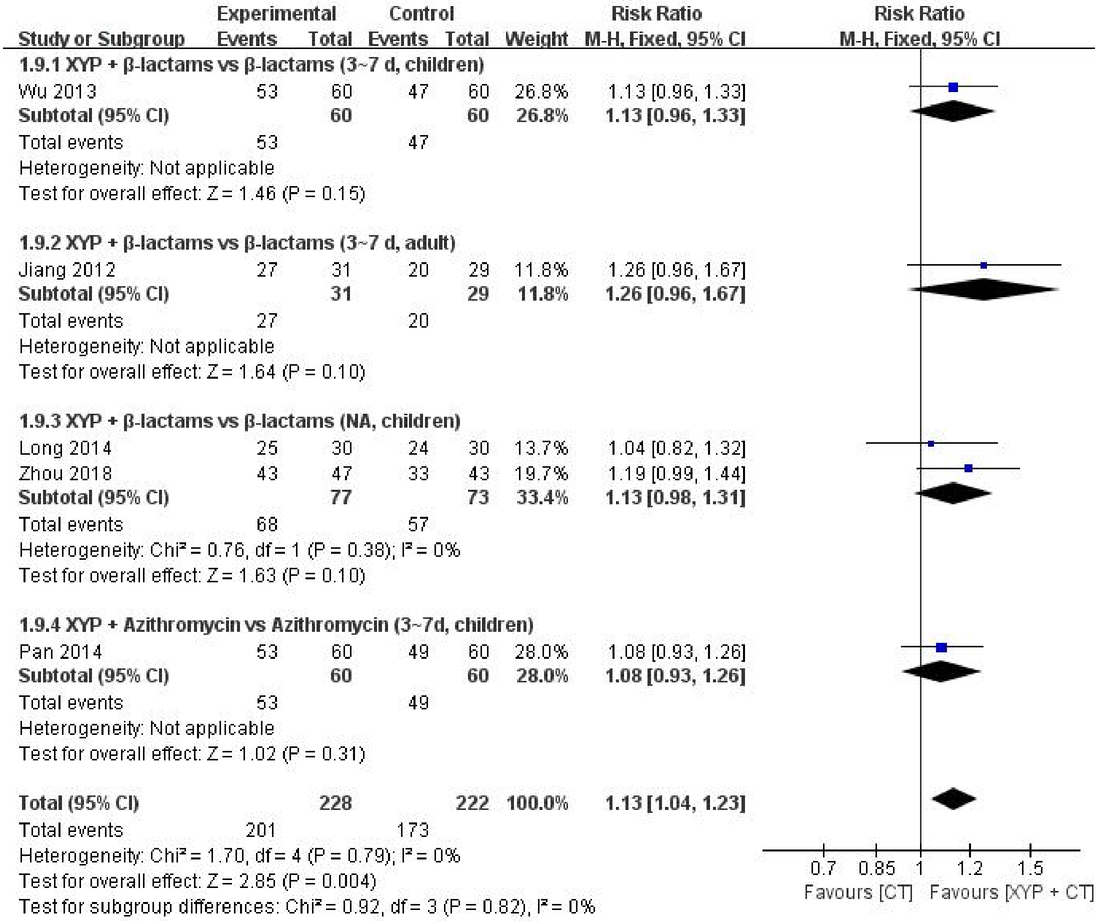

Figure 11. Forest plot of recovery rate of white blood cell count.

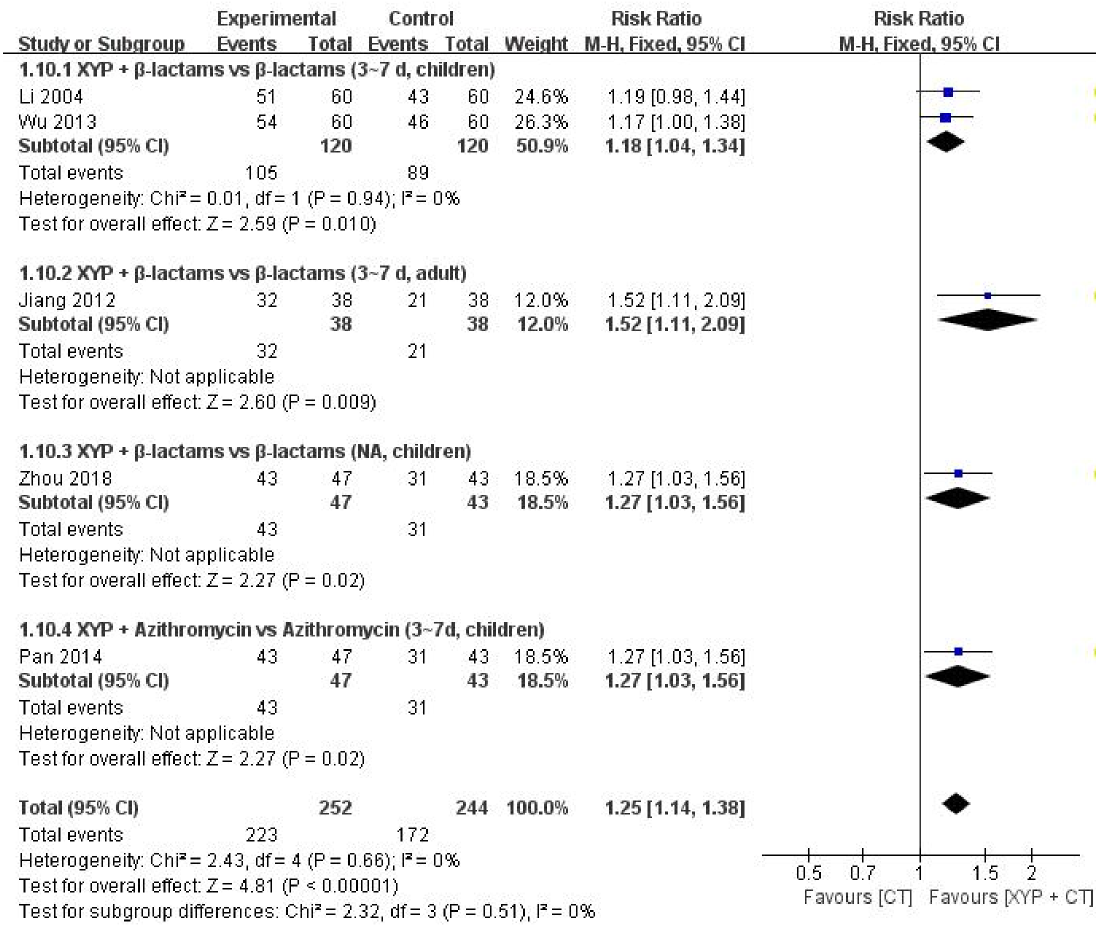

Figure 12. Forest plot of disappearance rate of tonsillar redness and swelling.

	Experimental		Control			Odds Ratio
Study or Subgroup	Events	Total	Events	Total	Weight	M-H, Random, 95% CI
**1.11.1 XYP + β-lactams vs β-lactams (3~7 d, children)**						
Liu 2020	6	43	4	43	14.8%	1.58 [0.41, 6.05]
**Subtotal (95% CI)**		**43**		**43**	**14.8%**	**1.58 [0.41, 6.05]**
Total events	6		4			
Heterogeneity: Not applicable						
Test for overall effect: Z = 0.67 (P = 0.50)						
**1.11.2 XYP + β-lactams vs β-lactams (8~14 d, children)**						
Qiao 2015	2	65	2	61	11.1%	0.94 [0.13, 6.86]
Sun 2020	11	254	33	105	18.4%	0.10 [0.05, 0.21]
**Subtotal (95% CI)**		**319**		**166**	**29.4%**	**0.25 [0.03, 2.19]**
Total events	13		35			
Heterogeneity: Tau² = 1.95; Chi² = 4.33, df = 1 (P = 0.04); I² = 77%						
Test for overall effect: Z = 1.25 (P = 0.21)						
**1.11.3 XYP + β-lactams vs β-lactams (NA, children)**						
Long 2014	3	30	2	30	11.7%	1.56 [0.24, 10.05]
**Subtotal (95% CI)**		**30**		**30**	**11.7%**	**1.56 [0.24, 10.05]**
Total events	3		2			
Heterogeneity: Not applicable						
Test for overall effect: Z = 0.46 (P = 0.64)						
**1.11.4 XYP + Clindamycin hydrochloride injection vs Clindamycin hydrochloride injection (3~7 d, children)**						
He 2013	1	57	0	57	6.3%	3.05 [0.12, 76.54]
Ren 2018	3	47	13	47	14.8%	0.18 [0.05, 0.68]
Shuai 2018	2	54	10	54	13.4%	0.17 [0.04, 0.81]
**Subtotal (95% CI)**		**158**		**158**	**34.6%**	**0.25 [0.07, 0.84]**
Total events	6		23			
Heterogeneity: Tau² = 0.33; Chi² = 2.77, df = 2 (P = 0.25); I² = 28%						
Test for overall effect: Z = 2.24 (P = 0.02)						
**1.11.5 XYP + Clindamycin hydrochloride injection vs Clindamycin hydrochloride injection (NA, children)**						
Mai 2018	1	38	3	37	9.5%	0.31 [0.03, 3.09]
**Subtotal (95% CI)**		**38**		**37**	**9.5%**	**0.31 [0.03, 3.09]**
Total events	1		3			
Heterogeneity: Not applicable						
Test for overall effect: Z = 1.00 (P = 0.32)						
**Total (95% CI)**		**588**		**434**	**100.0%**	**0.43 [0.16, 1.14]**
Total events	29		67			
Heterogeneity: Tau² = 1.21; Chi² = 21.69, df = 7 (P = 0.003); I² = 68%						
Test for overall effect: Z = 1.70 (P = 0.09)						
Test for subgroup differences: Chi² = 5.94, df = 4 (P = 0.20), I² = 32.7%						

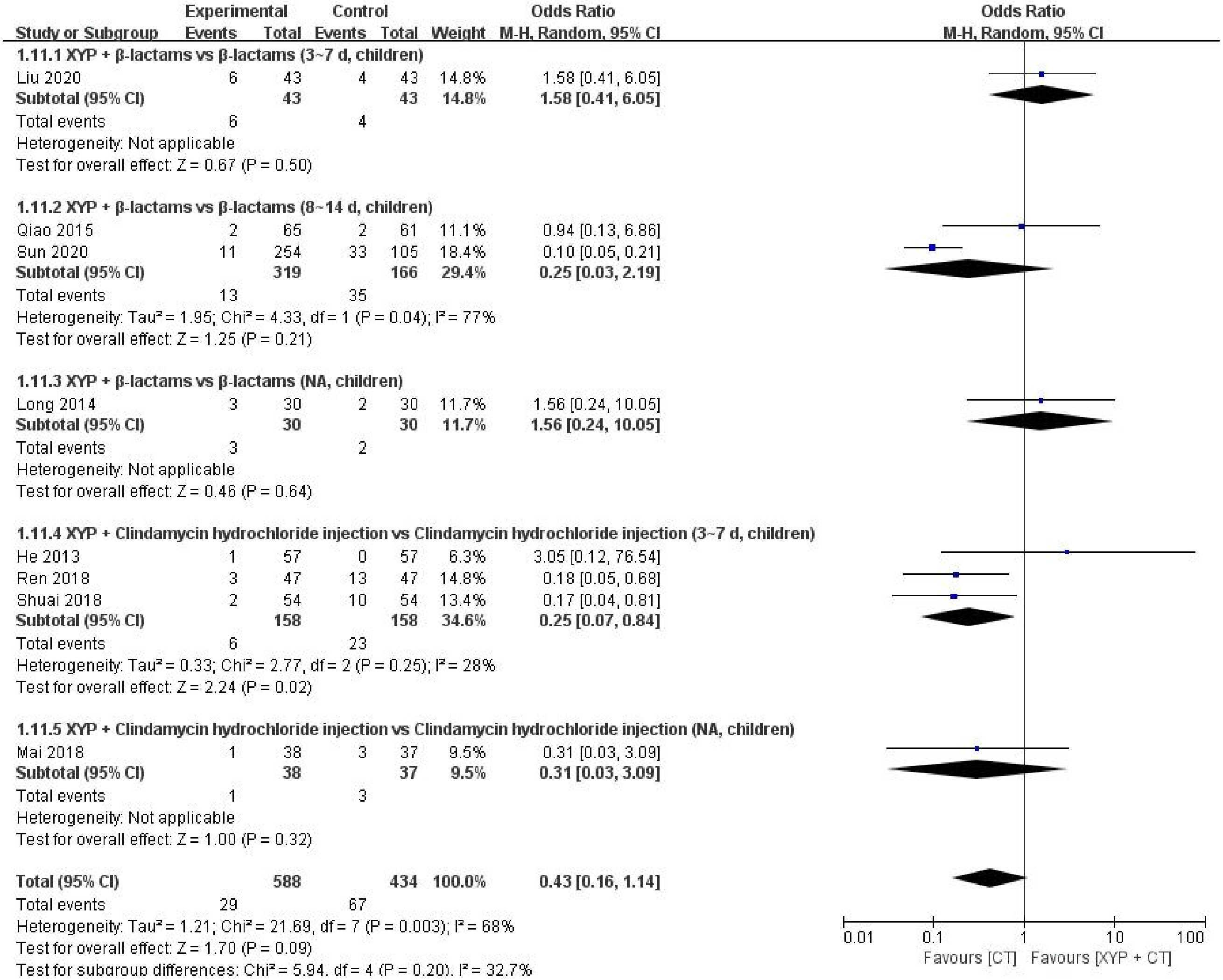

Figure 13. Forest plot of the incidence of AEs.
