# Supplementary material for: Potential efficacy and safety of Xiyanping injection as adjuvant therapy in treatment of suppurative acute tonsillitis: a meta-analysis, trial sequential analysis, and certainty of evidence
Source: Front Pharmacol. 2024 Jun 12;15:1327856. doi: 10.3389/fphar.2024.1327856 (PMC11199392; doi:10.3389/fphar.2024.1327856)
Supplement: Supplementary file 2 [file DataSheet4.PDF]

Supplementary File S2. Search strategies for databases.

	**Pubmed**
Number	Search terms
#1	("suppurative acute tonsillitis"[MeSH Terms])
#2	("Xiyanping"[Title] OR "Xi yan ping"[Title] OR "Xi-yan-ping"[Title])
#3	"randomized controlled trial"[Title/Abstract] OR "randomised controlled trial"[Title/Abstract] OR "randomized"[Title/Abstract] OR"randomised"[Title/Abstract] OR "RCT"[Title/Abstract]
#4	#1 and #2 and #3
	**Cochrane Library**
#1	(suppurative acute tonsillitis[Title/Abstract/keywords])
#2	(Xiyanping[Title/Abstract/keyword]) OR (Xi yan ping[Title/Abstract/keyword]) OR (Xi-yan-ping[Title/Abstract/keyword])
#3	(randomized controlled trial[Title/Abstract]) OR (randomised controlled trial[Title/Abstract]) OR (randomized) OR (randomised [Title/Abstract]) OR (RCT[Title/Abstract])
#4	#1 and #2 and #3
	**Web of Science**
#1	(TS = suppurative acute tonsillitis)
#2	(TS = Xiyanping OR Xi yan ping OR Xi-yan-ping)
#3	(TS = randomized controlled trial OR randomised controlled trial OR randomized OR randomised OR RCT)
#4	#1 and #2 and #3
	**Embase**
#1	(suppurative acute tonsillitis[Title/Abstract/keywords])
#2	(Xiyanping[Title/Abstract/keyword]) OR (Xi yan ping [Title/Abstract/keyword]) OR (Xi-yan-ping[Title/Abstract/keyword])

#3	(randomized controlled trial[Title/Abstract]) OR (randomised controlled trial[Title/Abstract]) OR (randomized[Title/Abstract])OR (randomised[Title/Abstract]) OR (RCT[Title/Abstract])
#4	#1 and #2 and #3

China National Knowledge Infrastructure (CNKI)

(主题=喜炎平 + 喜炎平注射液)

AND

(主题=扁桃体炎)

AND

(摘要=随机对照 + 随机 + 试验 + RCT)

China Science and Technology Journal Database (VIP)

(题名或关键词=喜炎平 + 喜炎平注射液)

AND

(题名或关键词=扁桃体炎)

AND

(摘要=随机对照 + 随机 + 试验 + RCT)

Wanfang Database (Wangfang)

(题名或关键词=喜炎平 OR 喜炎平注射液)

AND

(题名或关键词=扁桃体炎)

AND

(摘要=随机对照 OR 随机 OR 试验 OR RCT)

China Biology Medicine disc (CMB)

("喜炎平"[标题:智能] OR "喜炎平注射液"[标题:智能])

AND

("扁桃体炎"[标题:智能])

AND

("随机对照"[摘要:智能] OR "随机"[摘要:智能] OR "试验"[摘要:智能] OR "RCT"[摘要:智能]
