# Supplementary material for: Potential efficacy and safety of Xiyanping injection as adjuvant therapy in treatment of suppurative acute tonsillitis: a meta-analysis, trial sequential analysis, and certainty of evidence
Source: Front Pharmacol. 2024 Jun 12;15:1327856. doi: 10.3389/fphar.2024.1327856 (PMC11199392; doi:10.3389/fphar.2024.1327856)
Supplement: Supplementary file 3 [file DataSheet3.pdf]

SUPPLEMENTAL FILE S1

Title: Potential Efficacy and Safety of Xiyanping Injection as Adjuvant Therapy in Treatment of Suppurative Acute Tonsillitis: A Meta-analysis, Trial Sequential Analysis, and Certainty of Evidence

Contents

Figure S1. Chemical Structures of Andrographolide. (A) 2D structure.

Figure S2. Forest plot of duration of sore throat (low heterogenicity).

Figure S3. Forest plot of the duration of disappearance of tonsillar redness and swelling (low heterogenicity).

Figure S4. Forest plot of the time of tonsil purulent discharge (low heterogenicity).

Figure S5. Forest plot of the time of recovering normal temperature (low heterogenicity).

Figure S6. Forest plot of recovery rate of disease (low heterogenicity).

Figure S7. Forest plot of the incidence of adverse reactions (low heterogenicity).

Figure S8. Funnel plot of publication bias according to duration of sore throat.

Figure S9. Egger's publication bias plot according to duration of sore throat.

Figure S10. Funnel plot of publication bias according to duration of sore throat.

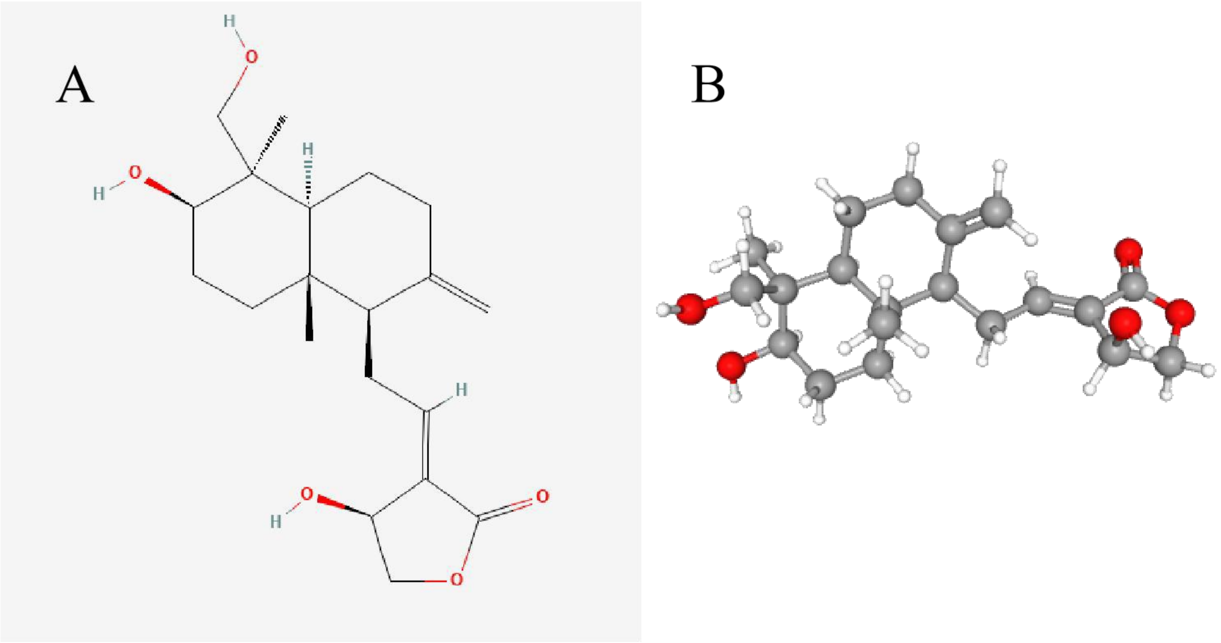

Figure S1. Chemical Structures of Andrographolide. (A) 2D structure (URL: https://pubchem. ncbi. nlm. nih. gov/compound/5318517#section=2D-Structure&fullscreen=true) (B) 3D structure (URL: https://pubchem. ncbi. nlm. nih. gov/compound/5318517#section=3D-Conformer&fullscreen=true)

Study or Subgroup	Experimental Mean	Experimental SD	Experimental Total	Control Mean	Control SD	Control Total	Weight	Mean Difference IV, Fixed, 95% CI
**1.1.1 XYP + β-lactams vs β-lactams (3~7 d, children)**								
Lyu 2020	33.12	11.04	60	45.12	18	60	1.5%	-12.00 [-17.34, -6.66]
Ou 2017	34.08	14.16	100	45.36	24.72	100	1.3%	-11.28 [-16.86, -5.70]
Shi 2018	34.32	13.92	450	45.36	24.72	450	6.0%	-11.04 [-13.66, -8.42]
Zhou 2018	34.61	1.34	47	46.73	2.16	43	73.7%	-12.12 [-12.87, -11.37]
**Subtotal (95% CI)**			**657**			**653**	**82.5%**	**-12.03 [-12.73, -11.32]**
Heterogeneity: Chi² = 0.67, df = 3 (P = 0.88); I² = 0%								
Test for overall effect: Z = 33.22 (P < 0.00001)								
**1.1.5 XYP + Clindamycin hydrochloride injection vs Clindamycin hydrochloride injection (3~7 d, children)**								
Hu 2018	73.44	12.48	54	112.56	20.4	54	1.0%	-39.12 [-45.50, -32.74]
Peng 2015	47	4	50	79	6	50	10.4%	-32.00 [-34.00, -30.00]
Zhou 2019	34.8	6	60	67.68	8.4	60	6.1%	-32.88 [-35.49, -30.27]
**Subtotal (95% CI)**			**164**			**164**	**17.5%**	**-32.72 [-34.26, -31.18]**
Heterogeneity: Chi² = 4.38, df = 2 (P = 0.11); I² = 54%								
Test for overall effect: Z = 41.63 (P < 0.00001)								
**Total (95% CI)**			**821**			**817**	**100.0%**	**-15.65 [-16.29, -15.00]**
Heterogeneity: Chi² = 577.18, df = 6 (P < 0.00001); I² = 99%								
Test for overall effect: Z = 47.59 (P < 0.00001)								
Test for subgroup differences: Chi² = 572.12, df = 1 (P < 0.00001), I² = 99.8%								

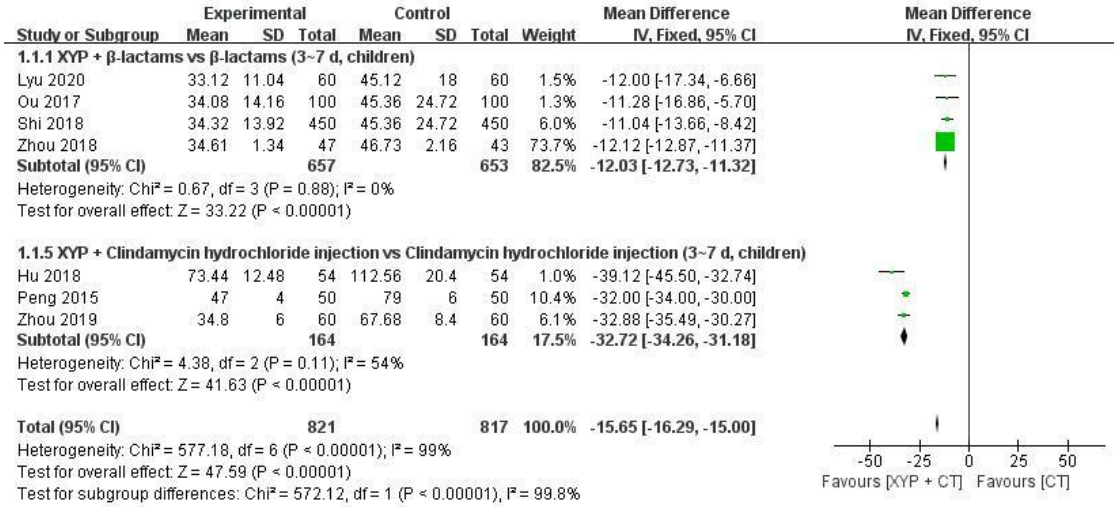

Figure S2. Forest plot of duration of sore throat (low heterogenicity).

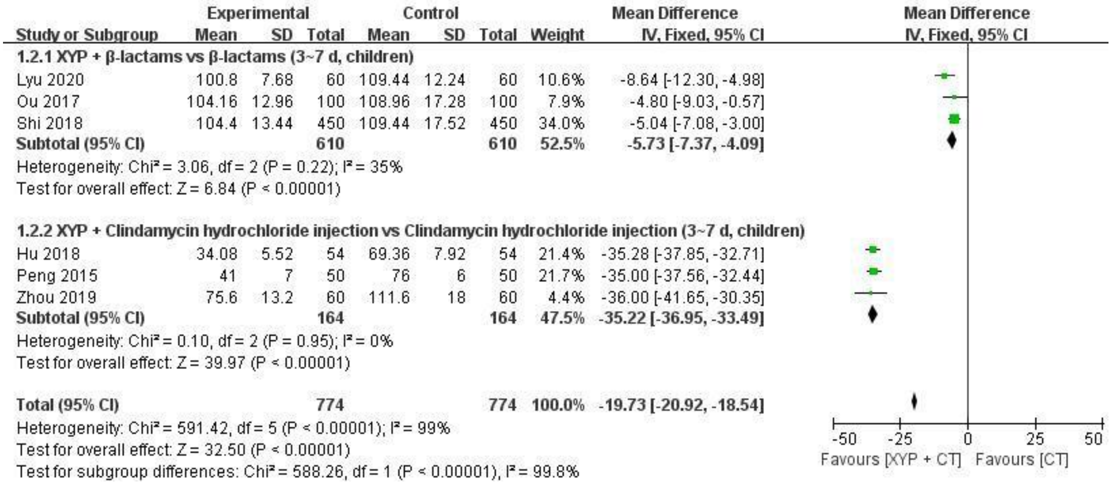

Figure S3. Forest plot of the duration of disappearance of tonsillar redness and swelling (low heterogenicity).

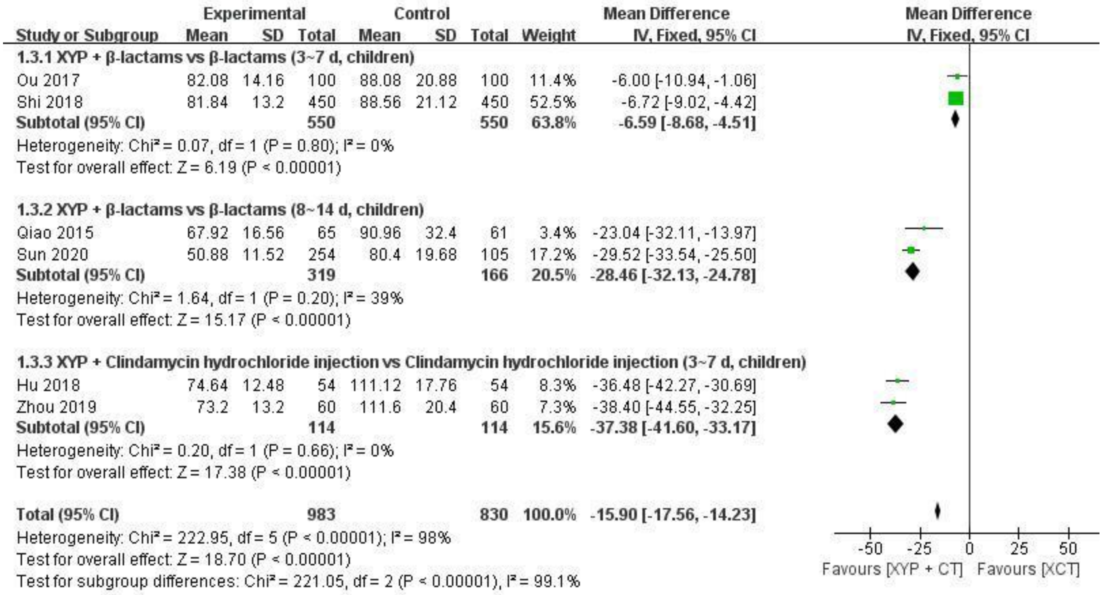

Figure S4. Forest plot of the time of tonsil purulent discharge (low heterogenicity).

Study or Subgroup	Experimental Mean	Experimental SD	Experimental Total	Control Mean	Control SD	Control Total	Weight	Mean Difference IV, Fixed, 95% CI
**1.4.1 XYP + β-lactams vs β-lactams (3~7 d, children)**								
Ou 2017	31.44	11.04	100	40.08	20.88	100	0.1%	-8.64 [-13.27, -4.01]
Shi 2018	31.92	10.8	450	39.84	21.36	450	0.6%	-7.92 [-10.13, -5.71]
Wu 2013	27.4	0.56	60	36.2	0.38	60	98.0%	-8.80 [-8.97, -8.63]
**Subtotal (95% CI)**			**610**			**610**	**98.7%**	**-8.79 [-8.97, -8.62]**
Heterogeneity: Chi² = 0.61, df = 2 (P = 0.74); I² = 0%								
Test for overall effect: Z = 101.03 (P < 0.00001)								
**1.4.3 XYP + β-lactams vs β-lactams (8~14 d, children)**								
Qiao 2015	36.24	15.6	65	60	21.12	61	0.1%	-23.76 [-30.28, -17.24]
Sun 2020	51.12	10.8	254	78	17.04	105	0.2%	-26.88 [-30.40, -23.36]
**Subtotal (95% CI)**			**319**			**166**	**0.3%**	**-26.18 [-29.27, -23.08]**
Heterogeneity: Chi² = 0.68, df = 1 (P = 0.41); I² = 0%								
Test for overall effect: Z = 16.57 (P < 0.00001)								
**1.4.5 XYP + Clindamycin hydrochloride injection vs Clindamycin hydrochloride injection (3~7 d, children)**								
Hu 2018	31.68	10.8	54	63.12	16.08	54	0.1%	-31.44 [-36.61, -26.27]
Peng 2015	37	6	50	65	4	50	0.7%	-28.00 [-30.00, -26.00]
Zhou 2019	32.4	10.8	60	63.6	15.6	60	0.1%	-31.20 [-36.00, -26.40]
**Subtotal (95% CI)**			**164**			**164**	**1.0%**	**-28.81 [-30.55, -27.07]**
Heterogeneity: Chi² = 2.58, df = 2 (P = 0.28); I² = 22%								
Test for overall effect: Z = 32.49 (P < 0.00001)								
**Total (95% CI)**			**1093**			**940**	**100.0%**	**-9.04 [-9.21, -8.87]**
Heterogeneity: Chi² = 626.57, df = 7 (P < 0.00001); I² = 99%								
Test for overall effect: Z = 104.47 (P < 0.00001)								
Test for subgroup differences: Chi² = 622.70, df = 2 (P < 0.00001), I² = 99.7%								

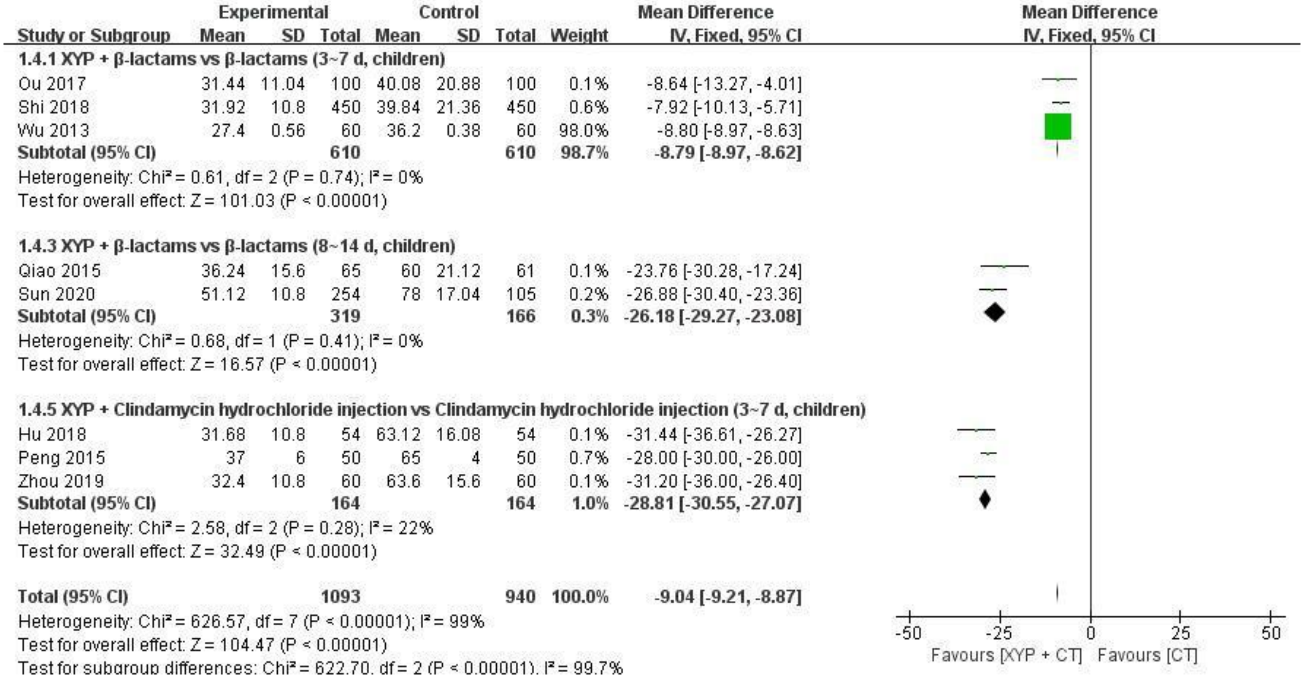

Figure S5. Forest plot of the time of recovering normal temperature (low heterogenicity).

Study or Subgroup	Experimental Events	Experimental Total	Control Events	Control Total	Weight	Risk Ratio M-H, Fixed, 95% CI
**1.7.1 XYP + β-lactams vs β-lactams (3~7 d, children)**						
Dai 2006	24	34	17	34	4.1%	1.41 [0.95, 2.11]
Li 2004	39	60	25	60	6.0%	1.56 [1.10, 2.22]
Liu 2008	21	36	10	33	2.5%	1.93 [1.07, 3.46]
Lu 2010	52	60	39	60	9.4%	1.33 [1.08, 1.65]
Ou 2017	69	100	59	100	14.2%	1.17 [0.95, 1.44]
Wu 2013	51	60	43	60	10.4%	1.19 [0.98, 1.44]
Ye 2011	38	60	24	60	5.8%	1.58 [1.10, 2.28]
Zou 2010	23	30	15	30	3.6%	1.53 [1.02, 2.31]
**Subtotal (95% CI)**		**440**		**437**	**56.0%**	**1.36 [1.23, 1.51]**
Total events	317		232			
Heterogeneity: Chi² = 6.95, df = 7 (P = 0.43); I² = 0%						
Test for overall effect: Z = 5.82 (P < 0.00001)						
**1.7.3 XYP + Clindamycin hydrochloride injection vs Clindamycin hydrochloride injection (3~7 d, children)**						
Dong 2009	20	42	14	40	3.5%	1.36 [0.80, 2.31]
Gu 2019	15	45	8	45	1.9%	1.88 [0.88, 3.98]
He 2013	28	57	10	57	2.4%	2.80 [1.50, 5.21]
Hu 2018	36	54	27	54	6.5%	1.33 [0.96, 1.85]
Luo 2016	24	49	9	49	2.2%	2.67 [1.38, 5.14]
Peng 2015	35	50	22	50	5.3%	1.59 [1.11, 2.28]
Ren 2018	23	47	14	47	3.4%	1.64 [0.97, 2.78]
Shuai 2018	18	54	10	54	2.4%	1.80 [0.92, 3.53]
Wang 2015	23	39	15	39	3.6%	1.53 [0.95, 2.47]
Zhou 2019	34	60	23	60	5.5%	1.48 [1.00, 2.18]
**Subtotal (95% CI)**		**497**		**495**	**36.7%**	**1.68 [1.44, 1.96]**
Total events	256		152			
Heterogeneity: Chi² = 7.78, df = 9 (P = 0.56); I² = 0%						
Test for overall effect: Z = 6.59 (P < 0.00001)						
**1.7.4 XYP + Ribavirin vs Ribavirin (3children)**						
Gan 2020	19	30	11	30	2.7%	1.73 [1.00, 2.97]
Gong 2020	33	62	19	62	4.6%	1.74 [1.12, 2.70]
**Subtotal (95% CI)**		**92**		**92**	**7.2%**	**1.73 [1.23, 2.44]**
Total events	52		30			
Heterogeneity: Chi² = 0.00, df = 1 (P = 0.99); I² = 0%						
Test for overall effect: Z = 3.14 (P = 0.002)						
**Total (95% CI)**		**1029**		**1024**	**100.0%**	**1.50 [1.38, 1.64]**
Total events	625		414			
Heterogeneity: Chi² = 22.49, df = 19 (P = 0.26); I² = 16%						
Test for overall effect: Z = 9.35 (P < 0.00001)						
Test for subgroup differences: Chi² = 5.89, df = 2 (P = 0.05), I² = 66.0%						

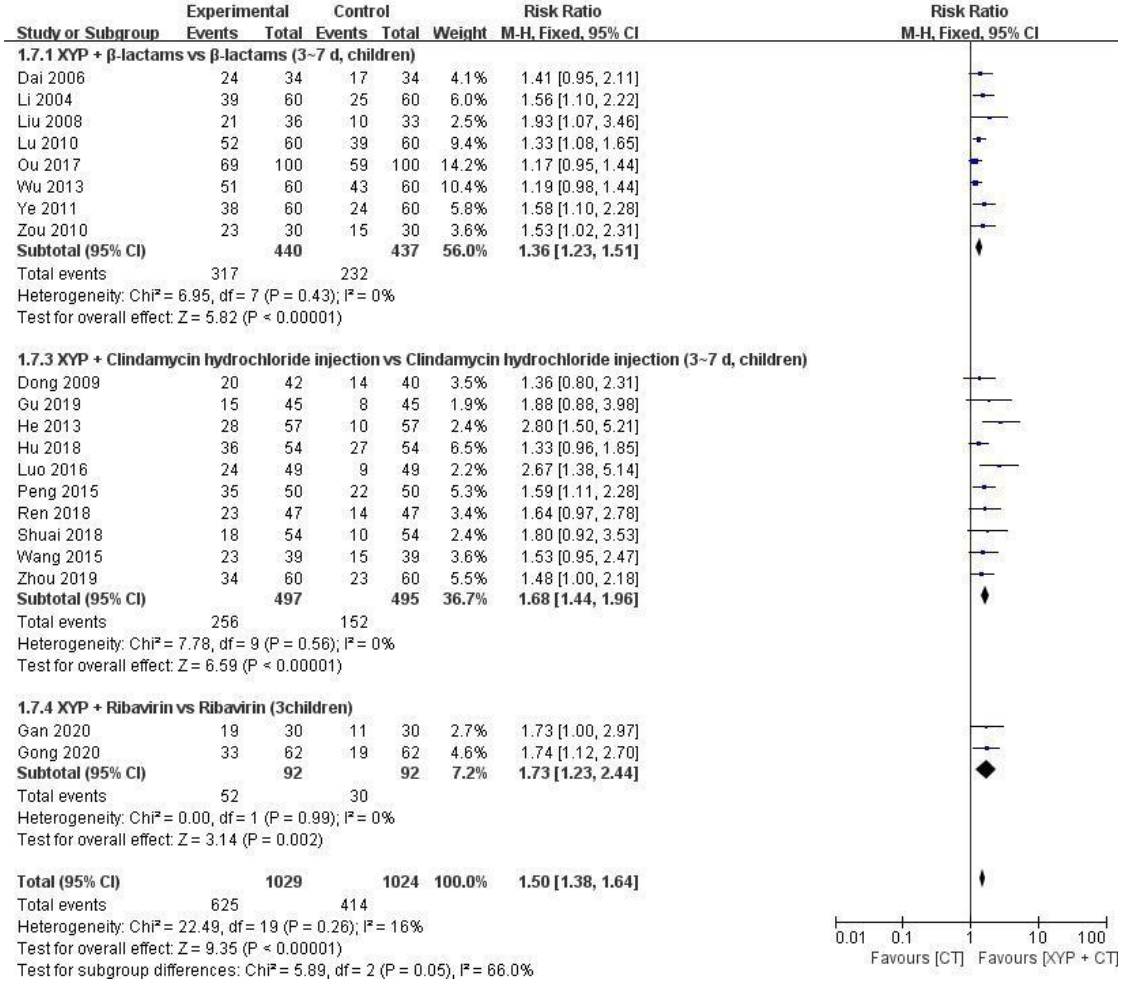

Figure S6. Forest plot of recovery rate of disease (low heterogenicity).

Study or Subgroup	Experimental Events	Experimental Total	Control Events	Control Total	Weight	Odds Ratio M-H, Fixed, 95% CI
**1.11.4 XYP + Clindamycin hydrochloride injection vs Clindamycin hydrochloride injection (3~7 d, children)**						
He 2013	1	57	0	57	2.2%	3.05 [0.12, 76.54]
Ren 2018	3	47	13	47	54.6%	0.18 [0.05, 0.68]
Shuai 2018	2	54	10	54	43.2%	0.17 [0.04, 0.81]
**Subtotal (95% CI)**		**158**		**158**	**100.0%**	**0.24 [0.10, 0.58]**
Total events	6		23			
Heterogeneity: Chi² = 2.77, df = 2 (P = 0.25); I² = 28%						
Test for overall effect: Z = 3.13 (P = 0.002)						
**Total (95% CI)**		**158**		**158**	**100.0%**	**0.24 [0.10, 0.58]**
Total events	6		23			
Heterogeneity: Chi² = 2.77, df = 2 (P = 0.25); I² = 28%						
Test for overall effect: Z = 3.13 (P = 0.002)						
Test for subgroup differences: Not applicable						

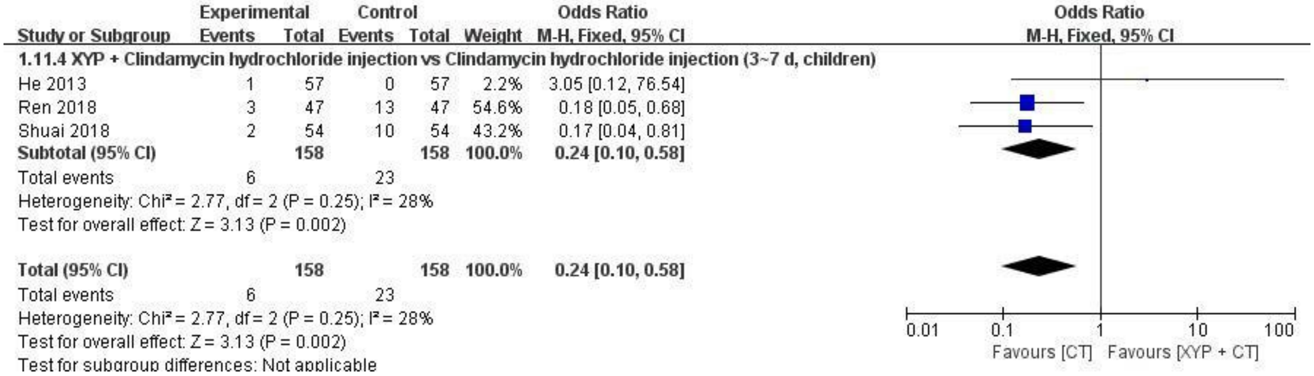

Figure S7. Forest plot of the incidence of adverse reactions (low heterogenicity).

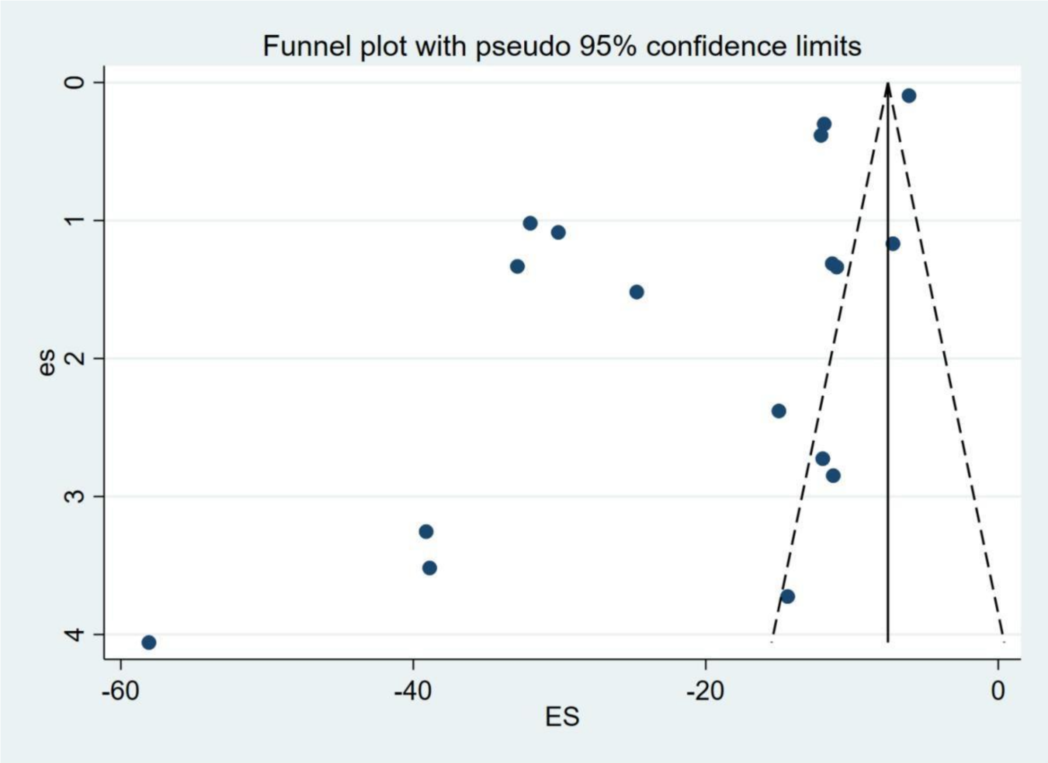

Figure S8. Funnel plot of publication bias according to duration of sore throat.

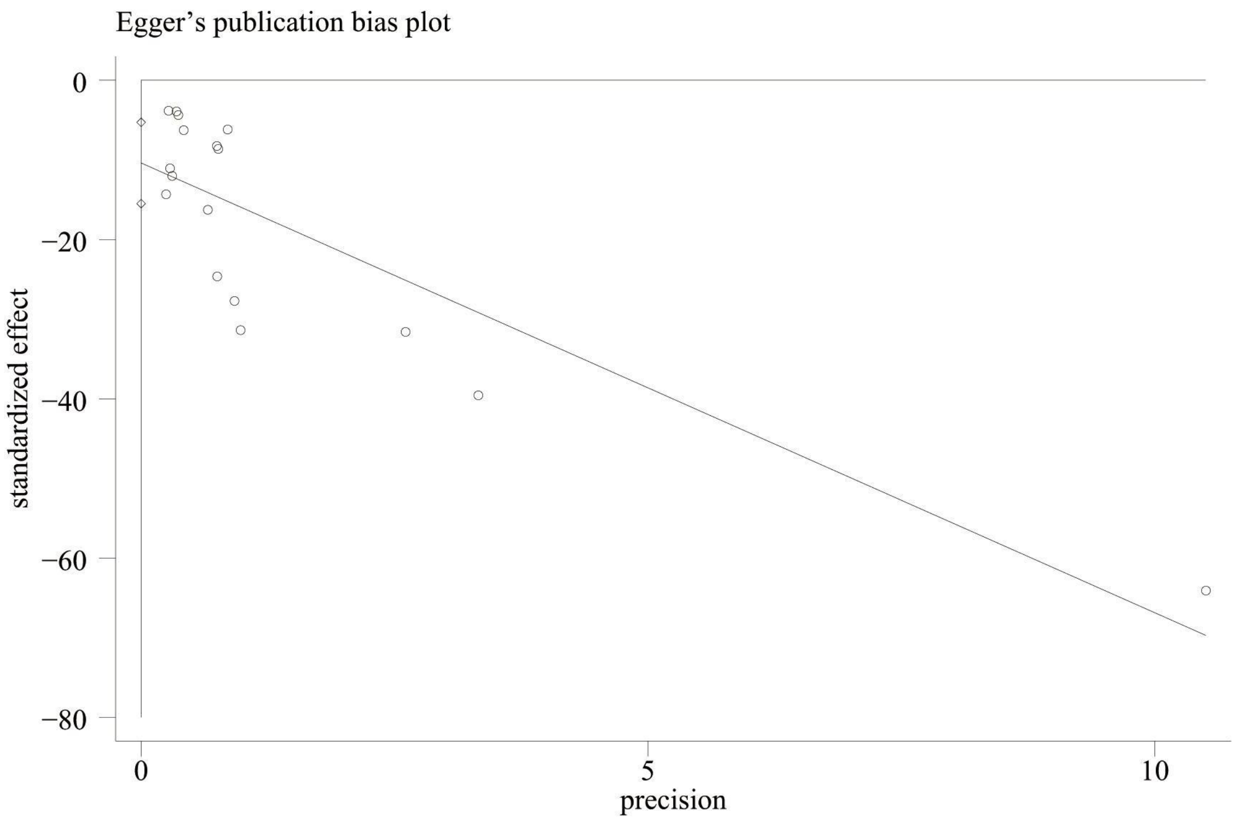

Figure S9. Egger's publication bias plot according to duration of sore throat.

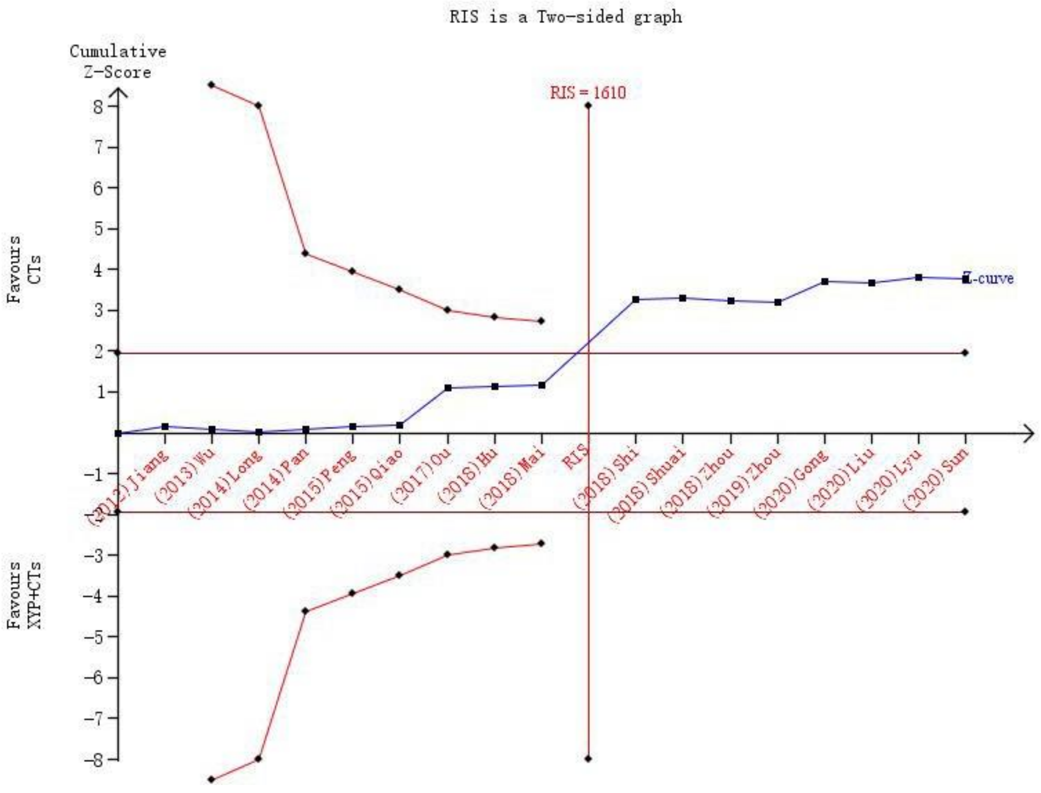

Figure S10. Funnel plot of publication bias according to duration of sore throat.
