# Supplementary material for: Potential efficacy and safety of Xiyanping injection as adjuvant therapy in treatment of suppurative acute tonsillitis: a meta-analysis, trial sequential analysis, and certainty of evidence
Source: Front Pharmacol. 2024 Jun 12;15:1327856. doi: 10.3389/fphar.2024.1327856 (PMC11199392; doi:10.3389/fphar.2024.1327856)
Supplement: Supplementary file 5 [file DataSheet5.PDF]

Supplementary File S3. Checklist of Items for Reporting Trials of Chinese Herbal Medicine Formulas

Table S1. Checklist of Items for Reporting Trials of Chinese Herbal Medicine Formulas 1.

Table S2. Checklist of Items for Reporting Trials of Chinese Herbal Medicine Formulas 2.

Table S3. Checklist of Items for Reporting Trials of Chinese Herbal Medicine Formulas 3.

Table S4. Checklist of Items for Reporting Trials of Chinese Herbal Medicine Formulas 4.

Table S1. Checklist of Items for Reporting Trials of Chinese Herbal Medicine Formulas 1.

Table. Checklist of Items for Reporting Trials of Chinese Herbal Medicine Formulas*Section/Topic	Item Number	Standard CONSORT Checklist Item	Qiao et al. (2015)	Mai (2018)	Ren (2018)	Liu (2020)	Lu (2010)	Ye (2011)	Lyu (2020)	Wu (2013)
Title, abstract, and keywords	1a	Identification as a randomized trial in the title	Clinical study of Xiyanping injection in the treatment of children with acute suppurative tonsillitis (No TCM Pattern)							
	1b	Structured summary of trial design, methods, results, and conclusions (for specific guidance, see CONSORT for abstracts	Objective: To evaluate the clinical efficacy of Xiyanping injection in the treatment of acute suppurative tonsillitis. Methods: Researchers conducted a single-blind, randomized, controlled trials involving 126 patients with acute suppurative tonsillitis	Objective: To evaluate the clinical efficacy of Xiyanping injection in the treatment of acute suppurative tonsillitis. Methods: Researchers conducted a single-blind,	Objective: To evaluate the clinical efficacy of Xiyanping injection in the treatment of acute suppurative tonsillitis. Methods: Researchers conducted a single-blind,	Objective: To evaluate the clinical efficacy of Xiyanping injection in the treatment of acute suppurative tonsillitis. Methods: Researchers conducted a single-blind, randomized, controlled trials involving 86 patients with acute	Objective: To evaluate the clinical efficacy of Xiyanping injection in the treatment of acute suppurative tonsillitis. Methods: Researchers conducted a single-blind,	Objective: To evaluate the clinical efficacy of Xiyanping injection in the treatment of acute suppurative tonsillitis. Methods: Researchers conducted a single-blind,	Objective: To evaluate the clinical efficacy of Xiyanping injection in the treatment of acute suppurative tonsillitis. Methods: Researchers conducted a single-blind,	Objective: To evaluate the clinical efficacy of Xiyanping injection in the treatment of acute suppurative tonsillitis. Methods: Researchers conducted a single-blind,

				randomized, controlled trials involving 75 patients with acute suppurative tonsillitis	randomized, controlled trials involving 94 patients with acute suppurative tonsillitis	suppurative tonsillitis	randomized, controlled trials involving 120 patients with acute suppurative tonsillitis	randomized, controlled trials involving 120 patients with acute suppurative tonsillitis	randomized, controlled trials involving 120 patients with acute suppurative tonsillitis	randomized, controlled trials involving 120 patients with acute suppurative tonsillitis
	1c	Determination of appropriate keywords, including “Chinese herbal medicine formula” and “randomized controlled trial”	Determination of appropriate keywords, including “Xiyanping injection” and “randomized controlled trial”							
Introduction Background and objectives	2a	Scientific background and explanation of rationale	Xiyanping injection (XYP), a purifying agent of TCM andrographis paniculata (Chuan Xin Lian in chinese, CXL), is a popular traditional medicinal plant. Previous studies confirmed that andrographolide was identified as safe, efficacious anti-inflammatory agent and had effects on disease of respiratory system included upper respiratory tract infections, lung injury, pneumonia, and other respiratory disease. Andrographolide can decrease the production of pro-inflammatory factors. It has antibacterial, antiviral, antipyretic and anti-inflammatory effects. Several basic researches have confirmed that andrographolide can exert anti-inflammatory effects through multiple targets (such as targets factor-α, interleukin, etc.) and multiple pathways (such as regulating silencing information regulator 1/extracellular regulatory kinase, the expression and activation of nuclear transcription factor-κB, etc.). A number of randomized controlled trial studies showed that XYP have good therapeutic effect in treatment of suppurative acute tonsillitis (SAT).							

	2b	Specific objectives or hypotheses	A single-blind, randomized, controlled clinical trial has been conducted to test the efficacy and safety of Xiyanping injection in the treatment of acute suppurative tonsillitis.							
Methods										
Trial design	3a	Description of trial design (such as parallel, factorial), including allocation ratio	This trial was a single-center, single-blind, randomized controlled clinical study conducted at Chongzhou People's Hospital, Chengdu, China.	This trial was a single-center, single-blind, randomized controlled clinical study conducted at People's Hospital of Hezheng County, Gansu province, China.	This trial was a single-center, single-blind, randomized controlled clinical study conducted at Gaotai Renji Hospital, Gansu province, China.	This trial was a single-center, single-blind, randomized controlled clinical study conducted at Nancheng County People's Hospital, Jiangxi province, China.	This trial was a single-center, single-blind, randomized controlled clinical study conducted at Bin county People's Hospita, Heilongjiang Province, China.	This trial was a single-center, single-blind, randomized controlled clinical study conducted at Shilong Boai Hospital, dongguan city, Guangdong province, China.	This trial was a single-center, single-blind, randomized controlled clinical study conducted at Jilin Oilfield Hospital, Jilin province, China. ,	This trial was a single-center, single-blind, randomized controlled clinical study conducted at the Second People's Hospital of Fengrun District, Tangshan city, Hebei province, China.
	3b	Important changes to methods after trial commencement	There were no important changes to methods after trial commencement.							

		(such as eligibility criteria), with reasons								
Participants	4a	Eligibility criteria for participants	Inclusion criteria: body temperature ≥38℃; Sore throat or difficulty swallowing; Diffuse hyperemia of the pharyngeal mucosa, enlarged and hyperemic tonsils, yellow-white pus spots on the surface or yellow-white exudates at the crypt, which could be connected into a pseudomembrane-like shape, easily wiped, but without bleeding; White blood cell count > 10.0×10*9 / L; The time from fever to admission was 2 to 3 days. No other antibiotics were used before treatment.	Inclusion Criteria (1) All patients had been diagnosed with acute suppurative tonsillitis. (2) The children were not allergic to the drugs used in this study. (3) The study was approved by the ethics committee of the hospital and the patient's family	Clinical manifestations included fever, pharyngeal pain, tonsil enlargement, white blood cell count higher than 10.0×109/L, and neutrophil ratio higher than 50%. Patients who were allergic to the drugs used in this study, had used related drugs or received related	1) Inclusion criteria :① All patients met the diagnostic criteria of tonsillitis in the 8th edition of Zhufusang Practical Pediatrics, accompanied by purulent secretion, tonsil enlargement and other symptoms; ② The families of the selected children voluntarily signed the consent form; ③ meeting the "TCM diagnostic criteria for Disease and Syndrome", the main symptoms were sore throat, mild	The specific diagnostic criteria, inclusion and exclusion criteria were not listed in this study	The specific diagnostic criteria, inclusion and exclusion criteria were not listed in this study.	All the children were in accordance with the relevant diagnostic criteria in Zhu Fu Tang Practical Pediatrics	The specific diagnostic criteria, inclusion and exclusion criteria were not listed in this study.

Exclusion criteria: severe complications such as sepsis; Severe immunodeficiency or combined chronic diseases; Patients with a history of penicillin anaphylactic shock or other severe allergies.	It is a signed consent. Exclusion Criteria (1) Children with other upper respiratory tract infections were excluded. (2) Infants with feeding dysfunction were excluded; (3) Patients with incomplete clinical data were excluded.	treatment before treatment, had severe liver or renal insufficiency, and had systemic infectious diseases were excluded. Combined with immune system disorders, coagulation disorders, etc.	dysphagia, onset of lactoid moth, tonsil congestion; Secondary symptoms: fever, cough, expectoration; Tongue pulse: tongue coating thin white, pulse floating number. (2) Exclusion criteria :① accompanied with severe immunodeficiency, severe malnutrition, etc. ② Allergic to Xiyanping injection or preparations containing andrographolide total sulfonate or a history of serious adverse reactions; ③ Intolerance to

					amoxicillin and clavulanate potassium for injection.				
4b	Settings and locations where the data were collected	The study was conducted from January 2013 to May 2014 at Chongzhou People's Hospital, Chengdu City, located in Sichuan Province, China.	The study was conducted from May 2017 to May 2018 at People's Hospital of Hezheng County, located in Gansu Province, China.	The study was conducted from From October 2015 to December 2016 at Gaotai Renji Hospital, located in Gansu Province, China.	The study was conducted from From July 2018 to December 2019 at Nancheng County People's Hospital, located in Jiangxi province, China.	The study was conducted from from June 2008 to January 2010 at Bin county People's Hospital, located in Heilongjiang province, China.	The study was conducted from from June 2008 to January 2010 at Dongguan Shilong Boai Hospital, located in Guangdong province, China.	The study was conducted at Dongguan Shilong Boai Hospital, located in Guangdong province, China.	The study was conducted from June 2010 to June 2012 at the Second People's Hospital of Fengrun District, located in Hebei province, China.

Interventions	5	The interventions for each group with sufficient details to allow replication, including how and when they were actually administered	Children in both groups were given intravenous infusion of β-lactam antibiotics. In the observation group, 23 cases were treated with amoxicillin clavulanate potassium, 3 cases with piperacillin tazobactam, 10 cases with cefotezole, 16 cases with cefuroxime, 6 cases with meclocillin sodium sulbactam, 2 cases with cefotaxime, and 2 cases with cefathiamidine. On the basis of the above treatment, Xiyanping injection (produced by Jiangxi Qingfeng Pharmaceutical Co., LTD.) was given at the same time. Batch number 2014072103, specification 50mg/ tube) 5mg/ (kg·d), diluted	The control group was given routine treatment, specifically clindamycin injection (specification: 2ml: 0.15g; H20054699; Manufacturer: Beijing Shuanglu Pharmaceutica l Co., LTD.) once a day, and the dosage depends on the weight of the child. The observation group was given Xiyanping injection (specification:	The control group was treated with clindamycin alone. Intravenous infusion of clindamycin hydrochloride injection according to body weight (manufacturer: Yichang Renfu Pharmaceutica l Co., LTD.; Approval number: H20058609; Batch number: 20150906; Specification: 8ml:0.6g), 15mg/ (kg*d), divided into 3	Both groups were given anti-infection and diet guidance (eat more heat-clearing and detoxing food, drink more water, etc.). The control group was treated with amoxicillin and clavulanate potassium for injection (amoxicillin 1g, clavulanate 0.2g), 30mg/kg, dissolved in 100mL sodium chloride injection, 3-4 times /d, intravenous drip, treatment for 5 days. The observation group was treated with amoxicillin clavulanate potassium for	Both groups were given symptomatic treatment such as cooling and hormone, and appropriate intravenous fluid infusion was given according to food intake and body temperature. The control group was given amoxicillin and clavulanate potassium 30mg/kg mixed with	Both groups were treated with intravenous penicillin injection, the dose was 200-300 000 U/kg·d, and the intravenous drip was divided into 2 times. In the treatment group, 5% glucose injection was added to Xiyanping injection, and the dose was 0.2-0.4 mL/kg·d. Both groups were given	Children in group A received routine basic treatment, including standardized diet, antipyretic, fluid infusion, and intravenous injection of β-lactam antibiotics, which lasted for 5 days. Under the above basic treatment, group B received Xiyanping injection (manufacturer: Jiangxi	The control group was given mezlocillin sulbactam sodium 75mg/ (kg·d),2 times /d; The treatment group was treated with Xiyanping injection 0.2-0.4 mL/ (kg·d) (maximum daily dose not more than 10mL) (Jiangxi Qingfeng Pharmaceutica l Co., LTD.) on the basis of the treatment of the control

	with 100ml 5% glucose injection, intravenous infusion, qd. In the control group, 20 cases were treated with amoxicillin and clavulanate potassium, 2 cases with piperacillin and tazobactam, 9 cases with cefotezole, 18 cases with cefuroxime, 8 cases with mezlocillin sodium and sulbactam, 1 case with cefazoxime, and 3 cases with cefathiamidine. No Xiyanpin injection was used in the control group. The course of treatment was 7-10 days. The two groups were given symptomatic treatment of cooling, fever and cough.	2ml:50mg; Chinese medicine approval: Z20026249; The drug was added to 100mL0.9Nacl solution by intravenous drip once a day at a dose of 5-15mg/Kg.	infusions. The study group was treated with Xiyanping on the basis of the control group. Xuxiyanping injection (Manufacturer: Jiangxi Qingfeng Pharmaceutical Co., LTD.; Approval number: Z20026249; Production batch number: 20150902; 250mg) 250mg was added into 5% glucose injection for intravenous drip, once a	injection + Xiyanping injection, amoxicillin clavulanate potassium for injection was the same as the control group, and Xiyanping injection (specification :50mg / each) was added. The treatment method was Xiyanping injection 5mg/kg+5% glucose injection 100mL, intravenous drip, 1 time /d, treatment for 5 days.	0.9% sodium chloride injection 100nd intravenous drip. Every 8 hours. The treatment group was treated with Xiyanping injection 5-10m/ (kg·d) and 5% glucose injection 250ml on the basis of the control group, once a day. Both groups were treated for 7 days.	symptomatic and supportive treatment, and no other antibiotics were used. The treatment time was 5-7 days.	Qingfeng Pharmaceutical Co., LTD., approval number: Z20026249) by intravenous drip, the dosage was 5-10 mg/kg, the dosage was 30-40 drops per minute, once a day, and the treatment lasted for 5 days.	group. Both groups were treated for 5 days as a course of treatment, and both groups were treated with rehydration, antipyretic symptomatic and supportive treatment

	day. Both groups were treated continuously for 3 days.	

Outcomes	6a	Completely defined, prespecified primary and secondary outcome measures, including how and when they were assessed	the time of recovering normal temperature, duration of sore throat, the time of tonsil purulent discharge, incidence of adverse events, recovery rate of disease,	recovery rate of disease, incidence of adverse events, the time of recovering normal temperature, duration of sore throat, duration of disappearance of tonsillar redness and swelling, the time of tonsil purulent discharge	recovery rate of disease, incidence of adverse events	incidence of adverse events, duration of sore throat, the time of tonsil purulent discharge, duration of disappearance of tonsillar redness and swelling, interleukin-6, TNF－α	recovery rate of disease,	the time of recovering normal temperature, the time of tonsil purulent discharge, recovery rate of disease,	recovery rate of disease, duration of sore throat, duration of disappearance of tonsillar redness and swelling, length of hospitliztion	, the disappearance rate of tonsillar redness and swelling, the recovery rate of white blood cell count, duration of sore throat
	6b	Any changes to trial outcomes after the trial commenced, with reasons	No important changes to methods after trial commencement.							

Sample size	7a	How sample size was determined	No specific sample size determination method was recorded.
	7b	When applicable, explanation of any interim analyses and stopping guidelines	Not Applicable
Randomization Sequence generation	8a	Method used to generate the random allocation sequence	All included studies were RCTs, thirteen trials (Qiao et al., 2015; Mai, 2018; Ren, 2018; Liu, 2020; Sun, 2020; Peng et al., 2015; Li, 2017; Shi, 2018; Luo, 2016; Hu et al., 2018; Dong, 2009; Ou et al., 2017; Liu et al., 2008; Gan et al., 2020) report the specific method of randomization. One trial (Sun, 2020) used the stratified randomization. Eight trials (Qiao et al., 2015; Ren, 2018; Liu, 2020; Peng et al., 2015; Li, 2017; Shi, 2018; Luo, 2016; Ou et al., 2017) used random number tables. Three trials (Mai, 2018; Hu et al., 2018; Gan et al., 2020) used treatment modalities. Three studies were categorized according to treatment modalities. Three trials were grouped by treatment modalities. One study (Dong, 2009) described using odd or even admission number sequence.
	8b	Type of randomization; details of any restriction (such as blocking and block size)	No specific type of randomisation was recorded.

Allocation concealment mechanism	9	Mechanism used to implement the random allocation sequence (such as sequentially numbered containers), describing any steps taken to conceal the sequence until interventions were assigned	No specific mechanism used to implement the random allocation sequence was recorded.
Implementation	10	Who generated the random allocation sequence, who enrolled participants, and who assigned participants to interventions	No specific method used to generate the random allocation sequence was recorded.

Blinding	11a	If done, who was blinded after assignment to interventions (for example, participants, care providers, those assessing outcomes) and how	No specific blinding protocol was recorded.
	11b	If relevant, description of the similarity of interventions	Not relevant.

Statistical methods	12a	Statistical methods used to compare groups for primary and secondary outcomes	PEMS3.1 for Windows statistical software was used for data processing. t test was used for measurement data, and χ2 test was used for count data.	SPSS20.0 statistical software was used for data statistics. The t test was performed by (x±s) for measurement data, and the x2 test was performed by n,% for count data. P<0.05 was considered as statistically significant.	SPSS17.0 software was used for statistical analysis, the count data were expressed as percentage (%), the difference between groups was analyzed by X2 test, when P < 0.05, the difference was statistically significant.	SPSS22.0 software was used for data processing, and measurement data were expressed as x±s. Independent sample t test was used between groups, paired sample t test was used within groups, enumeration data were expressed as percentage, and x2 test was used. P < 0.05 was considered statistically significant.	Statistical software SPSS 13.5 was used to process the data, and chi-square test was used. P<0.05 was considered statistically significant.	No specific statistical analysis method was recorded.	SPSS20.0 software program was used to process the research data, and the count data were output in the form of [n (%)] after chi-square test. The t test measurement data were output in the form of (x±s), P < 0.05, indicating that the difference between the data was large and statistically significant.	SPSS11.0 statistical software was used for statistical analysis. Measurement data were expressed as mean ± SD, and t test was used for comparison between groups. The count data were expressed as rate (%), and the comparison between groups was analyzed by chi-square test. P<0.05 was considered

			statistically significant.
	12b	Methods for additional analyses, such as subgroup analyses and adjusted analyses	Subgroup and adjusted analyses were not performed.
Results			

Participant flow (a diagram is strongly recommended)	13a	For each group, the numbers of participants who were randomly assigned, received intended treatment, and were analyzed for the primary outcome	See Table 1 for details.
	13b	For each group, losses and exclusions after randomization, together with reasons	No study reported the number of patients dropping out.
Recruitment	14a	Dates defining the periods of recruitment and follow-up	The follow-up time was the same as the treatment time, and the treatment time is shown in Table 1.

	14b	Why the trial ended or was stopped	Not applicable, none of the included studies were interrupted or stopped.
Baseline data	15	A table showing baseline demographic and clinical characteristics for each group	All the studies presented baseline data, including baseline demographic and clinical characteristics, for each group with the use of a table or text description.
Numbers analyzed	16	For each group, number of participants (denominator) included in each analysis and whether the analysis was by original assigned groups	The analysis was by original assigned groups.

Outcomes and estimation	17a	For each primary and secondary outcome, results for each group, and the estimated effect size and its precision (such as 95% confidence interval)	Not listed.
	17b	For binary outcomes, presentation of both absolute and relative effect sizes is recommended	Not listed.
Ancillary analyses	18	Results of any other analyses performed, including subgroup analyses and adjusted	Subgroup and adjusted analyses were not performed.

		analyses, distinguishing prespecified from exploratory	
Harms	19	All important harms or unintended effects in each group (for specific guidance, see CONSORT for harms [28])	See Table 1 for details.
Discussion			
Limitations	20	Trial limitations; addressing sources of potential bias; imprecision; and, if relevant, multiplicity of analyses	There were no trial limitations, addressing sources of potential bias and imprecision.

Generalizability	21	Generalizability (external validity, applicability) of the trial findings	The trial demonstrated the efficacy and safety of XYP combined with β-lactams in the treatment of children with SAT.	The trial demonstrated the efficacy and safety of XYP combined with Clindamycin hydrochloride injection in the treatment of children with SAT.	The trial demonstrated the efficacy and safety of XYP combined with Clindamycin hydrochloride injection in the treatment of children with SAT.	The trial demonstrated the efficacy and safety of XYP combined with β-lactams in the treatment of children with SAT.	The trial demonstrated the efficacy and safety of XYP combined with β-lactams in the treatment of children with SAT.	The trial demonstrated the efficacy and safety of XYP combined with β-lactams in the treatment of children with SAT.	The trial demonstrated the efficacy and safety of XYP combined with β-lactams in the treatment of children with SAT.	The trial demonstrated the efficacy and safety of XYP combined with β-lactams in the treatment of children with SAT.

Interpretation	22	Interpretation consistent with results, balancing benefits and harms, and considering other relevant evidence	Suppurative acute tonsillitis in children is knowned as “Lan Ru E” in chinese. The onset of SAT is rapid, and it is mostly caused by the invasion of tonsil by fire and heat evil in theory of traditional Chinese medicine (TCM). TCM andrographis paniculata is the component of XYP. The efficacy of Chuan Xin Lian (CXL) is to clear heat and detoxify, cool blood, and detumescence. CXL can be used for the treatment of cold and fever, sore throat, mouth and tongue sores, cough, diarrhea and dysentery, hot and astringent pain, carcarols and sores, snake bites. Along with clinical use, the activity and mechanism of XYP has also been evaluated in experimental model systems. Investigations by Fan et al. showed that XYP, a new candidate of antiviral drug for DENV infection, can inactivate DENV2 and inhibit the entry of DENV2 into cells. XYP can significantly inhibit the replication of DENV2 in a dose-dependent manner and reduce the viral load in organs and sera, and improve the survival rate of experimental mice. The antivirus activity of XYP is superior to ribavirin injection in the early and late stage of virus infection (Fan et al., 2020). In terms of safety, investigations by Yu et al. showed that compared with the control group, drugs in the group of XYP for injection 400mg/kg, 200mg/kg and 100mg/kg, XYP had no significant effect on the central nervous systems, cardiovascular and respiratory systems of experimental animals (Yu et al., 2009). Investigations by Liu et al. showed that actual phagocytosis rate and phagocytic index of neutrophils in peripheral blood, phagocytosis rate and phagocytic index of macrophages in enterocoelia of blank group and cefazolin group were statistically significantly lower than XYP group and XYP combined with cefazolin group. XYP combined with cefazolin can promote the recovery of cytophagocytosis of peripheral blood neutrophils and enterocoelia macrophages in mice with Staphylococcus aureus infection, and then enhance the body’s anti-infection ability and antibacterial effect (Liu et al., 2015). An animal experiment demonstrated that the actual phagocytotic rate and index of peripheral blood neutrophils in XYP group and the XYP combination of Cefazolin group were significantly higher than those of the Cefazolin group, suggested the combination of XYP and Cefazolin could enhance the therapeutic effect by improving the phagocytic function of peripheral blood neutrophils (Xiong et al., 2015). Investigations by Xiong et al. showed that XYP combined with cafezoin sodium may promote the apoptosis of neutrophils and alleviate inflammation of mice with infection by reducing the level of IL-6 and improving the level of IL-10 in plasma (Xiong et al., 2020).

Other information										
Registration	23	Registration number and name of trial registry	The clinical trial was not registered.							
Protocol	24	Where the full trial protocol can be accessed, if available	Not Applicable.							
Funding	25	Sources of funding and other support (such as supply of drugs), role of funders	Science and Technology Benefit People Project of Chengdu Science and Technology Bureau of Sichuan Province (No. : 2013-HM01-3-00004-SF)	There was no funding or other support for this study.	There was no funding or other support for this study.	There was no funding or other support for this study.	There was no funding or other support for this study.	There was no funding or other support for this study.	There was no funding or other support for this study.	There was no funding or other support for this study.

Table S2. Checklist of Items for Reporting Trials of Chinese Herbal Medicine Formulas 2.

Table. Checklist of Items for Reporting Trials of Chinese Herbal Medicine Formulas*Section/Topic	Item Number	Standard CONSORT Checklist Item	Zhou (2019)	Sun (2020)	Shuai et al. (2018)	Peng et al. (2015)	Dai (2006)	Zeng (2018)	Li and Li (2004)	Li (2017)

Title, abstract, and keywords	1a	Identification as a randomized trial in the title	Clinical study of Xiyanping injection in the treatment of children with acute suppurative tonsillitis (No TCM Pattern)							
	1b	Structured summary of trial design, methods, results, and conclusions (for specific guidance, see CONSORT for abstracts	Objective: To evaluate the clinical efficacy of Xiyanping injection in the treatment of acute suppurative tonsillitis. Methods: Researchers conducted a single-blind, randomized, controlled trials involving 120 patients with acute suppurative tonsillitis	Objective: To evaluate the clinical efficacy of Xiyanping injection in the treatment of acute suppurative tonsillitis. Methods: Researchers conducted a single-blind, randomized, controlled trials involving 359 patients with acute suppurative tonsillitis	Objective: To evaluate the clinical efficacy of Xiyanping injection in the treatment of acute suppurative tonsillitis. Methods: Researchers conducted a single-blind, randomized, controlled trials involving 108 patients with acute suppurative tonsillitis	Objective: To evaluate the clinical efficacy of Xiyanping injection in the treatment of acute suppurative tonsillitis. Methods: Researchers conducted a single-blind, randomized, controlled trials involving 100 patients with acute suppurative tonsillitis	Objective: To evaluate the clinical efficacy of Xiyanping injection in the treatment of acute suppurative tonsillitis. Methods: Researchers conducted a single-blind, randomized, controlled trials involving 68 patients with acute suppurative tonsillitis	Objective: To evaluate the clinical efficacy of Xiyanping injection in the treatment of acute suppurative tonsillitis. Methods: Researchers conducted a single-blind, randomized, controlled trials involving 86 patients with acute suppurative tonsillitis	Objective: To evaluate the clinical efficacy of Xiyanping injection in the treatment of acute suppurative tonsillitis. Methods: Researchers conducted a single-blind, randomized, controlled trials involving 126 patients with acute suppurative tonsillitis	Objective: To evaluate the clinical efficacy of Xiyanping injection in the treatment of acute suppurative tonsillitis. Methods: Researchers conducted a single-blind, randomized, controlled trials involving 108 patients with acute suppurative tonsillitis

	1c	Determination of appropriate keywords, including “Chinese herbal medicine formula” and “randomized controlled trial”	Determination of appropriate keywords, including “Xiyanping injection” and “randomized controlled trial”
Introduction Background and objectives	2a	Scientific background and explanation of rationale	Xiyanping injection (XYP), a purifying agent of TCM andrographis paniculata (Chuan Xin Lian in chinese, CXL), is a popular traditional medicinal plant. Previous studies confirmed that andrographolide was identified as safe, efficacious anti-inflammatory agent and had effects on disease of respiratory system included upper respiratory tract infections, lung injury, pneumonia, and other respiratory disease. Andrographolide can decrease the production of pro-inflammatory factors. It has antibacterial, antiviral, antipyretic and anti-inflammatory effects. Several basic researches have confirmed that andrographolide can exert anti-inflammatory effects through multiple targets (such as targets factor-α, interleukin, etc.) and multiple pathways (such as regulating silencing information regulator 1/extracellular regulatory kinase, the expression and activation of nuclear transcription factor-κB, etc.). A number of randomized controlled trial studies showed that XYP have good therapeutic effect in treatment of suppurative acute tonsillitis (SAT).
	2b	Specific objectives or hypotheses	A single-blind, randomized, controlled clinical trial has been conducted to test the efficacy and safety of Xiyanping injection in the treatment of acute suppurative tonsillitis.
Methods			

Trial design	3a	Description of trial design (such as parallel, factorial), including allocation ratio	This trial was a single-center, single-blind, randomized controlled clinical study conducted at Maternal and Child Health Service Center of Jialing District, Nanchong city, Sichuan province, China.	This trial was a single-center, single-blind, randomized controlled clinical study conducted at Jining Municipal Hospital, Shandong province, China.	This trial was a single-center, single-blind, randomized controlled clinical study conducted at Yichun People's Hospital, Jiangxi Province, China.	This trial was a single-center, single-blind, randomized controlled clinical study conducted at the Third Affiliated Hospital of Guangzhou Medical University, Guangdong Province, China.	This trial was a single-center, single-blind, randomized controlled clinical study conducted at Guankou Hospital, Jimei District, Xiamen City, Fujian Province, China.	This trial was a single-center, single-blind, randomized controlled clinical study conducted at Citic Huizhou Hospital, Huizhou City, Guangdong Province, China.	This trial was a single-center, single-blind, randomized controlled clinical study conducted at Pingyi County People's Hospital, Linyi City, Shandong Province, China.	This trial was a single-center, single-blind, randomized controlled clinical study conducted at Fuquan Maternal and Child Health Hospital, Guizhou Province, China.
	3b	Important changes to methods after trial commencement (such as eligibility criteria), with reasons	There were no important changes to methods after trial commencement.							

Participants	4a	Eligibility criteria for participants	All children had clinical symptoms related to suppurative tonsillitis and were diagnosed by examination, and none had a history of antibiotic use. Children with drug allergy, lower respiratory tract infection, severe infections, and severe organic diseases were excluded. With the consent of the children's guardians and the approval of the ethics	Inclusion criteria: meeting the relevant clinical diagnostic criteria for acute suppurative tonsillitis; All participants were informed of the study and signed the consent form. There was no history of drug allergy. Patients with good mental cognition could correctly recognize the instructions of medical staff. Exclusion criteria: allergy to the study drug; Congenital mental disorders	The specific diagnostic criteria, inclusion and exclusion criteria were not listed in this study.	Diagnostic criteria: clinical manifestations included pharyngeal pain, fever, suppuration and enlargement of tonsil, neutrophil ratio > 50%, and white blood cell count >10×10*9 / L. Inclusion criteria: ① Age 1 to 12 years old; ② Informed consent was obtained from the guardians. Exclusion criteria: ① Severe liver and kidney dysfunction; ② those who had received other treatment regimens; ③ Complicated with severe systemic infection.	The specific diagnostic criteria, inclusion and exclusion criteria were not listed in this study.	The specific diagnostic criteria, inclusion and exclusion criteria were not listed in this study.	The specific diagnostic criteria, inclusion and exclusion criteria were not listed in this study.	According to the analysis of the "Diagnosis, treatment and judgment Criteria for Children with suppurative tonsillitis" formulated by the Chinese Medical Association in 2016, all the subjects met the experimental inclusion criteria, volunteered to participate in the experimental study and actively

		committee of our hospital, 60 cases were randomly divided into two groups.	or cognitive deficits; People with impaired speech communication; Combined with immune function diseases or malignant tumors; Transfer and poor compliance.						cooperated with the analysis
4b	Settings and locations where the data were collected	The study was conducted from January to October 2018 at the Second People's Hospital of Fengrun District, located in Hebei province, China.	The study was conducted from January 2016 to January 2019 at at the Jining Municipal Hospital, located in Shandong province, China.	The study was conducted from October 2015 to December 2017 at the Yichun People's Hospital, located in Jiangxi province, China.	The study was conducted from October 2013 to October 2014 at the Third Affiliated Hospital of Guangzhou Medical University, located in Guangdong province, China.	The study was conducted from 2005 to 2006 at the Guankou Hospital, located in Fujian province, China.	The study was conducted from June 2016 to June 2017 at the Citic Huizhou Hospital, located in Guangdong province, China.	The study was conducted from June 2000 to August 2003 at the Pingyi County People's Hospital, located in Shandong province, China.	The study was conducted from April 2014 to February 2017 at Fuquan Maternal and Child Health Hospital, located in Guizhou province, China.

Interventions	5	The interventions for each group with sufficient details to allow replication, including how and when they were actually administered	Both groups received basic symptomatic treatment such as antipyretic and fluid infusion. (1) The control group was given clindamycin injection (approval number: Chinese Medicine H20054699, production enterprise: Beijing Shuanglu Pharmaceutical Co., LTD.), the dose was 25-40 mg/kg, which was added to	After admission, both groups received routine nursing and health education, and given symptomatic treatment such as basic fluid infusion. The control group was treated with cefprozil orally (Hunan Fangsheng Pharmaceutical Co., LTD., Chinese Medicine approved number H20093886, specification: 0.25g×6s)7.5mg/(kg·d), twice a day, if the	The control group was treated with clindamycin, 5mg/kg, 3 times a day for 1 week, and the infusion volume should be controlled within 1.2g for 1 hour. The patients in the treatment group were treated with clindamycin intravenously at a standard dose of 5mg/kg, 3 times a day for 1 week, and the infusion volume should be controlled within 1.2g for	The control group was only given clindamycin injection (Suzhou Tianma Pharmaceutical Group Tianji Biological Pharmaceutical Co., LTD., size: 0.6g, batch number: 1306121), 25-40mg /kg, added 500ml 0.9% sodium chloride injection, intravenous drip, once a day. Three days was a course of treatment. On the basis of the control group, the observation group was treated with Xiyanping injection (Jiangxi Qingfeng Pharmaceutical Co., LTD., specification: 5ml: 125mg, batch	Both groups were treated with penicillin injection 200 000 U/ (kg·d), 2 times intravenous drip, the treatment group was treated with Xiyanping injection 0.2-0.4 ml/ (kg·d), adding 5% glucose solution diluted into 2%-6% intravenous drip, once a day. Both groups were given supportive and symptomatic	Both groups were treated with conventional treatment, including essential nutritional elements, adequate rest, and ribavirin injection (Zhengzhou Zhuofeng Pharmaceutical Co., LTD., Chinese Medicine approval number H41023268), once a day for 5 consecutive days. On this basis, the observation	Both groups were treated with penicillin injection by intravenous drip, the dose was 200 000 ~ 300 000 U/kg·d, divided into two intravenous drops. The treatment group was treated with Xiyanping injection and 5% glucose injection, the dose was 0.2~0.4ml/kg·d. Both groups were given symptomatic and supportive treatment, and no other antibiotics were	The control group selected 54 children with tonsillitis to choose the general treatment, namely intravenous infusion of cefuroxime sodium injection, twice a day, the daily dose of 100mg/kg; The study group was treated with Xiyanping injection on the basis of the treatment group, intravenous drip, once 5-10

	0.9% sodium chloride solution 500ml, once a day by intravenous drip for 7 days. (2) The study group combined with Xiyanping injection (approval number: Chinese Medicine Z20026249, manufacturing enterprise: Jiangxi Qingfeng Pharmaceutical Co., LTD.), dosage of 5 ~ 10mg/kg, adding 5% glucose solution 250ml, once a	symptoms are serious, the dose can be increased to 8mg/ (kg·d), once a day, with 7 days as a course of treatment. The observation group was treated with Xiyanping on the basis of the control group. The methods were as following: taking Xiyanping for injection (Jiangxi Qingfeng Pharmaceutical Co., LTD., Chinese Medicine approval number	1 hour. At the same time, Xiyanping injection was given intravenously, with a standard dose of 10mg/kg, once a day for 1 week.	number: 1306121), 5-10 mg/kg, added 250 ml5% glucose injection, intravenous drip, once a day. Three days was a course of treatment.	treatment for 3 to 5 days.	group was treated with Xiyanping injection (Jiangxi Qingfeng Pharmaceutical Co., LTD., Chinese Medicine approval number Z20026249), according to the situation, with a dose of 0.2-0.1 mL/kg, and 5% glucose injection or normal saline for intravenous drip, the same treatment for 5 days.	used. The treatment time was 5-7 days.	mg/kg, once a day, the course of treatment was 7 days.

	day, continuous 7 days.	Z20026249, Specifications: 2ml：500mg×6s) was mixed with 5% glucose injection 250ml according to body weight (5-10 mg/kg), and the drip speed was adjusted at 30-40 drops /min, once a day. The course of treatment was the same as above.

Outcomes	6a	Completely defined, prespecified primary and secondary outcome measures, including how and when they were assessed	recovery rate of disease, the time of recovering normal temperature, duration of sore throat, duration of disappearance of tonsillar redness and swelling, the time of tonsil purulent discharge, hs-CRP (mg/L), TNF-α (mmol/L)	recovery rate of disease, incidence of adverse events, the time of recovering normal temperature, duration of sore throat, the time of tonsil purulent discharge, PCT (ng/ml), interleukin-6 (ng/L), TNF- α (μg/ml)	recovery rate of disease, incidence of adverse events, the time of recovering normal temperature, duration of sore throat, total time on medication, interleukin-8 (U/L), interleukin-6 (U/L)	recovery rate of disease, incidence of adverse events, the time of recovering normal temperature, duration of sore throat, duration of disappearance of tonsillar redness and swelling, duration of disappearance of tonsillar redness and swelling, interleukin-6 (ng/L), interleukin-8 (U/L), TNF-α (U/L)	recovery rate of disease,	recovery rate of disease, cough relief time, the disappearance time of pulmonary rales, the time of recovering normal temperature	Primary and secondary outcome measures were not prespecified.	recovery rate of disease, the time of recovering normal temperature, the time of tonsil purulent discharge, recovery time of white blood cells
	6b	Any changes to trial outcomes after the trial commenced, with reasons	No important changes to methods after trial commencement.							

Sample size	7a	How sample size was determined	No specific sample size determination method was recorded.
	7b	When applicable, explanation of any interim analyses and stopping guidelines	Not Applicable
Randomization Sequence generation	8a	Method used to generate the random allocation sequence	All included studies were RCTs, thirteen trials (Qiao et al., 2015; Mai, 2018; Ren, 2018; Liu, 2020; Sun, 2020; Peng et al., 2015; Li, 2017; Shi, 2018; Luo, 2016; Hu et al., 2018; Dong, 2009; Ou et al., 2017; Liu et al., 2008; Gan et al., 2020) report the specific method of randomization. One trial (Sun, 2020) used the stratified randomization. Eight trials (Qiao et al., 2015; Ren, 2018; Liu, 2020; Peng et al., 2015; Li, 2017; Shi, 2018; Luo, 2016; Ou et al., 2017) used random number tables. Three trials (Mai, 2018; Hu et al., 2018; Gan et al., 2020) used treatment modalities. Three studies were categorized according to treatment modalities. Three trials were grouped by treatment modalities. One study (Dong, 2009) described using odd or even admission number sequence.
	8b	Type of randomization; details of any restriction (such as blocking and block size)	No specific type of randomisation was recorded.

Allocation concealment mechanism	9	Mechanism used to implement the random allocation sequence (such as sequentially numbered containers), describing any steps taken to conceal the sequence until interventions were assigned	No specific mechanism used to implement the random allocation sequence was recorded.
Implementation	10	Who generated the random allocation sequence, who enrolled participants, and who assigned participants to interventions	No specific method used to generate the random allocation sequence was recorded.

Blinding	11a	If done, who was blinded after assignment to interventions (for example, participants, care providers, those assessing outcomes) and how	No specific blinding protocol was recorded.							
	11b	If relevant, description of the similarity of interventions	Not relevant.							
Statistical methods	12a	Statistical methods used to compare groups for primary and secondary outcomes	SPSS21.0 software was used to analyze the relevant data.	SPSS22.0 statistical software was used to analyze the research data. Measurement data were expressed as mean ± standard deviation (x ±s), and t test	SPSS18.0 software was used for data processing, measurement data were expressed as "x±s", t test was used for comparison between	SPSS16.0 statistical software was used to analyze the data. Measurement data were expressed as yuan ±s, and comparison between groups was analyzed using the independent sample t test. x2 test was used to compare	No specific statistical analysis method was recorded.	SPSS13.0 statistical software was used for analysis, measurement data were expressed as (x±s), and t test was used. Count data	SPSS13.0 statistical software was used for analysis, measurement data were expressed as (x±s), and t test was used. Count data were	Statistical software SPSS21.0 was used to process statistics, and Chi-square test and t test were used to evaluate the data between and within

				was used. Count data were expressed as rate (%) and χ2 test was used. P<0.05 was considered statistically significant.	groups, count data were expressed as rate (%), x2 test was used for comparison between groups. P < 0.05 was considered statistically significant.	the count data between groups. Rank sum test was used to compare the ranked data between groups. P<0.05 was considered statistically significant.		were expressed as (%) and analyzed by chi-square test. P<0.05 was considered statistically significant.	expressed as (%) and analyzed by chi-square test. P<0.05 was considered statistically significant.	groups
	12b	Methods for additional analyses, such as subgroup analyses and adjusted analyses	Subgroup and adjusted analyses were not performed.							
Results										
Participant flow (a diagram is strongly recommended)	13a	For each group, the numbers of participants who were randomly assigned,	See Table 1 for details.							

		received intended treatment, and were analyzed for the primary outcome	
	13b	For each group, losses and exclusions after randomization, together with reasons	No study reported the number of patients dropping out.
Recruitment	14a	Dates defining the periods of recruitment and follow-up	The follow-up time was the same as the treatment time, and the treatment time is shown in Table 1.
	14b	Why the trial ended or was stopped	Not applicable, none of the included studies were interrupted or stopped.

Baseline data	15	A table showing baseline demographic and clinical characteristics for each group	All the studies presented baseline data, including baseline demographic and clinical characteristics, for each group with the use of a table or text description.
Numbers analyzed	16	For each group, number of participants (denominator) included in each analysis and whether the analysis was by original assigned groups	The analysis was by original assigned groups.
Outcomes and estimation	17a	For each primary and secondary outcome, results for each	Not listed.

		group, and the estimated effect size and its precision (such as 95% confidence interval)	
	17b	For binary outcomes, presentation of both absolute and relative effect sizes is recommended	Not listed.
Ancillary analyses	18	Results of any other analyses performed, including subgroup analyses and adjusted analyses, distinguishing prespecified from exploratory	Subgroup and adjusted analyses were not performed.

Harms	19	All important harms or unintended effects in each group (for specific guidance, see CONSORT for harms [28])	See Table 1 for details.
Discussion			
Limitations	20	Trial limitations; addressing sources of potential bias; imprecision; and, if relevant, multiplicity of analyses	There were no trial limitations, addressing sources of potential bias and imprecision.

Generalizability	21	Generalizability (external validity, applicability) of the trial findings	The trial demonstrated the efficacy and safety of XYP combined with Clindamycin hydrochloride injection in the treatment of children with SAT.	The trial demonstrated the efficacy and safety of XYP combined with β-lactams in the treatment of children with SAT.	The trial demonstrated the efficacy and safety of XYP combined with Clindamycin hydrochloride injection in the treatment of children with SAT.	The trial demonstrated the efficacy and safety of XYP combined with Clindamycin hydrochloride injection in the treatment of children with SAT.	The trial demonstrated the efficacy and safety of XYP combined with β-lactams in the treatment of children with SAT.	The trial demonstrated the efficacy and safety of XYP combined with Ribavirin in the treatment of children with SAT.	The trial demonstrated the efficacy and safety of XYP combined with β-lactams in the treatment of children with SAT.	The trial demonstrated the efficacy and safety of XYP combined with β-lactams in the treatment of children with SAT.

Interpretation	22	Interpretation consistent with results, balancing benefits and harms, and considering other relevant evidence	Suppurative acute tonsillitis in children is knowned as "Lan Ru E" in chinese. The onset of SAT is rapid, and it is mostly caused by the invasion of tonsil by fire and heat evil in theory of traditional Chinese medicine (TCM). TCM andrographis paniculata is the component of XYP. The efficacy of Chuan Xin Lian (CXL) is to clear heat and detoxify, cool blood, and detumescence. CXL can be used for the treatment of cold and fever, sore throat, mouth and tongue sores, cough, diarrhea and dysentery, hot and astringent pain, carcarols and sores, snake bites. Along with clinical use, the activity and mechanism of XYP has also been evaluated in experimental model systems. Investigations by Fan et al. showed that XYP, a new candidate of antiviral drug for DENV infection, can inactivate DENV2 and inhibit the entry of DENV2 into cells. XYP can significantly inhibit the replication of DENV2 in a dose-dependent manner and reduce the viral load in organs and sera, and improve the survival rate of experimental mice. The antivirus activity of XYP is superior to ribavirin injection in the early and late stage of virus infection (Fan et al., 2020). In terms of safety, investigations by Yu et al. showed that compared with the control group, drugs in the group of XYP for injection 400mg/kg, 200mg/kg and 100mg/kg, XYP had no significant effect on the central nervous systems, cardiovascular and respiratory systems of experimental animals (Yu et al., 2009). Investigations by Liu et al. showed that actual phagocytosis rate and phagocytic index of neutrophils in peripheral blood, phagocytosis rate and phagocytic index of macrophages in enterocoelia of blank group and cefazolin group were statistically significantly lower than XYP group and XYP combined with cefazolin group. XYP combined with cefazolin can promote the recovery of cytophagocytosis of peripheral blood neutrophils and enterocoelia macrophages in mice with Staphylococcus aureus infection, and then enhance the body's anti-infection ability and antibacterial effect (Liu et al., 2015). An animal experiment demonstrated that the actual phagocytotic rate and index of peripheral blood neutrophils in XYP group and the XYP combination of Cefazolin group were significantly higher than those of the Cefazolin group, suggested the combination of XYP and Cefazolin could enhance the therapeutic effect by improving the phagocytic function of peripheral blood neutrophils (Xiong et al., 2015). Investigations by Xiong et al. showed that XYP combined with cafezoin sodium may promote the apoptosis of neutrophils and alleviate inflammation of mice with infection by reducing the level of IL-6 and improving the level of IL-10 in plasma (Xiong et al., 2020).
Other information			
Registration	23	Registration number and name of trial registry	The clinical trial was not registered.
Protocol	24	Where the full trial protocol can be	Not Applicable.

		accessed, if available								
Funding	25	Sources of funding and other support (such as supply of drugs), role of funders	There was no funding or other support for this study.	There was no funding or other support for this study.	There was no funding or other support for this study.	Science and Technology Program of Guangdong Province, No.2010A030400010	There was no funding or other support for this study.	There was no funding or other support for this study.	There was no funding or other support for this study.	There was no funding or other support for this study.

Table S3. Checklist of Items for Reporting Trials of Chinese Herbal Medicine Formulas 3.

Table. Checklist of Items for Reporting Trials of Chinese Herbal Medicine Formulas*Section/Topic	Item Number	Standard CONSORT Checklist Item	Jiang and Yang (2012)	Pan et al. (2014)	Wang (2015)	Shi (2018)	Luo (2016)	Hu et al. (2018)	Dong (2009)	He (2013)
Title, abstract, and keywords	1a	Identification as a randomized trial in the title	Clinical study of Xiyanping injection in the treatment of children with acute suppurative tonsillitis (No TCM Pattern)							
	1b	Structured summary of	Objective: To evaluate the	Objective: To evaluate the	Objective: To evaluate the	Objective: To evaluate the	Objective: To evaluate	Objective: To evaluate the	Objective: To evaluate the	Objective: To evaluate

	trial design, methods, results, and conclusions (for specific guidance, see CONSORT for abstracts	clinical efficacy of Xiyanping injection in the treatment of acute suppurative tonsillitis. Methods: Researchers conducted a single-blind, randomized, controlled trials involving 76 patients with acute suppurative tonsillitis	clinical efficacy of Xiyanping injection in the treatment of acute suppurative tonsillitis. Methods: Researchers conducted a single-blind, randomized, controlled trials involving 120 patients with acute suppurative tonsillitis	clinical efficacy of Xiyanping injection in the treatment of acute suppurative tonsillitis. Methods: Researchers conducted a single-blind, randomized, controlled trials involving 78 patients with acute suppurative tonsillitis	clinical efficacy of Xiyanping injection in the treatment of acute suppurative tonsillitis. Methods: Researchers conducted a single-blind, randomized, controlled trials involving 900 patients with acute suppurative tonsillitis	the clinical efficacy of Xiyanping injection in the treatment of acute suppurative tonsillitis. Methods: Researchers conducted a single-blind, randomized, controlled trials involving 98 patients with acute suppurative tonsillitis	clinical efficacy of Xiyanping injection in the treatment of acute suppurative tonsillitis. Methods: Researchers conducted a single-blind, randomized, controlled trials involving 108 patients with acute suppurative tonsillitis	clinical efficacy of Xiyanping injection in the treatment of acute suppurative tonsillitis. Methods: Researchers conducted a single-blind, randomized, controlled trials involving 82 patients with acute suppurative tonsillitis	the clinical efficacy of Xiyanping injection in the treatment of acute suppurative tonsillitis. Methods: Researchers conducted a single-blind, randomized, controlled trials involving 114 patients with acute suppurative tonsillitis

	1c	Determination of appropriate keywords, including “Chinese herbal medicine formula” and “randomized controlled trial”	Determination of appropriate keywords, including “Xiyanping injection” and “randomized controlled trial”
Introduction Background and objectives	2a	Scientific background and explanation of rationale	Xiyanping injection (XYP), a purifying agent of TCM andrographis paniculata (Chuan Xin Lian in chinese, CXL), is a popular traditional medicinal plant. Previous studies confirmed that andrographolide was identified as safe, efficacious anti-inflammatory agent and had effects on disease of respiratory system included upper respiratory tract infections, lung injury, pneumonia, and other respiratory disease. Andrographolide can decrease the production of pro-inflammatory factors. It has antibacterial, antiviral, antipyretic and anti-inflammatory effects. Several basic researches have confirmed that andrographolide can exert anti-inflammatory effects through multiple targets (such as targets factor-α, interleukin, etc.) and multiple pathways (such as regulating silencing information regulator 1/extracellular regulatory kinase, the expression and activation of nuclear transcription factor-κB, etc.). A number of randomized controlled trial studies showed that XYP have good therapeutic effect in treatment of suppurative acute tonsillitis (SAT).
	2b	Specific objectives or hypotheses	A single-blind, randomized, controlled clinical trial has been conducted to test the efficacy and safety of Xiyanping injection in the treatment of acute suppurative tonsillitis.
Methods			

Trial design	3a	Description of trial design (such as parallel, factorial), including allocation ratio	This trial was a single-center, single-blind, randomized controlled clinical study conducted at Chengdu Hospital of Integrated Traditional Chinese and Western Medicine, Sichuan Province, China.	This trial was a single-center, single-blind, randomized controlled clinical study conducted at Affiliated Hospital of Hebei Engineering University, Hebei Province, China.	This trial was a single-center, single-blind, randomized controlled clinical study conducted at Xuchang Central Hospital, Henan Province, China.	This trial was a single-center, single-blind, randomized controlled clinical study conducted at People's Hospital of Linxia County, Gansu Province, China.	This trial was a single-center, single-blind, randomized controlled clinical study conducted at Xiping County People's Hospital, Henan Province, China.	This trial was a single-center, single-blind, randomized controlled clinical study conducted at Dangyang People's Hospital, Yichang City, Hubei Province, China.	This trial was a single-center, single-blind, randomized controlled clinical study conducted at The Fourth Affiliated Hospital, Guangxi Medical University, China.	This trial was a single-center, single-blind, randomized controlled clinical study conducted at Qixian People's Hospital, Kaifeng city, Henan province, China.
	3b	Important changes to methods after trial commencement (such as eligibility criteria), with reasons	There were no important changes to methods after trial commencement.							

Participants	4a	Eligibility criteria for participants	According to the "TCM diagnostic and therapeutic Criteria" issued by the Department of Medical Administration of the State Administration of Traditional Chinese Medicine in June 1994: cure: normal body temperature, normal blood picture, disappearance of pharyngeal symptoms, tonsil no congestion, no pus point; He	According to the diagnostic criteria of acute suppurative tonsillitis in Practical Otolaryngology published by Huang Xuanzhao et al. J: the onset time was not more than 72 hours, and the clinical manifestations were fever, sore throat, dysphagia, cough and so on. Local throat examination showed that the tonsils were hyperemic, bright red or dark red, II-III degree diffuse swelling,	The specific diagnostic criteria, inclusion and exclusion criteria were not listed in this study.	The specific diagnostic criteria, inclusion and exclusion criteria were not listed in this study.	The specific diagnostic criteria, inclusion and exclusion criteria were not listed in this study.	Inclusion criteria: all patients met the diagnostic criteria of suppuration tonsillitis in "Applied Pediatric Otolaryngology" , with symptoms such as fever, sore throat, tonsil enlargement, white blood cell count > 10×109/L, neutrophils 50%, no history of antibiotic use, and their family members volunteered to participate in the study and signed the consent form.	The specific diagnostic criteria, inclusion and exclusion criteria were not listed in this study.	The specific diagnostic criteria, inclusion and exclusion criteria were not listed in this study.

	still had low fever, blood count was close to normal, pharyngeal symptoms were relieved, and tonsillar pus spots were eliminated. Uncured: there was no significant improvement in symptoms and signs.	with purulent secretion on the surface. Blood routine examination showed white blood cell count >10.0×10* 9 / L and neutrophil ratio > 70%.	

	4b	Settings and locations where the data were collected	The study was conducted from June 2010 to June 2011 at Chengdu Hospital of Integrated Traditional Chinese and Western Medicine, located in Sichuan province, China.	The study was conducted from March 2012 to August 2013 at Affiliated Hospital of Hebei Engineering University, located in Hebei province, China.	The study was conducted from April 2014 to April 2015 at Xuchang Central Hospital, located in Henan province, China.	The study was conducted from March 2014 to October 2017 at People's Hospital of Linxia County, located in Gansu province, China.	The study was conducted from May 2014 to January 2016 at Xiping County People's Hospital, located in Henan province, China.	The study was conducted from December 2016 to December 2017 at Dangyang People's Hospital, located in Hubei province, China.	The study was conducted from January to October 2008 at the Fourth Affiliated Hospital, located in Guangxi province, China.	The study was conducted from January 2012 to January 2013 at the Qixian People's Hospital, located in Henan province, China.
Interventions	5	The interventions for each group with sufficient details to allow replication, including how and when they were actually administered	The control group was given routine fluid replacement and adequate antibiotics (penicillin or cephalosporin) treatment. On the basis of the	Control group: azithromycin injection, according to the standard of 10mg·kg ~ ·d dissolved in 500ml 5% glucose injection, after completely dissolved,	The treatment group was treated with Xiyanping (Jiangxi Qingfeng Pharmaceutical Co., LTD., Chinese Medicine approved name	Conventional western medicine treatment group: patients in the conventional western medicine treatment group were treated with conventional western medicine, mainly including intravenous drip of	The children in the observation group were treated with clindamycin combined with Xiyanping, 5 ~ 10mg/kg Xiyanping (Jiangxi	Both groups were treated with antipyretic, fluid infusion and other basic symptomatic treatment. The control group was treated with clindamycin injection	Both groups were given clindamycin 25-40 mg/kg·d, added into normal saline 500ml intravenous drip, the treatment group was	The children in the treatment group were given 100mL normal saline and 5~8mg/kg clindamycin for injection produced by Hainan

	treatment of the control group, the treatment group was treated with Xiyanping injection (produced by Jiangxi Qingfeng Pharmaceutical Co., LTD., approval number: national approval number Z20026249)250mg added to normal saline 250ml by intravenous drip, once a day. Both groups took 5	intravenous drip. Treatment group: on the basis of the control group, Xiyanping injection 5-10mg/kg·d added 5% glucose injection 250ml intravenous drip. Five days was a course of treatment.	z20026249) combined with clindamycin (Yichang Renfu Pharmaceutical Co., LTD., Chinese Medicine approved name H20058607). After admission, 100ml of normal saline and 5~8mg/kg clindamycin were given by intravenous drip,3 times a day, 7 days as a course of treatment. The speed of intravenous infusion should	β-lactam antibiotics, in addition to diet control, pay attention to rest and other conventional treatment. Xiyanping injection treatment group: Xiyanping injection treatment group was treated with Xiyanping injection on the basis of conventional western medicine treatment, in which the conventional western medicine treatment method was the same as the conventional western medicine treatment group. The dosage of Xiyanping injection (Jiangxi Qingfeng Pharmaceutical Co., LTD., Chinese	Qingfeng Pharmaceutical Co., LTD., Chinese medicine approval number: Z20026249) mixed with 100ml 5% glucose injection, intravenous drip, once a day; 5 ~ 8mg/kg clindamycin (Yichang Renfu Pharmaceutical Co., LTD., Chinese Medicine approval name: H20058607)	(Chongqing Laimei Pharmaceutical Co., LTD., Chinese Medicine approved number H20020152, 4ml: 0.5g). The treatment: clindamycin 25-40mg /kg, added to 0.9% sodium chloride solution 500ml, intravenous drip, once a day, continuous treatment for 1 week. On the basis of the control group, the observation group was treated with	additionally given Xiyanping injection (Jiangxi Qingfeng Pharmaceutical Co., LTD.). Approval number: Z20026249)5-10mg/kg·d, adding 5% glucose injection 250ml drip. Both groups were given symptomatic and supportive treatment, 5 to 7 days as a course of treatment.	Shuangcheng Pharmaceutical Co., LTD. (Chinese Medicine approval number H20030869), 3 times a day by intravenous drip. The speed of infusion should be strictly controlled, and the amount of infusion should not exceed 1.2g within 1 hour. At the same time, 5% glucose 100mL was added to

	days as a course of treatment	be strictly controlled, and the amount of drug infused within 1 hour should be less than 1.2g. At the same time, 5~10mg/kg Xiyanping was given intravenously by 5% glucose solution 100m once a day. In addition, symptomatic treatments such as nutritional support, antiemetic and physical cooling should be carried out according to the actual	Medicine approval number Z20026249) is 0.2-0.4 ml/kg·d, and attention should be paid to add the injection into 50ml glucose solution. The treatment method is intravenous drip.	was mixed with 100ml of normal saline, intravenous infusion, 3 times a day, strictly control the infusion speed, to ensure that the amount of drug input within 1h within 1.2g. The children in the control group were only treated with clindamycin, and the use method and dose of clindamycin were the same as those in the	Xiyanping injection (Jiangxi Qingfeng Pharmaceutical Co., LTD., Chinese medicine Z20026249, 2ml：50mg×6 / box) : Xiyanping 5 ~ 10mg/kg, added to 5% glucose solution 250ml, intravenous drip, once a day, continuous treatment for 1 week.	Jiangxi Qingfeng Pharmaceutical Co., LTD. Xiyanping injection (Chinese medicine approval name Z20026249) 5-10 mg/kg, intravenous drip once a day. In addition, symptomatic treatment was given according to the patient's symptoms, including physical cooling, antiemetic, nutritional

	situation of patients. ② Only the control group was treated with clindamycin, and the treatment method was the same as the treatment group	observation group. Continuous treatment for 7 days was a course of treatment, and the children in the two groups were treated for 1 course of treatment. During the treatment, the changes of clinical data of the children were closely monitored, and the corresponding antiemetic, nutritional support, physical cooling and	support and other treatments. Seven days was a course of treatment. The control group was treated with clindamycin alone, and the usage and dosage were the same as the treatment group. Other treatments were the same as the treatment group.

other auxiliary treatment measures were given.

Outcomes	6a	Completely defined, prespecified primary and secondary outcome measures, including how and when they were assessed	recovery rate of disease, the disappearance rate of tonsillar redness and swelling, the time of recovering normal temperature, duration of sore throat, the recovery rate of white blood cell count	recovery rate of disease, the disappearance rate of tonsillar redness and swelling, the recovery rate of white blood cell count, cough relief timeh, duration of sore throat	recovery rate of disease, interleukin-8 (U/L), interleukin-6 (U/L), TNF-α (U/L),	recovery rate of disease, the disappearance rate of tonsillar redness and swelling, the time of recovering normal temperature, cough relief timeh, duration of sore throat	recovery rate of disease, interleukin-8, interleukin-6, TNF-α	recovery rate of disease, the time of recovering normal temperature, the time of recovering normal temperature, the time of tonsil purulent discharge, length of hospitliztion, hs-CRP (mg/L), TNF-α (mmol/L), interleukin-6 (mmol/L), interleukin-8 (mmol/L)	recovery rate of disease,	recovery rate of disease, incidence of adverse events,
	6b	Any changes to trial outcomes after the trial commenced, with reasons	No important changes to methods after trial commencement.							

Sample size	7a	How sample size was determined	No specific sample size determination method was recorded.
	7b	When applicable, explanation of any interim analyses and stopping guidelines	Not Applicable
Randomization Sequence generation	8a	Method used to generate the random allocation sequence	All included studies were RCTs, thirteen trials (Qiao et al., 2015; Mai, 2018; Ren, 2018; Liu, 2020; Sun, 2020; Peng et al., 2015; Li, 2017; Shi, 2018; Luo, 2016; Hu et al., 2018; Dong, 2009; Ou et al., 2017; Liu et al., 2008; Gan et al., 2020) report the specific method of randomization. One trial (Sun, 2020) used the stratified randomization. Eight trials (Qiao et al., 2015; Ren, 2018; Liu, 2020; Peng et al., 2015; Li, 2017; Shi, 2018; Luo, 2016; Ou et al., 2017) used random number tables. Three trials (Mai, 2018; Hu et al., 2018; Gan et al., 2020) used treatment modalities. Three studies were categorized according to treatment modalities. Three trials were grouped by treatment modalities. One study (Dong, 2009) described using odd or even admission number sequence.
	8b	Type of randomization; details of any restriction (such as blocking and block size)	No specific type of randomisation was recorded.

Allocation concealment mechanism	9	Mechanism used to implement the random allocation sequence (such as sequentially numbered containers), describing any steps taken to conceal the sequence until interventions were assigned	No specific mechanism used to implement the random allocation sequence was recorded.
Implementation	10	Who generated the random allocation sequence, who enrolled participants, and who assigned participants to interventions	No specific method used to generate the random allocation sequence was recorded.

Blinding	11a	If done, who was blinded after assignment to interventions (for example, participants, care providers, those assessing outcomes) and how	No specific blinding protocol was recorded.							
	11b	If relevant, description of the similarity of interventions	Not relevant.							
Statistical methods	12a	Statistical methods used to compare groups for primary and secondary outcomes	SPSS11.0 software was used to analyze the data. Measurement data were expressed as mean ± SD, and comparison between groups	SPSS16.0 statistical software was used, measurement data were expressed as ±s, t test was used, count data were analyzed by x test, P<0.05 was considered	SPSS18.0 statistical software was used for data processing and analysis. Quantitative data were represented by (+), and the comparison	The statistical software was SPSS17.0.	No specific statistical analysis method was recorded.	SPSS21.0 software was used for data analysis, measurement data were expressed as "x±s", and t test was used for comparison between groups.	SPSS software was used for statistical analysis. The rank sum test was used for ranked data, and P<0.05 was considered statistically significant.	The data of this group were analyzed by statistical software SPSS13.5, and the count data were tested by χ2 test. P<0.01 was considered

			was analyzed using the t test. Count data were expressed as rate (%), and χ2 test was used for comparison between groups. P < 0.05 was considered statistically significant.	statistically significant.	between groups was performed by test. Qualitative data were compared by test, and P<0.05 was considered statistically significant	The enumeration data were expressed by the number of cases (n), and the comparison of the rate (%) of enumeration data between groups was analyzed by x2 test. P < 0.05 was considered statistically significant.	statistically significant
	12b	Methods for additional analyses, such as subgroup analyses and adjusted analyses	Subgroup and adjusted analyses were not performed.				
Results							

Participant flow (a diagram is strongly recommended)	13a	For each group, the numbers of participants who were randomly assigned, received intended treatment, and were analyzed for the primary outcome	See Table 1 for details.
	13b	For each group, losses and exclusions after randomization, together with reasons	No study reported the number of patients dropping out.
Recruitment	14a	Dates defining the periods of recruitment and follow-up	The follow-up time was the same as the treatment time, and the treatment time is shown in Table 1.

	14b	Why the trial ended or was stopped	Not applicable, none of the included studies were interrupted or stopped.
Baseline data	15	A table showing baseline demographic and clinical characteristics for each group	All the studies presented baseline data, including baseline demographic and clinical characteristics, for each group with the use of a table or text description.
Numbers analyzed	16	For each group, number of participants (denominator) included in each analysis and whether the analysis was by original assigned groups	The analysis was by original assigned groups.

Outcomes and estimation	17a	For each primary and secondary outcome, results for each group, and the estimated effect size and its precision (such as 95% confidence interval)	Not listed.
	17b	For binary outcomes, presentation of both absolute and relative effect sizes is recommended	Not listed.
Ancillary analyses	18	Results of any other analyses performed, including subgroup analyses and adjusted	Subgroup and adjusted analyses were not performed.

		analyses, distinguishing prespecified from exploratory	
Harms	19	All important harms or unintended effects in each group (for specific guidance, see CONSORT for harms [28])	See Table 1 for details.
Discussion			
Limitations	20	Trial limitations; addressing sources of potential bias; imprecision; and, if relevant, multiplicity of analyses	There were no trial limitations, addressing sources of potential bias and imprecision.

Generalizability	21	Generalizability (external validity, applicability) of the trial findings	The trial demonstrated the efficacy and safety of XYP combined with β-lactams in the treatment of children with SAT.	The trial demonstrated the efficacy and safety of XYP combined with Azithromycin in the treatment of children with SAT.	The trial demonstrated the efficacy and safety of XYP combined with Clindamycin hydrochloride injection in the treatment of children with SAT.	The trial demonstrated the efficacy and safety of XYP combined with β-lactams in the treatment of children with SAT.	The trial demonstrated the efficacy and safety of XYP combined with Clindamycin hydrochloride injection in the treatment of children with SAT.	The trial demonstrated the efficacy and safety of XYP combined with Clindamycin hydrochloride injection in the treatment of children with SAT.	The trial demonstrated the efficacy and safety of XYP combined with Clindamycin hydrochloride injection in the treatment of children with SAT.	The trial demonstrated the efficacy and safety of XYP combined with Clindamycin hydrochloride injection in the treatment of children with SAT.

Interpretation	22	Interpretation consistent with results, balancing benefits and harms, and considering other relevant evidence	Suppurative acute tonsillitis in children is knowned as "Lan Ru E" in chinese. The onset of SAT is rapid, and it is mostly caused by the invasion of tonsil by fire and heat evil in theory of traditional Chinese medicine (TCM). TCM andrographis paniculata is the component of XYP. The efficacy of Chuan Xin Lian (CXL) is to clear heat and detoxify, cool blood, and detumescence. CXL can be used for the treatment of cold and fever, sore throat, mouth and tongue sores, cough, diarrhea and dysentery, hot and astringent pain, carcarols and sores, snake bites. Along with clinical use, the activity and mechanism of XYP has also been evaluated in experimental model systems. Investigations by Fan et al. showed that XYP, a new candidate of antiviral drug for DENV infection, can inactivate DENV2 and inhibit the entry of DENV2 into cells. XYP can significantly inhibit the replication of DENV2 in a dose-dependent manner and reduce the viral load in organs and sera, and improve the survival rate of experimental mice. The antivirus activity of XYP is superior to ribavirin injection in the early and late stage of virus infection (Fan et al., 2020). In terms of safety, investigations by Yu et al. showed that compared with the control group, drugs in the group of XYP for injection 400mg/kg, 200mg/kg and 100mg/kg, XYP had no significant effect on the central nervous systems, cardiovascular and respiratory systems of experimental animals (Yu et al., 2009). Investigations by Liu et al. showed that actual phagocytosis rate and phagocytic index of neutrophils in peripheral blood, phagocytosis rate and phagocytic index of macrophages in enterocoelia of blank group and cefazolin group were statistically significantly lower than XYP group and XYP combined with cefazolin group. XYP combined with cefazolin can promote the recovery of cytophagocytosis of peripheral blood neutrophils and enterocoelia macrophages in mice with Staphylococcus aureus infection, and then enhance the body's anti-infection ability and antibacterial effect (Liu et al., 2015). An animal experiment demonstrated that the actual phagocytotic rate and index of peripheral blood neutrophils in XYP group and the XYP combination of Cefazolin group were significantly higher than those of the Cefazolin group, suggested the combination of XYP and Cefazolin could enhance the therapeutic effect by improving the phagocytic function of peripheral blood neutrophils (Xiong et al., 2015). Investigations by Xiong et al. showed that XYP combined with cafezoin sodium may promote the apoptosis of neutrophils and alleviate inflammation of mice with infection by reducing the level of IL-6 and improving the level of IL-10 in plasma (Xiong et al., 2020).
Other information			
Registration	23	Registration number and name of trial registry	The clinical trial was not registered.
Protocol	24	Where the full trial protocol can be	Not Applicable.

		accessed, if available	
Funding	25	Sources of funding and other support (such as supply of drugs), role of funders	There was no funding or other support for this study.

Table S4. Checklist of Items for Reporting Trials of Chinese Herbal Medicine Formulas 4.

Table. Checklist of Items for Reporting Trials of Chinese Herbal Medicine Formulas*Section/Topic	Item Number	Standard CONSORT Checklist Item	Zou (2010)	Gu (2019)	Long and Cai (2014)	Gong (2020)	Ou et al. (2017)	Liu et al. (2008)	Zhou (2018)	Gan et al. (2020)
Title, abstract, and keywords	1a	Identification as a randomized trial in the title	Clinical study of Xiyanping injection in the treatment of children with acute suppurative tonsillitis (No TCM Pattern)							
	1b	Structured summary of trial design, methods,	Objective: To evaluate the clinical efficacy of	Objective: To evaluate the clinical efficacy of	Objective: To evaluate the clinical efficacy of	Objective: To evaluate the clinical efficacy of Xiyanping	Objective: To evaluate the clinical efficacy of Xiyanping	Objective: To evaluate the clinical efficacy of	Objective: To evaluate the clinical efficacy of	Objective: To evaluate the clinical efficacy of

	results, and conclusions (for specific guidance, see CONSORT for abstracts	Xiyanping injection in the treatment of acute suppurative tonsillitis. Methods: Researchers conducted a single-blind, randomized, controlled trials involving 60 patients with acute suppurative tonsillitis	Xiyanping injection in the treatment of acute suppurative tonsillitis. Methods: Researchers conducted a single-blind, randomized, controlled trials involving 90 patients with acute suppurative tonsillitis	Xiyanping injection in the treatment of acute suppurative tonsillitis. Methods: Researchers conducted a single-blind, randomized, controlled trials involving 60 patients with acute suppurative tonsillitis	injection in the treatment of acute suppurative tonsillitis. Methods: Researchers conducted a single-blind, randomized, controlled trials involving 124 patients with acute suppurative tonsillitis	injection in the treatment of acute suppurative tonsillitis. Methods: Researchers conducted a single-blind, randomized, controlled trials involving 200 patients with acute suppurative tonsillitis	Xiyanping injection in the treatment of acute suppurative tonsillitis. Methods: Researchers conducted a single-blind, randomized, controlled trials involving 69 patients with acute suppurative tonsillitis	Xiyanping injection in the treatment of acute suppurative tonsillitis. Methods: Researchers conducted a single-blind, randomized, controlled trials involving 90 patients with acute suppurative tonsillitis	Xiyanping injection in the treatment of acute suppurative tonsillitis. Methods: Researchers conducted a single-blind, randomized, controlled trials involving 60 patients with acute suppurative tonsillitis
1c	Determination of appropriate keywords, including “Chinese herbal medicine formula” and “randomized	Determination of appropriate keywords, including “Xiyanping injection” and “randomized controlled trial”							

		controlled trial"	
Introduction Background and objectives	2a	Scientific background and explanation of rationale	Xiyanping injection (XYP), a purifying agent of TCM andrographis paniculata (Chuan Xin Lian in chinese, CXL), is a popular traditional medicinal plant. Previous studies confirmed that andrographolide was identified as safe, efficacious anti-inflammatory agent and had effects on disease of respiratory system included upper respiratory tract infections, lung injury, pneumonia, and other respiratory disease. Andrographolide can decrease the production of pro-inflammatory factors. It has antibacterial, antiviral, antipyretic and anti-inflammatory effects. Several basic researches have confirmed that andrographolide can exert anti-inflammatory effects through multiple targets (such as targets factor-α, interleukin, etc.) and multiple pathways (such as regulating silencing information regulator 1/extracellular regulatory kinase, the expression and activation of nuclear transcription factor-κB, etc.). A number of randomized controlled trial studies showed that XYP have good therapeutic effect in treatment of suppurative acute tonsillitis (SAT).
	2b	Specific objectives or hypotheses	A single-blind, randomized, controlled clinical trial has been conducted to test the efficacy and safety of Xiyanping injection in the treatment of acute suppurative tonsillitis.
Methods			

Trial design	3a	Description of trial design (such as parallel, factorial), including allocation ratio	This trial was a single-center, single-blind, randomized controlled clinical study conducted at Sanfutan Town Health Center, Xiantao city, Hubei province, China. 本研究 2007~2008 年	This trial was a single-center, single-blind, randomized controlled clinical study conducted at Shulan People's Hospital, Jilin Province, China.	This trial was a single-center, single-blind, randomized controlled clinical study conducted at Qiongshan District Maternal and Child Health Hospital of Haikou City, Hainan Province, China.	This trial was a single-center, single-blind, randomized controlled clinical study conducted at Jinta County People's Hospital, Jiuquan City, Gansu Province, China.	This trial was a single-center, single-blind, randomized controlled clinical study conducted at the First Affiliated Hospital of Guangzhou University of Traditional Chinese Medicine, Guangdong Province, China.	This trial was a single-center, single-blind, randomized controlled clinical study conducted at the Shenzhen Maternal and Child Health Hospital Affiliated to Southern Medical University, Guangdong Province, China.	This trial was a single-center, single-blind, randomized controlled clinical study conducted at the Shandong Energy Xinwen Mining Group Central Hospital, Shandong Province, China.	This trial was a single-center, single-blind, randomized controlled clinical study conducted at the Chongqing Banan District Hospital of Traditional Chinese Medicine, China.
	3b	Important changes to methods after trial commencement (such as eligibility criteria), with	There were no important changes to methods after trial commencement.							

		reasons								
Participants	4a	Eligibility criteria for participants	The specific diagnostic criteria, inclusion and exclusion criteria were not listed in this study.	The specific diagnostic criteria, inclusion and exclusion criteria were not listed in this study.	)The specific diagnostic criteria, inclusion and exclusion criteria were not listed in this study.	Inclusion criteria: ① There were symptoms such as sore throat, tonsil enlargement, fever, and some children had cough; ② age ≥1 year old [2]; ③ no use of other antibiotics in the past 30 days; ④ Informed consent was obtained from patients and their families. 5) good compliance. Exclusion criteria: ① congenital heart disease; ②	The diagnostic criteria of acute suppurative tonsillitis in children were as following: fever, sore throat, dysphagia, with or without cough, enlarged tonsils, pus spots or pus, expectoration at rale, elevated white blood cells and neutrophils, and lower respiratory tract infection was excluded. Inclusion criteria: ① Meeting the above diagnostic criteria of acute suppurative	The specific diagnostic criteria, inclusion and exclusion criteria were not listed in this study.	The specific diagnostic criteria, inclusion and exclusion criteria were not listed in this study.	The specific diagnostic criteria, inclusion and exclusion criteria were not listed in this study.

Severe immune deficiency diseases; ③ Contraindications of drug use.	tonsillitis/FJL; ②i age ≤ age ≤14 years old; ③ The time from fever to treatment was 2 to 3 days, and no other antibiotics were used before treatment. ④ the parents of the children can understand and sign the informed consent. Exclusion criteria: (1) patients with severe immunodeficiency or chronic diseases, severe malnutrition; ② accompanied by other serious primary diseases of the heart, liver, kidney and hematopoietic system; ③ with severe complications such as sepsis; ④

						patients with a history of penicillin anaphylactic shock or other severe allergies; ⑤ patients with Xiyanping allergy history; ⑥ Participating in other drug clinical observers.			
4b	Settings and locations where the data were collected	The study was conducted from January 2007 to 2008 at the Sanfutan Town Health Centerl, located in Hubei province, China.	The study was conducted from August 2017 to August 2018 at the Shulan People's Hospital, located in Jilin province, China.	The study was conducted from June 2012 to June 2013 at the Qiongshan District Maternal and Child Health Hospital of Haikou City, located in Hainan province, China.	The study was conducted from June 2012 to June 2013 at the Jinta County People's Hospital, located in Gansu province, China.	The study was conducted from January 2012 to November 2016 at the First Affiliated Hospital of Guangzhou University of Traditional Chinese Medicine, located in Guangdong province, China.	The study was conducted from 2007 at the Shenzhen Maternal and Child Health Hospital Affiliated to Southern Medical University, located in Guangdong province, China.	The study was conducted from April 2016 to April 2017 at the Shandong Energy Xinwen Mining Group Central Hospital, located in Shandong province, China.	The study was conducted from February 2019 to February 2020 at the the Chongqing Banan District Hospital of Traditional Chinese Medicine, located in Chongqing, China.

Interventions	5	The interventions for each group with sufficient details to allow replication, including how and when they were actually administered	Both groups were given symptomatic and supportive treatment and penicillin injection 200 000 U/ (kg·d), divided into two intravenous drops. In the treatment group, Xiyanping injection 0.2-0.4mL/ (kg·d) was added to 5% glucose solution diluted into 2%-6% solution by intravenous drip, once a day. The course	Control group: the control group was treated with intravenous infusion of clindamycin (produced by Beijing Sihuan Kebao Pharmaceutical Co., LTD., the approval number of Chinese Medicine approval number H20050301), 5mg/kg each time, 3 times a day, continuous treatment for 7 days, the drug input dose should not	The children in the control group were given intravenous infusion of mezlocillin sulbactam sodium 75mg/ (kg· time),2 times/day, added to 100 ml 5% glucose injection intravenous drip; The observation group was combined with Xiyanping injection on the basis of the control group, the usage was 0.2ml/ (kg· time), 1	Both groups were given diet control, more water and more rest. ① The control group was treated with conventional therapy, that is, ribavirin injection was selected, and normal saline or glucose solution was used to dilute ribavirin before clinical use, intravenous drip, the dosage was 10-15 mg/kg, the drip rate was controlled, and the treatment was 3-7 days according to the condition of the children. ② The observation group	The control group was treated with conventional western medicine. Including: intravenous infusion of β-lactam antibiotics, with reasonable diet, routine nursing, antipyretic, fluid replacement and other symptomatic and supportive treatment. The treatment duration was 5 days. The treatment group was treated with Xiyanping injection on the basis of conventional western medicine treatment in the control group. Usage: Xi phlogistic flat injection (andrographis	Both groups were treated with penicillin injection intravenous drip, the dose was 200 000 U/ (kg·d), divided into two intravenous drip, the treatment group was treated with Xiyanping injection (Jiangxi Qingfeng Pharmaceutical Co., LTD., specifications 50mg, 2ml)5-10mg/ (kg·d), 5-10mg/kg, the treatment	The control group was treated with antibiotics, 1 million to 3 million units of penicillin were given by intravenous drip,2 times /d,5 days as a course of treatment. On the basis of the control group, the observation group was treated with Xiyanping injection (Jiangxi Qingfeng Pharmaceutical Co., LTD., Chinese Medicine	Conventional treatment: normal saline and ribavirin injection were administered intravenously. The dose is 10-15mg/kg, and treatment is continued for 3 to 7 days, depending on the patient's condition; Xiyanping Injection: Normal saline and Xiyanping injection were given intravenously, 2-4ml each time. Treatment was continued for 3

	of treatment was 3-5 days	exceed 1.2g/h. Study group: the study group was treated with clindamycin combined with Xiyanping injection, and the clindamycin treatment was the same as the control group. At the same time, Xiyanping injection (manufactured by Jiangxi Qingfeng Pharmaceutical Co., LTD., approved number of Chinese Medicine	time/day, adding 50-100ml glucose injection for intravenous drip. The children in the two groups were given rehydration, antipyretic and other symptomatic and supportive treatment. The clinical manifestations and adverse reactions of the two groups were observed and recorded closely.	was treated with Xiyanping injection on the basis of the control group, and the concentration of 5% normal saline and glucose injection were diluted, 2-4 ml/time, according to the doctor's advice. The patients were treated for 3 to 7 days according to their condition.	purifying agent, for traditional Chinese medicine have antibacterial, antiviral and antipyretic anti-inflammatory effect, by the qingfeng-xiangguang fracture pharmaceutical factory production in jiangxi province), dosage of 0.2 ~ 0.4 mL/kg · d, 50 g, join L glucose injection intravenous drip, was used in 5 o glucose injection tube and case mix with other drugs cause adverse reactions. The treatment duration was 5 days.	group was treated with Xiyanping injection (Jiangxi Qingfeng Pharmaceutical Co., LTD., specifications 50mg, 2ml). 5% glucose 100-250ml was added to intravenous drip, once a day. Both groups were given supportive symptomatic treatment, and the course of treatment was 3-5 days.	approval number Z20026249), taking 5~10mg/kg, diluted with 5% glucose injection 250ml, intravenous drip, drip speed control at 30~40 drops /min,1 time /d,5 days as a course of treatment.	to 7 days depending on the patient's condition.

Z20026249) was given intravenously, 10mg/kg each time, once a day for 7 days.

Outcomes	6a	Completely defined, prespecified primary and secondary outcome measures, including how and when they were assessed	recovery rate of disease,	recovery rate of disease, interleukin-8 (U/L), interleukin-6 (U/L)	recovery rate of disease, incidence of adverse events, the time of recovering normal temperature, duration of sore throat, recovery time of white blood cells,	recovery rate of disease, duration of sore throat, the time of tonsil purulent discharge, the time of tonsil purulent discharge, duration of disappearance of tonsillar redness and swelling	the time of recovering normal temperature, duration of sore throat, duration of disappearance of tonsillar redness and swelling, the time of tonsil purulent discharge, length of hospitliztion,	Primary and secondary outcome measures were not prespecified.	recovery rate of disease, the disappearance rate of tonsillar redness and swelling, the recovery rate of white blood cell count, , the disappearance rate of tonsillar redness and swelling, the recovery rate of white blood cell count	recovery rate of disease,
	6b	Any changes to trial outcomes after the trial commenced, with reasons	No important changes to methods after trial commencement.							

Sample size	7a	How sample size was determined	No specific sample size determination method was recorded.
	7b	When applicable, explanation of any interim analyses and stopping guidelines	Not Applicable
Randomization Sequence generation	8a	Method used to generate the random allocation sequence	All included studies were RCTs, thirteen trials (Qiao et al., 2015; Mai, 2018; Ren, 2018; Liu, 2020; Sun, 2020; Peng et al., 2015; Li, 2017; Shi, 2018; Luo, 2016; Hu et al., 2018; Dong, 2009; Ou et al., 2017; Liu et al., 2008; Gan et al., 2020) report the specific method of randomization. One trial (Sun, 2020) used the stratified randomization. Eight trials (Qiao et al., 2015; Ren, 2018; Liu, 2020; Peng et al., 2015; Li, 2017; Shi, 2018; Luo, 2016; Ou et al., 2017) used random number tables. Three trials (Mai, 2018; Hu et al., 2018; Gan et al., 2020) used treatment modalities. Three studies were categorized according to treatment modalities. Three trials were grouped by treatment modalities. One study (Dong, 2009) described using odd or even admission number sequence.
	8b	Type of randomization; details of any restriction (such as blocking and block size)	No specific type of randomisation was recorded.

Allocation concealment mechanism	9	Mechanism used to implement the random allocation sequence (such as sequentially numbered containers), describing any steps taken to conceal the sequence until interventions were assigned	No specific mechanism used to implement the random allocation sequence was recorded.
Implementation	10	Who generated the random allocation sequence, who enrolled participants, and who assigned participants to interventions	No specific method used to generate the random allocation sequence was recorded.

Blinding	11a	If done, who was blinded after assignment to interventions (for example, participants, care providers, those assessing outcomes) and how	No specific blinding protocol was recorded.							
	11b	If relevant, description of the similarity of interventions	Not relevant.							
Statistical methods	12a	Statistical methods used to compare groups for primary and secondary outcomes	No specific statistical analysis method was recorded.	All data were analyzed by SPSS18.0 system software. Measurement data t test; Count data x2 test; P < 0.05 was considered to indicate	All data were analyzed by SPSS10.0 statistical software, measurement data were expressed as mean ± standard deviation	SPSS17.0 software was used to process the data. Count data were expressed as [n (%)], and chi-square test was used. Measurement data were expressed as (x±s) and	No specific statistical analysis method was recorded.	No specific statistical analysis method was recorded.	SPSS19.0 statistical software was used to analyze the data. Measurement data were expressed as mean ± standard deviation	In this study, the statistical software used by the author to analyze the data was SPSS 19.0. The measurement data were expressed by chi-square

				statistical significance.	(x±s), measurement data between groups were compared by t test, count data were compared by X2 test, P<0.05 was considered statistically significant.	analyzed by t test. P < 0.05 was considered statistically significant.			(x±s), and t test was used. Count data were expressed as rate (%) and χ2 test was used. P<0.05 was considered statistically significant.	value, the t value was used for checking, the count data were expressed by x±s, and the count data were checked by χ2. When P < 0.05, the difference was statistically significant.
	12b	Methods for additional analyses, such as subgroup analyses and adjusted analyses	Subgroup and adjusted analyses were not performed.							
Results										
Participant flow (a diagram is strongly recommended)	13a	For each group, the numbers of participants who were randomly	See Table 1 for details.							

		assigned, received intended treatment, and were analyzed for the primary outcome	
	13b	For each group, losses and exclusions after randomization, together with reasons	No study reported the number of patients dropping out.
Recruitment	14a	Dates defining the periods of recruitment and follow-up	The follow-up time was the same as the treatment time, and the treatment time is shown in Table 1.
	14b	Why the trial ended or was stopped	Not applicable, none of the included studies were interrupted or stopped.

Baseline data	15	A table showing baseline demographic and clinical characteristics for each group	All the studies presented baseline data, including baseline demographic and clinical characteristics, for each group with the use of a table or text description.
Numbers analyzed	16	For each group, number of participants (denominator) included in each analysis and whether the analysis was by original assigned groups	The analysis was by original assigned groups.
Outcomes and estimation	17a	For each primary and secondary outcome, results for each	Not listed.

		group, and the estimated effect size and its precision (such as 95% confidence interval)	
	17b	For binary outcomes, presentation of both absolute and relative effect sizes is recommended	Not listed.
Ancillary analyses	18	Results of any other analyses performed, including subgroup analyses and adjusted analyses, distinguishing prespecified from exploratory	Subgroup and adjusted analyses were not performed.

Harms	19	All important harms or unintended effects in each group (for specific guidance, see CONSORT for harms [28])	See Table 1 for details.
Discussion			
Limitations	20	Trial limitations; addressing sources of potential bias; imprecision; and, if relevant, multiplicity of analyses	There were no trial limitations, addressing sources of potential bias and imprecision.

Generalizability	21	Generalizability (external validity, applicability) of the trial findings	The trial demonstrated the efficacy and safety of XYP combined with β-lactams in the treatment of children with SAT.	The trial demonstrated the efficacy and safety of XYP combined with Clindamycin hydrochloride injection in the treatment of children with SAT.	The trial demonstrated the efficacy and safety of XYP combined with β-lactams in the treatment of children with SAT.	The trial demonstrated the efficacy and safety of XYP combined with Ribavirin in the treatment of children with SAT.	The trial demonstrated the efficacy and safety of XYP combined with β-lactams in the treatment of children with SAT.	The trial demonstrated the efficacy and safety of XYP combined with β-lactams in the treatment of children with SAT.	The trial demonstrated the efficacy and safety of XYP combined with β-lactams in the treatment of children with SAT.	The trial demonstrated the efficacy and safety of XYP combined with Ribavirin in the treatment of children with SAT.

Interpretation	22	Interpretation consistent with results, balancing benefits and harms, and considering other relevant evidence	Suppurative acute tonsillitis in children is knowned as “Lan Ru E” in chinese. The onset of SAT is rapid, and it is mostly caused by the invasion of tonsil by fire and heat evil in theory of traditional Chinese medicine (TCM). TCM andrographis paniculata is the component of XYP. The efficacy of Chuan Xin Lian (CXL) is to clear heat and detoxify, cool blood, and detumescence. CXL can be used for the treatment of cold and fever, sore throat, mouth and tongue sores, cough, diarrhea and dysentery, hot and astringent pain, carcarols and sores, snake bites. Along with clinical use, the activity and mechanism of XYP has also been evaluated in experimental model systems. Investigations by Fan et al. showed that XYP, a new candidate of antiviral drug for DENV infection, can inactivate DENV2 and inhibit the entry of DENV2 into cells. XYP can significantly inhibit the replication of DENV2 in a dose-dependent manner and reduce the viral load in organs and sera, and improve the survival rate of experimental mice. The antivirus activity of XYP is superior to ribavirin injection in the early and late stage of virus infection (Fan et al., 2020). In terms of safety, investigations by Yu et al. showed that compared with the control group, drugs in the group of XYP for injection 400mg/kg, 200mg/kg and 100mg/kg, XYP had no significant effect on the central nervous systems, cardiovascular and respiratory systems of experimental animals (Yu et al., 2009). Investigations by Liu et al. showed that actual phagocytosis rate and phagocytic index of neutrophils in peripheral blood, phagocytosis rate and phagocytic index of macrophages in enterocoelia of blank group and cefazolin group were statistically significantly lower than XYP group and XYP combined with cefazolin group. XYP combined with cefazolin can promote the recovery of cytophagocytosis of peripheral blood neutrophils and enterocoelia macrophages in mice with Staphylococcus aureus infection, and then enhance the body’s anti-infection ability and antibacterial effect (Liu et al., 2015). An animal experiment demonstrated that the actual phagocytotic rate and index of peripheral blood neutrophils in XYP group and the XYP combination of Cefazolin group were significantly higher than those of the Cefazolin group, suggested the combination of XYP and Cefazolin could enhance the therapeutic effect by improving the phagocytic function of peripheral blood neutrophils (Xiong et al., 2015). Investigations by Xiong et al. showed that XYP combined with cafezoin sodium may promote the apoptosis of neutrophils and alleviate inflammation of mice with infection by reducing the level of IL-6 and improving the level of IL-10 in plasma (Xiong et al., 2020).
Other information			
Registration	23	Registration number and name of trial registry	The clinical trial was not registered.
Protocol	24	Where the full trial protocol can be	Not Applicable.

		accessed, if available								
Funding	25	Sources of funding and other support (such as supply of drugs), role of funders	There was no funding or other support for this study.	There was no funding or other support for this study.	There was no funding or other support for this study.	There was no funding or other support for this study.	Research Project Supported by Guangdong Province Building a strong Province of Traditional Chinese Medicine (No. 20131228)	There was no funding or other support for this study.	There was no funding or other support for this study.	There was no funding or other support for this study.
